# Bright V‐Shaped *bis*‐Imidazo[1,2‐*a*]pyridine Fluorophores with Near‐UV to Deep‐Blue Emission

**DOI:** 10.1002/asia.202200903

**Published:** 2022-10-26

**Authors:** Elise Jouaiti, Valerio Giuso, Damien Cianfarani, Nathalie Kyritsakas, Christophe Gourlaouen, Matteo Mauro

**Affiliations:** ^1^ Institut de Physique et Chimie des Matériaux de Strasbourg UMR7504 Université de Strasbourg CNRS 23 rue du Loess 67034 Strasbourg France; ^2^ Service de Radiocristallographie Fédération de chimie Le Bel – FR2010 BP 296R8 1, rue Blaise Pascal 67008 Strasbourg France; ^3^ Laboratoire de Chimie Quantique Institut de Chimie de Strasbourg UMR7177 Université de Strasbourg CNRS 4 Rue Blaise Pascal 67008 Strasbourg France

**Keywords:** N-heterocycles, fluorescence, density functional theory, fluorescent probes, chromophores

## Abstract

Ten novel small‐molecule fluorophores containing two electron‐accepting imidazo[1,2‐*a*]pyridine (ImPy) units are presented. Each ImPy core is functionalized at its C6 position with groups featuring either electron accepting (A) or donating (D) properties, thus providing emitters with general structure X−ImPy−Y−ImPy−X (X=either A or D; Y=phenyl or pyridine). The molecules bear either a phenyl (series **4**) or a pyridine (series **5**) π bridge that connects the two ImPys via *meta* (phenyl) or 2,6‐ (pyridine) positions, yielding an overall V‐shaped architecture. The final compounds are synthetized straightforwardly by condensation between substituted 2‐aminopyridines and α‐halocarbonyl derivatives. All the compounds display intense photoluminescence with quantum yield (PLQY) in the range of 0.17–0.51. Remarkably, substituent effect enables tuning the emission from near‐UV to (deep‐)blue region while keeping Commission Internationale de l’Éclairage (CIE) *y* coordinate ≤0.07. The emitting excited state is characterized by a few nanoseconds lifetime and high radiative rate constant, and its nature is modulated from pure π‐π* to intramolecular charge transfer (ICT) by the electronic properties of the peripheral X substituent. This is further corroborated by the nature of the frontier orbitals and vertical electronic excitations computed at (time‐dependent) density functional level of theory (TD‐)DFT. Finally, this study enlarges the palette of bright deep‐blue emitters based on the interesting ImPy scaffolds in view of their potential application as photo‐functional materials in optoelectronics.

## Graphical abstract

**A novel class of bright V‐shaped chromophores** is presented that feature imidazo[1,2‐*a*]pyridine units. The emitters possess an overall X−ImPy−π−ImPy−X architecture, where X=either electron‐acceptor or electron‐donor group. Photoluminescence is modulated from the near‐UV to the deep‐blue region by the electronic character of the peripheral groups that also influence the nature of the emitting excited state.

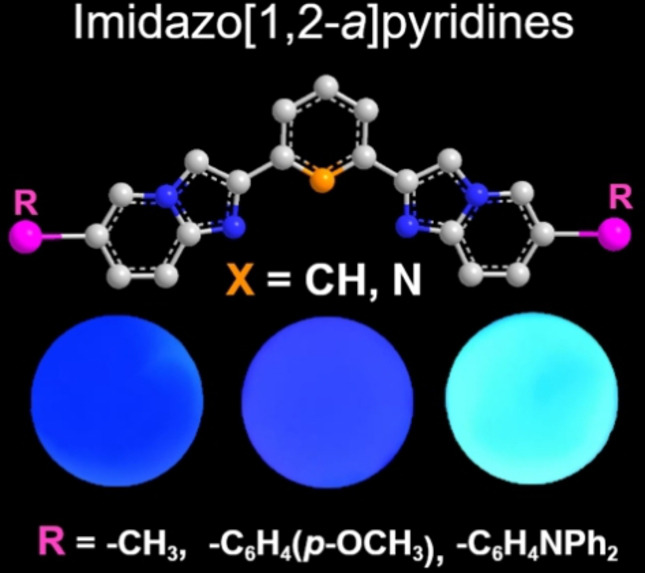

## Introduction

Molecular fluorophores have attracted great interest due to their excellent optical properties, such as high photoluminescence quantum yield, short‐lived excited state and emission wavelength tunable over the entire visible spectrum and beyond. This features have also prompted their application as photo‐functional materials in the field of laser dyes,^[1]^ bioimaging probes,[^2^, ^3^] and electroluminescent compounds in optoelectronics,^[4]^ just to cite some. Amongst the different classes of small‐molecule emitters, the imidazopyridine (ImPy) scaffolds represent an interesting family of N‐containing heterocycles, whose chemistry has been mainly developed in view of their application as pharmaceutical active species.^[5]^ These compounds have indeed demonstrated a large spectrum of biological activities and they are active principles in a few commercial drugs as well. From electronic viewpoint, the ImPy features a rigid planar scaffold that possesses two endocyclic *sp* ^2^‐hybridized nitrogen atoms, which confers appealing electron‐accepting features.

Imidazo[1,5‐*a*]pyridines and their coordination compounds are one of the most investigated ImPy isomers with optical properties.[^6^, ^7^, ^8^, ^9^, ^10^, ^11^, ^12^, ^13^, ^14^, ^15^] Instead, design and investigation of luminophores containing the parental imidazo[1,2‐*a*]pyridine derivatives have been limited to date. Pioneering investigation on their luminescence properties at various pH was reported by Leonard.^[16]^ Tomoda, Araki and co‐workers studied the substituent effect onto the imidazo[1,2‐*a*]pyridine core.^[17]^ In their study, modulation of the electronic properties was exerted by introducing groups at the 4’ position of substituted phenyl‐ring grafted at the C2 site of the ImPy. More recently, Yoo and co‐workers systematically investigated the effect of substitution at the C3 and C5 position on the imidazolyl and pyridyl scaffold, respectively.^[18]^ Also, a few studies focused on the investigation of the excited‐state intramolecular proton transfer (ESIPT) properties of 2′‐(2′‐hydroxyphenyl)imidazo[1,2‐*a*]pyridine derivatives both in solution and in the solid state.[^19^, ^20^, ^21^, ^22^, ^23^] Moreover, this class of organic fluorophore demonstrated interesting non‐linear optics response, such as near‐infrared (NIR) two‐photon absorption.^[24]^

Noteworthy, the use of emitters featuring the imidazo[1,2‐*a*]pyridine core in efficient organic light‐emitting diodes (OLEDs) was recently demonstrated and state‐of‐the‐art deep‐blue emitting devices with high brightness were fabricated.^[25]^

In this framework, it is worth noticing that the design of materials that matches the National Television Standards Committee (NTSC) for the deep‐blue color, *i. e*. CIE(x,y)=(0.14, 0.08) and that retain CIE_y_ ≤0.08 at high brightness is of paramount importance in OLED display technology and it is still highly challenging in the field of small molecule photo‐functional materials.^[26]^

Aiming at expanding the palette of bright deep‐blue fluorophores based on the appealing ImPy motifs, we hereafter describe the synthesis, crystallographic and photophysical investigation of a newly designed family of V‐shaped emitters. These latter contain two imidazo[1,2‐*a*]pyridine scaffolds that are functionalized at the C6 position with either electron‐donating (D) or electron‐accepting (A) peripheral groups. The molecular design was inspired by the fact that substituent effect at the C2 position was limited to the introduction of (substituted) aromatic rings in the literature, whereas substitution at the C6 position was largely overlooked to date. In the herein proposed molecular systems, connection between the two imidazo[1,2‐*a*]pyridine moieties is warrant by a π‐bridging unit (Y) such as either a phenyl (series **4**) or a pyridine (series **5**) linked at either meta (phenyl) or 2, 6 (pyridine) positions. Hence, emitters feature the general structure X−ImPy−Y−ImPy−X, where X is either A or D. Finally, the optical properties of these appealing luminophores are further elucidated by in‐depth investigation of the molecular frontier orbitals and vertical transition by means of density functional theory (DFT) and time‐dependent DFT.

## Results and Discussion

### Synthesis

**Scheme 1:**
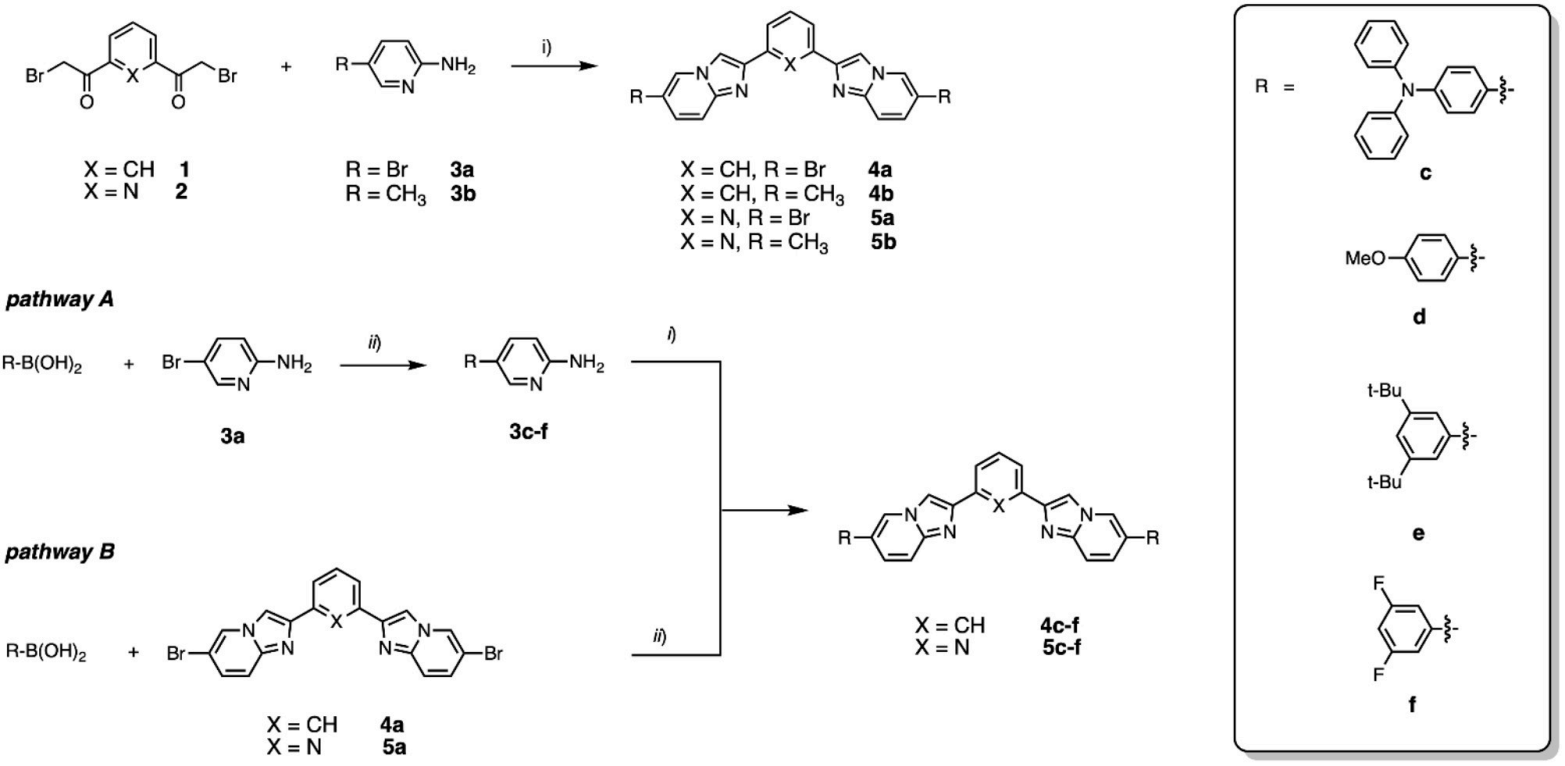
*i*) NaHCO_3_, CH_3_CN, reflux, overnight; *ii*) Pd(PPh_3_)_4_, NaHCO_3,_ 1,4‐dioxane:H_2_O 1 : 1, reflux, overnight.

The overall synthetic strategy employed for the preparation of the target compounds **4 b**–**f** and **5 b**–**f** is depicted in Scheme 1. For all the intermediates and target compounds, ^1^H, ^13^C and (when applicable) ^19^F NMR spectra are available in Figure S1–S35 of the Supporting Information and additional synthetic and characterization details are provided in the Experimental section (see below).

Condensation reaction between either α,α′‐dibromo‐1,3‐diacetylbenzene (**1**) or α,α′‐dibromo‐2,6‐diacetylpyridine (**2**) with 2‐amino‐5‐bromopyridine (**3 a**) yielded almost quantitatively intermediate derivative 1,3‐bis(6‐bromoimidazo[1,2‐*a*]pyridin‐2‐yl)benzene (**4 a**) and 2,6‐bis(6‐bromoimidazo[1,2‐*a*]pyridin‐2‐yl)pyridine (**5 a**), respectively, which possess poor solubility in different organic solvents regrettably. The use of 2‐amino‐5‐methylpyridine, namely **3 b**, under similar reaction condition provided 1,3‐bis(6‐methylimidazo[1,2‐*a*]pyridin‐2‐yl)benzene (**4 b**) and 2,6‐bis(6‐methylimidazo[1,2‐*a*]pyridin‐2‐yl)pyridine (**5 b**) in a straightforward manner. The presence of a bromine atom onto the aminopyridine **3 a** enabled widening of the palette of target imidazo[1,2‐*a*]pyridines by introducing moieties with different electronic properties (pathway A in Scheme 1). Reaction of **3 a** with boronic acid R−B(OH)_2_ under Pd‐catalyzed Suzuki‐Miyaura cross‐coupling condition yielded 5‐substituted 2‐amino‐pyridine **3 c**–**f**. Subsequently, condensation reaction between **3 c**–**f** and either α,α′‐dibromo‐1,3‐diacetylbenzene or α,α′‐dibromo‐2,6‐diacetylpyridine yielded the desired compounds **4 c**–**f** and **5 c**–**f**, respectively, in excellent (>70%) yield.

Aiming at comparing the different reaction condition of these interesting molecules, an alternative synthetic pathway was tested as well, namely pathway B in Scheme 1. This second strategy involves Suzuki‐Miyaura cross‐coupling between boronic acids and bromo‐derivatives (either **4 a** or **5 a**). Regrettably, this alternative method provided more complex product mixtures that were difficult to purify by column chromatography, most likely due to the poor solubility of brominated compound **4 a** or **5 a**. Overall, pathway B afforded target compounds with much lower yield compared to pathway A.

Single‐crystals of compound **4 c** and **5 b** of quality suitable for X‐ray diffractometric analysis were grown by slow diffusion of diethyl ether in CH_2_Cl_2_ solution and slow evaporation of a CH_2_Cl_2_:EtOH mixture, respectively. Their structural characterization helped to unambiguously confirm their atom connectivity and crystalline arrangement. Atom numbering and crystallographic data are provided in Figure S36–S37 and Table S1–S4 of the Supporting Information, respectively. As far as derivative **4 c** is concerned, the compound crystalizes into a *P*‐1 space group with adjacent molecules arranged into skewed head‐to‐tail packing. In the crystal, the central aromatic moieties adopts an almost planar conformation being the dihedral angle ∠C(27)−C(26)−C(22)−N(3) and ∠C(27)−C(28)−C(32)−N(4) equal to 0.08 and −17.98°, respectively. N1 atoms, each belonging to one of two pentatomic rings, are found to point inwards. Expectantly, the two peripheral triphenylamine pendants display a propeller‐like conformation.

Molecules **5 b** crystallizes into a *P*21/n space group and adopts an almost planar arrangement with dihedral angle ∠N(3)−C(9)−C(6)−C(7) and ∠N(3)−C(13)−C(14)−C(20) value of 6.44 and −0.53, respectively. Interestingly, two intermolecular C−H⋅⋅⋅π interactions with *d*(C‐centroid)=3.496 and 3.593 Å are present between neighboring molecules and involving the ImPy moieties (see Figure S37 of the Supporting Information).

### Photophysical properties

The optical properties of the two series of compounds have been investigated in dilute CH_2_Cl_2_ solution at room temperature, except for compound **5 f** that has been characterized in DMSO instead, due to its limited solubility in chlorinated solvents. The photophysical data are summarized in Table 1 and the corresponding absorption and photoluminescence spectra are depicted in Figure 3 and 4, respectively, for both series of investigated compounds.

**Table 1:** Photophysical data recorded for samples of the two series of compounds **1 b**–**f** and **2 b**–**f** in dilute solution at room temperature.

compound	λ_abs_ (ϵ) [nm, 10^4^ M^−1^ cm^−1^]	λ_em_ [nm]	Chromaticity CIE1931 (x, y)	PLQY	τ [ns]	*k*_r_ [10^8^ s^−1^]^[c]^	*k*_nr_ [10^8^ s^−1^]^[c]^
**4 b** ^[a]^	251 (6.17) 316 (1.76) 327 (1.76)	363, 378, 395*sh*	(0.17, 0.06)	0.41	2.91	1.41	2.03
**4 c** ^[a]^	253 (4.69) 310 (4.30) 332 (4.50)	425	(0.17, 0.06)	0.50	2.53	1.98	1.98
**4 d** ^[a]^	276 (4.68) 335*sh* (1.09)	392	(0.17, 0.05)	0.21	4.43 (26%) 2.58 (74%)	–	–
**4 e** ^[a]^	273 (6.80) 322 (1.41) 336*sh* (1.32)	392	(0.17, 0.04)	0.35	3.11	1.13	2.09
**4 f** ^[a]^	275 (8.89) 338 (1.56)	410	(0.16, 0.05)	0.24	3.43	0.70	2.22
**5 b** ^[a]^	321 (1.39) 331*sh* (1.49)	363, 378, 395*sh*	(0.17, 0.06)	0.32	2.97	1.08	2.29
**5 c** ^[a]^	252 (4.68) 310 (4.03) 340 (5.14)	431	(0.16, 0.06)	0.51	2.88	1.77	1.70
**5 d** ^[a]^	270 (6.50) 335 (2.42)	391	(0.18, 0.07)	0.25	4.48 (16%) 2.71 (84%)	–	–
**5 e** ^[a]^	264 (4.96) 334*sh* (1.83)	391	(0.17, 0.04)	0.30	2.84	1.06	2.46
**5 f** ^[b]^	330 (1.57)	405	(0.17, 0.07)	0.20	3.39	0.59	2.36

[a] in CH_2_Cl_2_. [b] in DMSO. [c] *k*_r_ and *k*_nr_ were estimated by using the following equations *k*_r_=PLQY/τ and *k*_nr_=(1–PLQY)/τ; *sh* denotes a shoulder.

The absorption spectra display two bands falling in the UV portion of the spectrum with a tail extending into the visible for compound **4 c** and **5 c** only. At shorter wavelengths (λ_abs_=*ca*. 250–270 nm), the spectrum is characterized by an intense (ϵ=4.7–8.9×10^4^ M^−1^ cm^−1^) and narrow band attributable to transition with largely spin‐allowed ^1^π–π* character and involving the whole molecule. This transition is indeed barely visible for the methylated derivative at the C6 position of the imidazo[1,2‐*a*]pyridine core, namely compound **4 b** and **5 b**, due to the smaller conjugation length of the two chromophores that lack of lateral (substituted) phenyl rings. At longer wavelength, a broader and less intense (ϵ=1.1–1.8×10^4^ M^−1^ cm^−1^) band is present that is attributed to the convolution of electronic transitions with admixed singlet‐manifold ^1^π–π* and intramolecular charge‐transfer (^1^ICT) nature. When going from a more electro‐donating *p*‐methoxy‐ to the electron‐withdrawing *meta*‐difluoro substituent of the peripheral aromatic rings (*i. e*. substituent **d**→**e**→**f**) the absorption onset shifts to longer wavelength monotonically, thus further corroborating the partial CT nature of the transition. Compound **4 c**–**5 c** do not follow this trend. The presence of a strong electro‐donating Ph_2_N‐ groups imparts an almost pure ICT character to the transition. Overall, it is possible to notice that the nature of the central substituted ring that bridges the two imidazo[1,2a]pyridine cores via substitution at the two *meta* positions, *i. e*. phenyl vs pyridine, affects the absorptions spectral feature to a minor extent only.

As shown in Figure 4, upon photoexcitation at λ_exc_=300–330 nm, all the compounds display intense near‐UV to blue fluorescence in diluted air‐equilibrated samples at room temperature, whose emission maximum parallels the trend observed in the absorption profiles.

Indeed, compound **4 b** and **5 b** display a largely similar structured emission profile with maximum centered at 378 nm and with PLQY values of 0.41 and 0.32, respectively. The photoluminescence spectrum of these compounds is the most hypsochromically shifted within the series and agrees well with those reported previously for the parental 2‐phenylimidazo[1,2‐*a*]pyridine.^[17]^ Introduction of the two additional substituted phenyls at position C6 yields a bathochromic shift of the emission, which is accompanied by a broadening of the spectral profile when going from R=di‐*tert*‐butylphenyl to difluorophenyl, being the emission maximum centered at λ_em_=391, 410 and 405 nm for **4 e**–**5 e**, **4 f** and **5 f**, respectively. PLQY slightly decreases from 0.35–0.3 to 0.24–0.2 when moving from **4 e**–**5 e** to **4 f**–**5 f**.

Once again, fluorophore **4 c** and **5 c** show a different behavior, due to the presence of the Ph_2_N− moieties, with emission falling in the deep‐blue region at 425 and 430 nm, respectively. Remarkably, this is accompanied by a strong increase of the PLQY up to 0.51.

Time‐resolved emission decays help to get better insight onto the nature and properties of the excited state responsible of the strong fluorescence in this class of compounds. Samples of all the investigated derivatives in solution, except for **4 d** and **5 d**, show emission decay traces that can be fitted with a mono‐exponential kinetics, providing excited‐state lifetime in the order of 2.53–5.94 ns. Rather similar radiationless rate constant values are determined falling in the range *k*_nr_=1.7–2.5×10^8^ s^−1^ that are indicative of the fact that non‐radiative emission channels have similar efficiency within the two families of emitters. Fluorophores **4 c** and **5 c** possess the largest radiative rate constant, *k*_r_, with values of 1.98 and 1.77×10^8^ s^−1^, respectively. On the other hand, slightly smaller *k*_r_ values are obtained for derivatives **4 f** and **5 f** indicative of the high degree of allowness of the radiative transitions with mainly ^1^π–π* nature. Furthermore, derivatives **4 c** and **5 c** are expected to possess an emitting excited state with a sizeable ^1^ICT, in accordance with the slightly broader emission spectra observed for these compounds. On the other hand, methoxy‐derivative **4 d** and **5 d** display a different behavior with a bi‐exponential emission decay most likely due to their reduced photothermal stability that has been observed during the synthesis and characterization.

The change in the nature of the excited state between series **b** and **c** is further highlighted by the remarkably different fluorosolvatochromism recorded for compound **4 b** and **4 c**, that have been chosen as examples. The spectra recoded in solvents with different polarity are displayed in Figure S38 and the corresponding data are listed in Table S5. While the former compound shows very minor variation of the emission profile and energy, indicative of a highly localized excited state (LE), the latter displays a clear positive fluorosolvatochromism pointing toward a mainly ^1^ICT character.

### Computational investigation

**Scheme 2:**
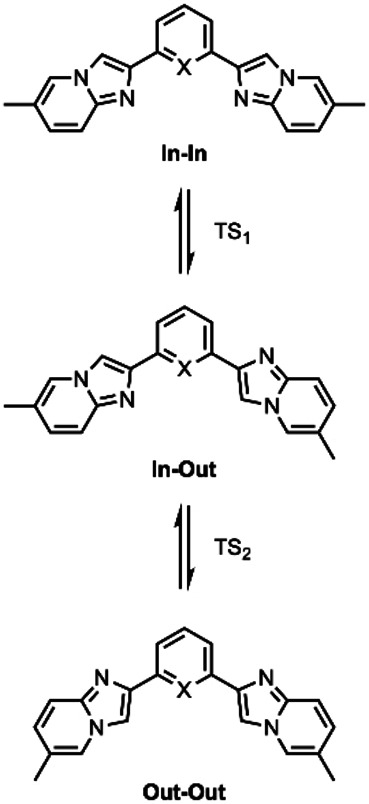
Name and structure of the different conformers of compound **4 b** and **5 b**, as an example.

Firstly, structural flexibility of compounds **4 b** and **5 b** was explored to determine the energetics of the different minimum‐energy conformations and the barrier of interconversion between them. Indeed, depending on the orientation of the imidazo[1,2‐*a*]pyridine, three conformers can be sketched, namely In−In, In‐Out and Out‐Out, which are depicted in Scheme 2 and energetic data are listed in Table S6–S13 of the Supporting Information. For **4 b**, and for compounds of series **4** in general, the three conformers are almost degenerate (Table 2 and Figure S40–S41) and are expected to be in fast equilibrium in solution at room temperature, due to the low energy barrier of interconversion (<6 kcal mol^−1^). For the corresponding derivative bearing a central pyridine, namely **5 b**, conformer Out‐Out is computed to be significantly lower in energy than the two others expectantly. Hence, it is likely that it represents the predominant species in solution. Similar behavior is expected for all derivatives of series **5**.

**Table 2:** Relative Gibbs free energy (ΔG) of the different conformers and the transition state (TS, ΔG^≠^) computed for derivative **4 b** and **5 b**. Energy values are given in kcal mol^−1^.

compound	In‐In	TS_1_	In‐Out	TS_2_	Out‐Out
**4 b**	0.6	5.2	0.0	5.5	0.2
**5 b**	6.2	9.8	2.7	6.7	0.0

**Figure 1:**
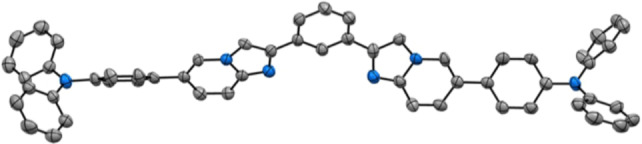
ORTEP diagram of compound **4 c** **⋅** **CH_2_Cl_2_** with thermal ellipsoids shown at 50% probability level obtained by single crystal X‐ray diffractometric analysis. Hydrogen atoms and CH_2_Cl_2_ solvent molecule are omitted for clarity. Gray and blue ellipsoids represent carbon and nitrogen atoms, respectively.

**Figure 2:**
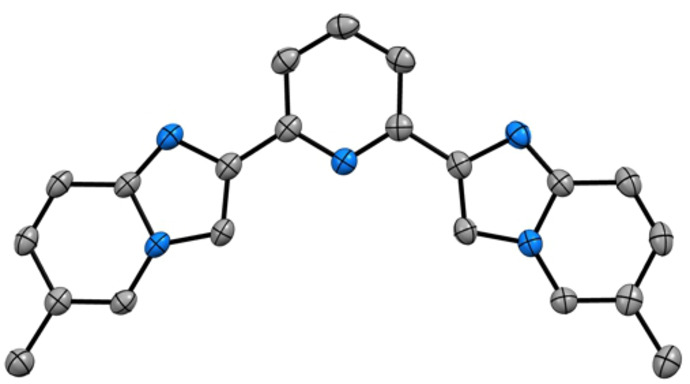
ORTEP diagram of compound **5 b** with thermal ellipsoids shown at 50% probability level obtained by single‐crystal X‐ray diffractometric analysis. Hydrogen atoms are omitted for clarity. Gray and blue ellipsoids represent carbon and nitrogen atoms, respectively.

The computed absorption spectra of the different structures are displayed in Figures 5 and 6 and the data are listed in Table S14–S18 of the Supporting Information. We retained the In−In conformation for series **4** and the Out‐Out for series **5** in agreement with the experimental crystallographic data (Figures 1 and 2). The computation reproduces well the experimental findings. For **4 b**, the theoretical spectrum computed for the In−In conformer exhibits two bands, a first one centered at 320 nm and a second one peaking at 260 nm, which are in very good agreement with the experimental photophysical data (see above). These two bands are the results of the convolution of several, energetically close, electronic transitions. Three electronic transitions contribute to the band at λ_abs_=320 nm. All of them are π–π* in nature and are delocalized over the two imidazo[1,2‐*a*]pyridine and on the central phenyl ring. The higher‐energy band centered around 260 nm is the overall result of seven transitions with a major one at 265 nm that also possesses a π–π* character. This agrees well with the experimental spectra (Table 1), the two maxima observed at 316 and 328 nm are consistent with the values computed for the first band and that at 251 nm to the values computed for the second band.

**Figure 3:**
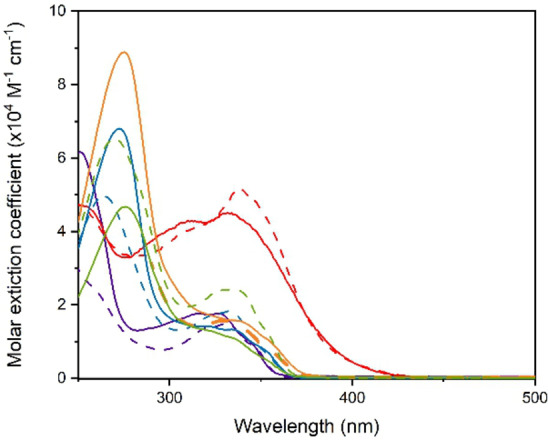
Electronic absorption spectra recorded for compounds of the series **4** (solid traces) and **5** (dashed traces) in diluted CH_2_Cl_2_ (DMSO for **5 f**) solution at room temperature. Color code: **b** (violet); **c** (red); **d** (green); **e** (blue); **f** (orange).

**Figure 4:**
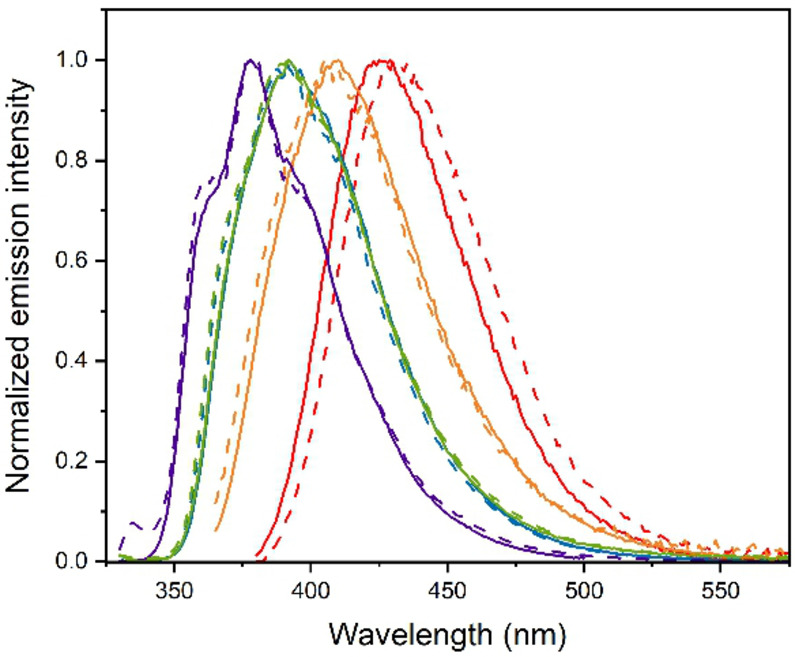
Photoluminescence spectra recorded for compounds of the series **4** (solid traces) and **5** (dashed traces) in diluted CH_2_Cl_2_ (DMSO for **2 f**) solution at room temperature upon excitation at λ_exc_=300–330 nm. Color code: **b** (violet); **c** (red); **d** (green); **e** (blue); **f** (orange).

**Figure 5:**
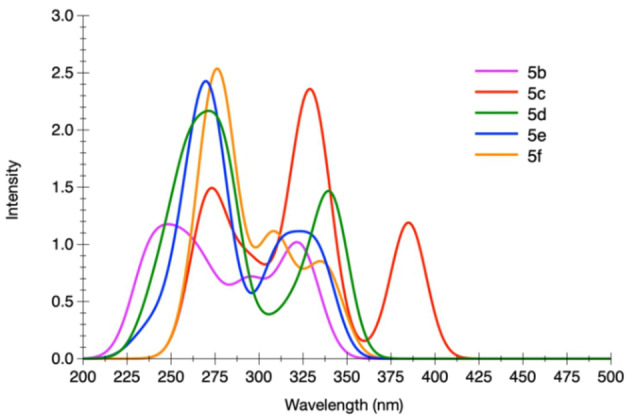
Electronic absorption spectra computed for compounds of series **4** in CH_2_Cl_2_.

**Figure 6:**
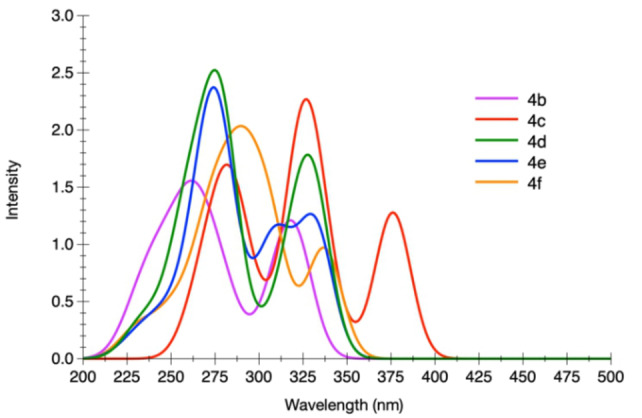
Electronic absorption spectra computed for compounds of series **5** in CH_2_Cl_2_.

As far as compound **5 b** is concerned, a first band is present at 322 nm in its computed spectrum, composed of three electronic transitions, consistent with the experimental maxima at 321 and 331 nm (see Table 1). A shoulder is present at 295 nm due to a single transition, with no obvious experimental counterpart. Yet, this latter can be present below the close and more intense band that is visible in the experimental spectrum. A large band is present around 250 nm, which is the result of the contribution of five intense transitions.

**Figure 7:**
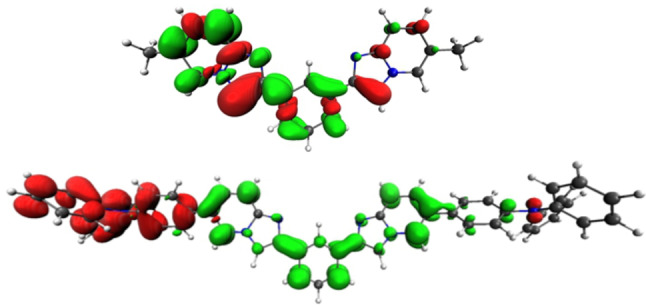
Electron density difference maps for the vertical transition S_1_→S_0_, computed at the S_1_ at the optimized geometry for **4 b** (top) and **4 c** (bottom). Electron density enriched and depleted areas are in green and red, respectively.

Similarly to what is observed for **4 b**, all the computed transitions for **5 b** can be described as excitations with large π–π* character involving different portions and to a different extent the π system of the molecule. A similar picture can be drawn for the other compounds within a given series, with the noticeable exception of derivatives **4 c** and **5 c**. Indeed, for these latter the nature of the lowest‐lying singlet‐manifold excitation process populates an excited state with ICT nature. This process can be described as an electron density moving from the peripheral *N*,*N*‐diphenylaniline moiety towards the fused imidazo[1,2‐*a*]pyridine core (Figure 7 and S39).

Secondly, the excited state and emission properties were investigated. The computed emission wavelengths are listed in Table 3 and the values are in excellent agreement with the experimental data (*cf*. Table 1).

**Table 3:** Computed emission wavelengths (in CH_2_Cl_2_) for the different investigated derivatives with the In−In and Out‐Out conformations for series **4** and **5**, respectively. These values are computed for the most stable structure without symmetry constraints (see Table S7–S13 for complete list). Data are listed in nm.

series	b	c	d	e	f
**4**	362	433	376	375	397
**5**	363	438	389	368	393

**Figure 8:**
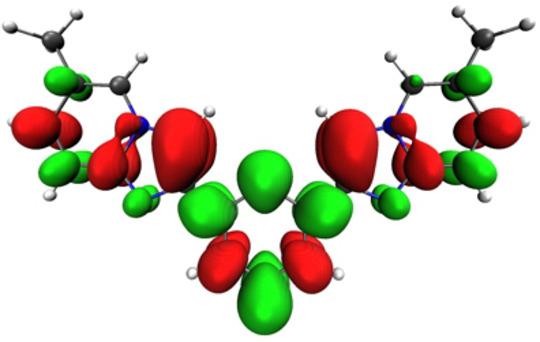
Electron density difference maps for the vertical transition S_1_→S_0_, computed at the S_1_ at the optimized geometry for **5 b** (bottom). Electron density enriched and depleted areas are in green and red, respectively.

The methylated derivatives **4 b** and **5 b** do not show any change in nature of the lowest excited singlet state, S_1_, from the Franck‐Condon (FC) state upon geometry relaxation. The emission wavelength for the S_1_→S_0_ transition is computed at 362 and 363 nm for compound **4 b** and **5 b**, respectively, being the experimental E_0,0_ value of emission 363 nm for both. This transition arises from the S_1_ state, which is similar in nature to that of the absorption process. However, a small difference appears between the phenyl (**4 b**) and the pyridine (**5 b**) containing derivative, but still consistent with the experimental findings. As for as derivative **4 b** is concerned, the excited state appears localized onto one of the two imidazo[1,2‐*a*]pyridine moieties only (Figure 7). Nevertheless, the barrier between the two degenerate, localized states, is computed to be only 0.048 eV (Figure S40–S41, Table S6). By comparing this value with the thermal energy at 300 K, being 0.026 eV, one may think that there is a fast excited state hopping between the two minima in solution at room temperature at the nanosecond time scale. On the other hand, emitting excited state S_1_ extends over the whole π system for derivative **5 b** (Figure 8).

A similar picture can be drawn for the emission of compounds **4 e** and **5 e**. The bathochromic shift observed experimentally is due to a minor contribution of the substituted phenyl that yields obvious extension of the π‐conjugated system. Indeed, the di‐*tert‐*butylphenyl groups contribute to the lower‐lying accepting virtual orbital and, as consequence, provide a CT character to the emitting state. As evidenced by the THEODore analysis, this CT contribution remains small and amounts for about 5–10% of the overall character of the electronic transition (Figure S39).

The nature of the lowest excited singlet state of **4 c** and **5 c** is similar to that of the S_1_ state computed for the absorption process at FC (see above). The emitting state can be described as having large CT character and involving an electron density redistribution from one of the *N*,*N*‐diphenylaniline group to the central core (*i. e*. the *bis*‐imidazo[1,2‐*a*]pyridine scaffold) of the molecule (Figure 7).

For structures **4 d**–**5 d** and **4 f**–**5 f**, the lowest singlet excited state at Franck‐Condon geometry is similar in nature to that computed for the corresponding methylated derivative, *i.e* **4 b** and **5 b** as depicted in Figure 7 and Figure 8, respectively. In sharp contrast to what observed for the other investigated derivatives, its nature changes upon geometry relaxation yielding a lower‐energy minimum on the S_1_ Potential Energy Surface (PES) with different character. During this relaxation, the excited state gains a partial CT character, whose extent depends on the electronic nature of the peripheral substituents.

**Figure 9:**
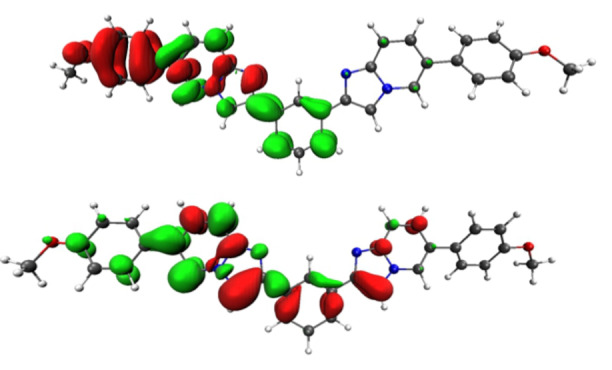
Electron density difference maps for the vertical transition S_1_→S_0_ for compound **4 d** for two different S_1_ PES local minima. Top image corresponds to the minimum with π–π* character and bottom image to the one with CT character. Electron density enriched and depleted areas are in green and red, respectively.

For **4 d**, the two local minima corresponding to the S_1_ with π‐π* and CT character are close in energy, but the former is found to be slightly more stable than the latter (see Figure 9 and Table S11). This picture is different for **5 d**, where the CT state lies well below the π‐π* state (Table S12). The CT involves an electron density redistribution from the more electron‐rich anisole moiety towards the *bis*‐imidazo[1,2‐*a*]pyridine central core (Figure 9).

**Figure 10:**
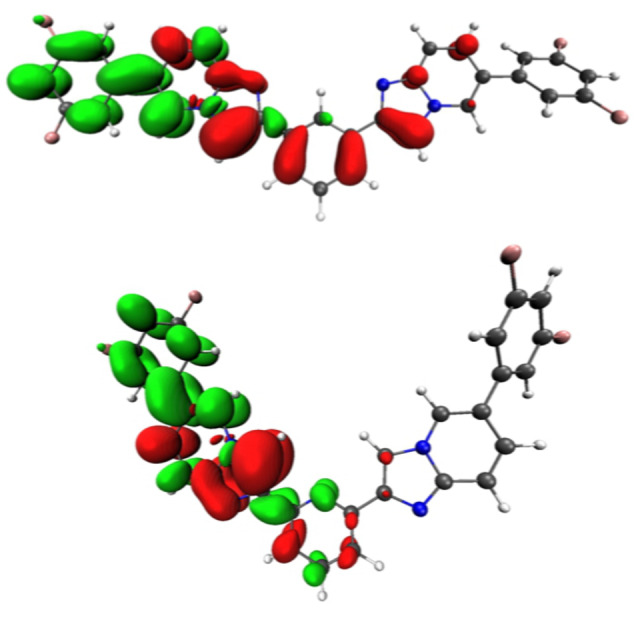
Electron density difference maps for the vertical transition S_1_→S_0_ for compound **4 f** (top) and **5 f** (bottom). Electron density enriched and depleted areas are in green and red, respectively.

For **4 f**–**5 f**, no minima were found corresponding π–π* state on the S_1_ PES. Given the electron‐withdrawing nature of the difluorophenyl moieties in compounds **4 f** and **5 f**, the direction of the electron density redistribution in the CT character of the optimized S_1_ is now reversed, with a transfer from the *bis*‐imidazo[1,2‐*a*]pyridine central core towards one of the two peripheral 3,5‐difuorophenyl groups (Figure 10). For these two compounds, no minima on the S_1_ PES were found corresponding π–π* state.

These results allow us to rationalize the experimental data. As a matter of fact, series **4** favors emission from localized excited state, whereas series **5** displays S_1_ with delocalized character (*cf*. Figure 7 and Figure 8).

Fluorophores **4 b** and **5 b** emits from a π–π* transitions of the core. A similar picture can be drawn for **4 e** and **5 e**. These latter possess peripheral phenyls substituted with moderate electron donating groups that yields a small bathochromic shift due to the extension of the π system.

Even at Franck‐Condon geometries, **4 c** and **5 c** are different, S_1_ being a CT state from the amine R group towards the core and after S_1_ geometry optimization this leads to the structure with the longest emission wavelength. A similar CT state appears for **4 d** and **5 d**, though the low stabilization of the CT state fortuity leads to similar emission wavelength than for **4 e** and **5 e**. Finally, **4 f** and **5 f** also emits from a CT state but in which the R group is electron accepting (Figure 10). These results show that in this family of structure it is possible by the change of the R group to modify the nature of the emitting state. Furthermore, this rough static description is probably complexified by the presence of rotamers allowed by the degenerated minima separated by low barriers (Table 2). This flexibility of the structural and electronic properties paves the way for accurate design of structures with selected properties.

## Conclusion

A novel family of V‐shaped fluorophores based on the imidazo[1,2‐*a*]pyridine core is presented and comprehensively characterized. The compounds differ in the nature of the π‐conjugated bridge (phenyl vs. pyridine). The emission properties are modulated in energy and lifetime by the electronic character of the peripheral substituents, being either an electron‐donating or withdrawing group. Similarly, the nature of the excited state is tuned from purely π‐π* to mainly ICT in character and also supported by computational investigation at TD‐DFT level. Remarkably, these emitters display intense fluorescence in the near‐UV to deep‐blue region with CIE y coordinate ≤0.07, which makes them a highly appealing class of emitters for deep‐blue electroluminescent devices and their application is organic light‐emitting diodes (OLEDs) is currently under investigation. Finally, their potential use as optical probes and molecular sensors (e. g. acids and metal ions) can be envisaged as well both in solution and as solid state materials.

## Experimental Section

### General considerations

2‐Amino‐5‐bromopyridine (**3 a**), 2‐amino‐5‐methylpyridine (**3 b**) were commercially available and used as received. Solvents and other commonly available reagents were purchased and used without further purification. α,α′‐Dibromo‐1,3‐diacetylbenzene (**1**) and α,α′‐dibromo‐2,6‐diacetylpyridine (**2**) were synthesized as reported in literature.[^27^, ^28^] Silica gel for column chromatography was purchased from Sigma‐Aldrich. ^1^H, ^13^C{^1^H} and ^19^F{^1^H} NMR spectra were recorded at 298 K on either Bruker AV500 spectrometer in deuterated solvents and the residual solvent peak was used as the internal reference. NMR spectra were calibrated to residual solvent signals. All the chemical shifts (δ) are reported in ppm. Mass spectrometry was performed by the Service Spectrométrie de Masse of the Fédération de Chimie “Le Bel” FR2010 of the University of Strasbourg.

### Synthesis

#### Synthesis of 5‐substituted 2‐aminopyridines 3 c‐f: general procedure.

A mixture of 2‐amino‐5‐bromopyridine (1.0 g, 5.7 mmol), NaHCO_3_ (1.4 g, 17.1 mmol) and the boronic acid derivative (6.84 mmol) in 45 mL of a mixture 1,4‐dioxane/H_2_O (1 : 1 ^v^/_v_) was degassed by steady bubbling with argon for 20 minutes. Pd(PPh_3_)_4_ (0.02 g, 0.018 mmol) was added, and the mixture was refluxed overnight under argon. After cooling the mixture, it was extracted with CH_2_Cl_2_ (3×50 mL). The combined organic layers were washed with brine, dried over MgSO_4_, and evaporated under vacuum. The residue was purified by silica gel column chromatography with CH_2_Cl_2_/MeOH mixture varying from 100:0 to 98 : 2 as eluent to provide the target compound **3 c**–**f**.

**5‐(4‐(diphenylamino)phenyl)pyridin‐2‐amine (3 c)**: white solid, 1.8 g, yield 92%. ^1^H NMR (CDCl_3_, 500 MHz): δ 8.28 (d, *J=*2 Hz, 1H), 7.66 (dd, *J_1_=*2 Hz, *J_2_=*8.5 Hz, 1H), 7.41 (d, *J=*8.5 Hz, 2H), 7.27 (t, *J=*8.5 Hz, 4H), 7.11–7.05 (m, 6H), 7.03 (t, *J=*8.5 Hz, 2H), 6.57 (d, *J=*8.5 Hz, 1H). ^13^C NMR (CDCl_3_, 126 MHz): δ 159.5, 149.6, 148.7, 147.8, 137.7, 134.4, 131.1, 128.7, 128.5, 126.2, 126.1, 124.7, 110.1. HR‐MS (ESI): m/z [M+H]^+^ calcd for C_23_H_20_N_3_ [M+H]^+^ 338.1652; found 338.1651.

**5‐(4‐methoxyphenyl)pyridin‐2‐amine (3 d)**: white solid, 1.0 g, yield 96%. ^1^H NMR (CDCl_3_, 500 MHz): δ 8.24 (d, *J=*2.5 Hz, 1H), 7.62 (dd, *J_1_=*2.5 Hz, *J_2_=*8.5 Hz, 1H), 7.4 (dt, *J_1_=*2.5 Hz, *J_2_=*8.5 Hz, 2H), 6.95 (dt, *J_1_=*2.5 Hz, *J_2_=*8.5 Hz, 2H), 6.55 (d, *J*=8.5 Hz, 1H), 4.52 (s, 2H), 3.82 (s, 3H). ^13^C NMR (CDCl_3_, 126 MHz): δ 158.9, 157.0, 145.5, 136.5, 130.7, 127.4, 127.2, 114.4, 108.7, 55.4. HR‐MS (ESI): m/z [M+H]^+^ calcd for C_12_H_13_N_2_O [M+H]^+^ 201.1022; found 201.1022.

**5‐(3,5‐di‐*tert*‐butylphenyl)pyridin‐2‐amine (3 e)**: white solid, 1.5 g, yield 93%. ^1^H NMR (CDCl_3_, 500 MHz): δ 8.30 (d, *J=*2 Hz, 1H), 7.66 (dd, *J=*2 Hz, *J=*8 Hz, 1H), 7.39 (s, 1H), 7.31 (d, *J*=2 Hz, 2H), 6.57 (d, *J*=8 Hz, 1H), 4.51 (s, 2H), 1.35 (s, 18H). ^13^C NMR (CDCl_3_, 126 MHz): δ 157.4, 151.3, 146.5, 137.6, 137, 128.6, 121.2, 120.9, 108.5, 35, 31.5. HR‐MS (ESI): m/z [M+H]^+^ calcd for C_19_H_27_N_2_ [M+H]^+^ 283.2169; found 283.2165.

**5‐(3,5‐difluorophenyl)pyridin‐2‐amine (3 f)**: white solid, 1.04 g, yield 88%. ^1^H NMR (CDCl_3_, 500 MHz): δ 8.26 (d, *J=*2 .5 Hz, 1H), 7.59 (dd, *J=*2.5 Hz, *J=*9 Hz, 1H), 7.01–6.95 (m, 2H), 6.71 (tt, *J*=2.5 Hz, *J*=8.5 Hz, 1H), 6.55 (d, *J*=9 Hz, 1H), 4.62 (s, 2H). ^13^C NMR (CDCl_3_, 126 MHz): δ 163.4 (dd, *J*_C‐F_ *=*13.3 Hz, *J*_C‐F_ *=*267.3 Hz), 158.4, 146.4, 141.6, 136.3, 125, 108.8 (dd, *J*_C‐F_ *=*6.0 Hz, *J*_C‐F_ *=*52.4 Hz), 108.5, 102.1 (t, *J*_C‐F_ *=*25.3 Hz). ^19^F NMR (CDCl_3_, 300 MHz): δ −109.5. HR‐MS (ESI): m/z [M+H]^+^ calcd for C_11_H_9_F_2_N_2_ [M+H]^+^ 207.0728; found 207.0732.

#### Synthesis of 4 a‐f and 5 a‐f: general procedure

The α,α′‐dibromo‐1,3‐diacetylbenzene or α,α′‐dibromo‐2,6‐diacetylpyridine (1.5 mmol) reacted with 2‐aminopyridine derivatives **1 a**–**f** respectively (3.0 mmol) and sodium hydrogen carbonate (9.0 mmol) in acetonitrile (40 mL) at reflux for 8 h. After cooling and filtration, the residue was washed successively with water, MeOH and petroleum ether and dried under vacuum.

**1,3‐bis(6‐bromoimidazo[1,2‐*a*]pyridin‐2‐yl)benzene (4 a)**: pale brown powder, 0.602 g, yield 86%. ^1^H NMR (CD_2_Cl_2_, 500 MHz): δ 8.57 (s, 1H), 8.4 (t, *J*=1 Hz, 2H), 8.04 (s, 2H), 7.97 (d, *J*=8.5 Hz, 2H), 7.56–7.54 (m, 3H), 7.3 (dd, *J*=1 Hz, *J*=8.5 Hz, 2H). ^13^C NMR (CD_2_Cl_2_, 126 MHz): δ 146.1, 144.1, 134.0, 129.2, 127.9, 125.9, 125.6, 123.5, 118.0, 108.7, 106.7. ESI‐MS: m/*z* [M+H]^+^ calcd for C_20_H_13_Br_2_N_4_ 466.9501, found 466.9507.

**1,3‐bis(6‐methylimidazo[1,2‐*a*]pyridin‐2‐yl)benzene (4 b)**: pale yellow solid, 0.432 g, yield 85%. ^1^H NMR (CDCl_3_, 500 MHz): δ 8.45 (s,1H), 7.92–7.89 (m, 6H), 7.53 (d, *J*=9 Hz, 2H), 7.48 (t, *J*=8 Hz, 1H), 7.00 (d, *J*=9 Hz, 2H), 2.31 (s, 6H). ^13^C NMR (CDCl_3_, 126 MHz): δ 145.4, 144.7, 134.3, 129.2, 127.9, 125.4, 123.4, 123.3, 122.0, 116.8, 108.3, 18.2. ESI‐MS: m/*z* [M+H]^+^ calcd for C_22_H_19_N_4_ [M+H]^+^ 339.1604 found 339.1603.

**4,4′‐(1,3‐phenylenebis(imidazo[1,2‐*a*]pyridine‐2,6‐diyl))bis(*N***, ***N*** **‐diphenylaniline) (4 c)**: pale brown solid, 1.016 g, 85% yield. ^1^H NMR (CDCl_3_, 500 MHz): δ 8.52 (s,1H), 8.26 (s, 2H), 8.02 (s, 2H), 7.96 (d, *J*=7.8 Hz, 2H), 7.69 (d, *J*=9 Hz, 2H), 7.52 (t, *J=*8 Hz, 1H), 7.44–7.40 (m, 6H), 7.27 (dd, *J_1_* =7 Hz, *J_2_* =6 Hz, 8H), 7.28–7.10 (m, 12H), 7.12 (t, *J*=7 Hz, 4H). ^13^C NMR (CDCl_3_, 126 MHz): δ 147.8, 147.5, 146.0, 144.8, 134.1, 132.1, 130.8, 129.4, 128.6, 127.5, 126.6, 125.7, 125.4, 124.6, 123.7, 123.3, 122.2, 117.2, 108.9. ESI‐MS: m/*z* [M+H]^+^ calcd for C_56_H_41_N_6_ [M+H]^+^ 797.3387 found 797.3357.

**1,3‐bis(6‐(4‐methoxyphenyl)imidazo[1,2‐*a*]pyridin‐2‐yl)benzene (4 d)**: pale yellow solid, 0.572 g, yield 73%. ^1^H NMR (CDCl_3_, 500 MHz): δ 8.53 (s,1H), 8.24 (s, 2H), 8.03 (s, 2H), 7.96 (d, *J*=7.75 Hz, 2H), 7.68 (d, *J*=9 Hz, 2H), 7.52–7.48 (m, 5H), 7.41 (d, *J*=9 Hz, 2H), 7.00 (d, *J*=6.5 Hz, 4H), 3.85 (s, 6H). ^13^C NMR (CDCl_3_, 126 MHz): δ 159.6, 145.7, 144.7, 134.0, 129.6, 129.3, 128.0, 126.8, 125.8, 125.7, 123.5, 122.3, 117.1, 114.6, 109.0, 55.4. ESI‐MS: m/z [M+H]^+^ calcd for C_34_H_27_N_4_O_2_ [M+H]^+^ 523.2129 found 523.2112.

**1,3‐bis(6‐(3,5‐di‐*tert*‐butylphenyl)imidazo[1,2‐*a*]pyridin‐2‐yl)benzene (4 e)**: pale brown solid, 0.897 g, yield 87%. ^1^H NMR (CDCl_3_, 500 MHz): δ: 8.55 (s,1H), 8.29 (s, 2H), 8.07 (s, 2H), 7.98 (d, *J*=7.5 Hz, 2H), 7.70 (d, *J*=9 Hz, 2H), 7.53 (t, *J*=7.5 Hz, 1H), 7.53–5.44 (m, 4H), 7.39 (s, 4H), 1.40 (s, 36H). ^13^C NMR (CDCl_3_, 126 MHz): δ 152.6, 151.7, 146.3, 145.0, 137.6, 136.7, 128.4, 126.3, 123.1, 122.2, 121.4, 119.4, 117.5, 111.5, 35.0, 31.5. ESI‐MS: m/*z* [M+H]^+^ calcd for C_48_H_55_N_4_ [M+H]^+^ 687.4421 found 687.4418.

**1,3‐bis(6‐(3,5‐difluorophenyl)imidazo[1,2‐*a*]pyridin‐2‐yl)benzene (4 f)**: pale brown solid, 0.682 g, yield 85%. ^1^H NMR (CDCl_3_, 500 MHz): δ 8.57 (s,1H), 8.34 (s, 2H), 8.08 (s, 2H), 8.01 (d, *J*=7.8 Hz, 2H), 7.75 (d, *J*=9.3 Hz, 2H), 7.57 (t, *J*=7.8 Hz, 1H), 7.41 (d, *J*=9.3 Hz, 2H), 7.13 (d, *J*=9 Hz, 4H), 6.87 (t, *J*=9 Hz, 2H). ^13^C NMR (CDCl_3_, 126 MHz): δ 163.5 (dd, *J*_C‐F_ *=*12.9 Hz, *J*_C‐F_ *=*262.7 Hz), 146.6, 145.0, 140.6 (t, *J*_C‐F_ *=*9.9 Hz), 134.0, 129.4, 126.0, 124.9, 124.6, 123.6, 123.3, 117.8, 109.9 (dd, *J*_C‐F_ *=*6.1 Hz, *J*_C‐F_ *=*26.2 Hz), 109.2, 103.0 (t, *J*_C‐F_ *=*25.3 Hz). ^19^F NMR (CDCl_3_, 300 MHz): δ −108.6. ESI‐MS: m/z [M+H]^+^ calcd for C_32_H_19_F_4_N_4_ [M+H]^+^ 535.1540 found 535.1525.

**1,3‐bis(6‐bromoimidazo[1,2‐*a*]pyridin‐2‐yl)pyridine (5 a)**: pale brown powder, 0.592 g, yield 84%. ^1^H NMR (CD_2_Cl_2_, 500 MHz): δ 8.42 (s, 2H), 8.37 (s, 2H), 8.1 (d, *J*=8 Hz, 2H), 7.9 (t, *J*=8 Hz, 1H), 7.5 (d, *J*=8 Hz, 2H), 7.3 (d, *J*=8 Hz, 2H). ^13^C NMR (CD_2_Cl_2_, 126 MHz): δ 152.2, 146.3, 144.1, 137.6, 128.3, 126.2, 119.5, 118.3, 111.2, 107.2. ESI‐MS: m/*z* [M+H]^+^ calcd for C_19_H_12_Br_2_N_5_ [M+H]^+^ 469.9434 found 469.9413.

**2,6‐bis(6‐methylimidazo[1,2‐*a*]pyridin‐2‐yl)pyridine (5 b)**: pale yellow solid, 0.418 g, yield 82%. ^1^H NMR (CDCl_3_, 500 MHz): δ 8.26 (s, 2H), 8.07 (d, *J*=7.5 Hz, 2H), 7.92 (s, 2H), 7.84 (t, *J*=7.5 Hz, 1H), 7.53 (d, *J*=10 Hz, 2H), 7.00 (d, *J*=10 Hz, 2H), 2.32 (s, 6H). ^13^C NMR (CDCl_3_, 126 MHz): δ 152.6, 145.6, 144.7, 137.5, 128.2, 123.5, 122.4, 119.2, 117.1, 110.8, 18.2. ESI‐MS: m/*z* [M+H]^+^ calcd for C_21_H_18_N_5_ [M+H]^+^ 340.1557 found 340.1546.

**4,4′‐(pyridine‐2,6‐diylbis(imidazo[1,2‐*a*]pyridine‐2,6‐diyl))bis(*N***, ***N*** **‐diphenylaniline) (5 c)**: slightly brown solid, 0.957 g, 80% yield. ^1^H NMR (CDCl_3_, 500 MHz): δ 8.41 (s, 2H), 8.30 (s, 2H), 8.12 (d, *J=*7.5 Hz, 2H), 7.88 (t, *J*=8 Hz, 1H), 7.70 (d, *J*=9.5 Hz, 2H), 7.45–7.41 (m, 6H), 7.28 (dd, *J_1_* =7.5 Hz, *J_2_* =7.5 Hz, 8H), 7.16–7.12 (m, 12H), 7.05 (t, *J=*7.5 Hz, 4H). ^13^C NMR (CDCl_3_, 126 MHz): δ 125.5, 147.9, 147.4, 146.1, 144.8, 137.6, 130.6, 129.4, 127.6, 126.9, 125.7, 124.7, 123.7, 123.3, 122.4, 119.5, 117.6, 111.4. ESI‐MS: m/*z* [M+H]^+^ calcd for C_55_H_40_N_7_ [M+H]^+^ 798.3340 found 798.3367.

**2,6‐bis(6‐(4‐methoxyphenyl)imidazo[1,2‐*a*]pyridin‐2‐yl)pyridine (5 d)**: pale yellow solid, 0.589 g, yield 75%. ^1^H NMR (CDCl_3_, 500 MHz): δ 8.63 (s,2H), 8.49 (s, 2H), 8.35 (d, *J*=8 Hz, 2H), 8.10 (t, *J*=8 Hz, 1H), 7.91 (d, *J*=9.5 Hz, 2H), 7.72 (d, *J*=9 Hz, 4H), 7.64 (d, *J*=9.5 Hz, 2H), 7.23 (d, *J*=9 Hz, 4H), 3.85 (s, 6H). ^13^C NMR (CDCl_3_, 126 MHz): δ 159.6, 152.4, 146.0, 144.7, 137.6, 129.6, 128.0, 127.0, 125.9, 122.4, 119.5, 117.5, 114.6, 111.5, 55.4. ESI‐MS: m/*z* [M+H]^+^ calcd for C_33_H_26_N_5_O_2_ [M+H]^+^ 524.2081 found 524.2087.

**2,6‐bis(6‐(3,5‐di‐*tert*‐butylphenyl)imidazo[1,2‐*a*]pyridin‐2‐yl)pyridine (5 e)**: pale brown solid, 0.908 g, yield 88%. ^1^H NMR (CDCl_3_, 500 MHz): δ 8.55 (s, 1H), 8.28 (s, 2H), 8.07 (s, 2H), 7.98 (d, *J*=8 Hz, 2H), 7.70 (d, *J*=9 Hz, 2H), 7.53 (t, *J*=8 Hz, 1H), 7.47–7.38 (m, 4H), 7.38 (s, 4H), 1.31 (s, 36H). ^13^C NMR (CDCl_3_, 126 MHz): δ 151.7, 146.1, 145.0, 136.7, 134.3, 129.2, 128.1, 126.1, 125.7, 123.6, 122.9, 122.1, 121.4, 117.2, 108.9, 35.0, 31.5. ESI‐MS: m/*z* [M+H]^+^ calcd for C_47_H_54_N_5_ [M+H]^+^ 688.4374 found 688.4349.

**2,6‐bis(6‐(3,5‐difluorophenyl)imidazo[1,2‐*a*]pyridin‐2‐yl)pyridine (5 f)**: pale brown solid, 0.667 g, yield 83%. ^1^H NMR (CDCl_3_, 500 MHz): δ 8.43 (s,2H), 8.35 (s, 2H), 8.15 (d, *J*=8 Hz, 2H), 7.91 (t, *J*=8 Hz, 1H), 7.74 (d, *J*=9.5 Hz, 2H), 7.40 (d, *J*=9.5 Hz, 2H), 7.11 (d, *J*=6 Hz, 4H), 6.85 (t, *J*=6 Hz, 2H). ^13^C NMR (DMSO‐d_6_, 126 MHz): δ 163.5 (dd, *J*_C‐F_ *=*13.6 Hz, *J*_C‐F_ *=*259.9 Hz), 152.8, 145.9, 144.7, 140.7 (t, *J*_C‐F_ *=*9.9 Hz), 138.4, 126.1, 125.4, 123.7, 119.6, 117.5, 112.4, 110.2 (dd, *J*_C‐F_ *=*6.4 Hz, *J*_C‐F_ *=*33.1 Hz), 103.6 (t, *J*_C‐F_ *=*25.8 Hz). ^19^F NMR (CDCl_3_, 300 MHz): δ −108.5. ESI‐MS: m/*z* [M+H]^+^ calcd for C_31_H_18_F_4_N_5_ [M+H]^+^ 536.1493 found 536.1478.

### Photophysical characterization

*Instrument details*. Absorption spectra were measured on a Varian Cary 100 double‐beam UV–VIS spectrophotometer and baseline corrected. Steady‐state emission spectra were recorded on a Horiba Jobin−Yvon IBH FL‐322 Fluorolog 3 spectrometer equipped with a 450 W xenon arc lamp, double‐grating excitation, and emission monochromators (2.1 nm mm^−1^ of dispersion; 1200 grooves mm^−1^) and a Hamamatsu R13456 red sensitive Peltier‐cooled PMT detector. Emission and excitation spectra were corrected for source intensity (lamp and grating) and emission spectral response (detector and grating) by standard correction curves. Time‐resolved measurements were performed using the time‐correlated single‐photon counting (TCSPC) electronics option of the TimeHarp 260 board installed on a PicoQuant FluoTime 300 fluorimeter (PicoQuant GmbH, Germany), equipped with a PDL 820 laser pulse driver. A pulsed laser diode LDH‐P‐C‐375 (λ=375 nm, pulse full width at half maximum FWHM <50 ps, repetition rate 200 kHz–40 MHz) was used to excite the sample and mounted directly on the sample chamber at 90**°**. The photons were collected by a PMA Hybrid‐07 single photon counting detector. The data were acquired by using the commercially available software EasyTau II (PicoQuant GmbH, Germany), while data analysis was performed using the built‐in software FluoFit (PicoQuant GmbH, Germany).

*Methods*. For time resolved measurements, data fitting was performed by employing the maximum likelihood estimation (MLE) methods and the quality of the fit was assessed by inspection of the reduced χ^2^ function and of the weighted residuals. For multi‐exponential decays, the intensity, namely I*(t)*, has been assumed to decay as the sum of individual single exponential decays (Eqn. 1:

(1) $$I\left(t\right)=\sum_{i=1}^{n}\alpha_{i}exp\left(-\frac{t}{\tau_{i}}\right)$$

where *τ_i_* are the decay times and *α_i_* are the amplitude of the component at *t*=0. In the tables, the percentages to the pre‐exponential factors, *α_i_*, are listed upon normalization. Luminescence quantum yields were measured in optically dilute solutions (optical density <0.1 at the excitation wavelength). The absolute photoluminescence quantum yields (PLQY) were measured on a Hamamatsu Quantaurus‐QY C11347‐11 integrating sphere in air‐equilibrated condition using an empty quartz tube as the reference upon excitation at λ_exc_=300–330 nm.

### X‐ray diffractometric analysis

The crystals were placed in oil, and a single crystal was selected, mounted on a glass fibre and placed in a low‐temperature N_2_ stream. X‐ray diffraction data collection was carried out on a Bruker PHOTON III DUO CPAD diffractometer equipped with an Oxford Cryosystem liquid N_2_ device, using Mo−Kα radiation (λ=0.71073 Å). The crystal‐detector distance was 38 mm. The cell parameters were determined (APEX3 software)^[29]^ from reflections taken from 1 set of 180 frames at one second exposure. The structure was solved using the program SHELXT‐2018.^[30]^ The refinement and all further calculations were carried out using SHELXL‐2018.^[31]^ The H‐atoms were included in calculated positions and treated as riding atoms using SHELXL default parameters. The non‐H atoms were refined anisotropically, using weighted full‐matrix least‐squares on *F* ^2^. A semi‐empirical absorption correction was applied using SADABS in APEX3;^[29]^ transmission factors: T_min/_T_max_=0.6357/0.7456. CCDC reference numbers CCDC 2183608 and 2183609 for compound **5 a** and **4 c** **⋅** **CH_2_Cl_2_**, respectively.

### Computational details

A first set of calculations have been performed with GAUSSIAN 09 version D01 at DFT level of theory with B3LYP functional.^[32]^ All atoms were described by the 6‐31+G** basis set.^[33]^ Van der Waals forces were introduced through Grimme's corrections.^[34]^ Solvent (CH_2_Cl_2_) was introduced through PCM model.^[35]^ All geometries were fully optimized and the nature of the encountered stationary point determined by frequency analysis. Minima were characterized by a full set of real frequencies and transition states by one imaginary frequency. Gibbs Free energies were extracted from the frequency analysis. A second set of calculations have been performed with ADF 2019 at DFT level of theory with B3LYP functional. All atoms were described by the TZP basis set.^[36]^ Van der Waals forces were introduced through Grimme's corrections. Solvent (CH_2_Cl_2_) was introduced through COSMO model.^[37]^ Scalar relativistic ZORA Hamiltonian was employed. All geometries were fully optimized and the absorption spectra computed on the computed stationary point by mean of TD‐DFT.^[38]^ Excited states structures were optimized the same way. Tamm‐Dancoff approximation were used to avoid triplet instability.^[39]^ The nature of the electronic transition was determined by THEODore analysis of the ADF TD‐DFT results.^[40]^

## Conflict of interest

The authors declare no conflict of interest.

1

## Supporting information

As a service to our authors and readers, this journal provides supporting information supplied by the authors. Such materials are peer reviewed and may be re‐organized for online delivery, but are not copy‐edited or typeset. Technical support issues arising from supporting information (other than missing files) should be addressed to the authors.

Supporting Information

## Data Availability

The data that support the findings of this study are available from the corresponding author upon reasonable request.
