# Supplementary material for: Bright V‐Shaped bis‐Imidazo[1,2‐a]pyridine Fluorophores with Near‐UV to Deep‐Blue Emission
Source: Chem Asian J. 2022 Oct 26;17(23):e202200903. doi: 10.1002/asia.202200903 (PMC10091824; doi:10.1002/asia.202200903)
Supplement: Supplementary file 1 — Supporting Information [file ASIA-17-0-s001.pdf]

CHEMISTRY
AN ASIAN JOURNAL

Supporting Information

Bright V-Shaped *bis*-Imidazo[1,2-*a*]pyridine Fluorophores with Near-UV to Deep-Blue Emission

Elise Jouaiti, Valerio Giuso, Damien Cianfarani, Nathalie Kyritsakas, Christophe Gourlaouen, and Matteo Mauro*© 2022 The Authors. Chemistry - An Asian Journal published by Wiley-VCH GmbH. This is an open access article under the terms of the Creative Commons Attribution License, which permits use, distribution and reproduction in any medium, provided the original work is properly cited.

Table of contents

	Page
1. ^{1}H and ^{13}C NMR spectra	S13–S21
2. Supplementary crystallographic figures	S22
3. Supplementary computational figures	S23–S24
4. Supplementary tables	S25–S33

1. NMR spectra

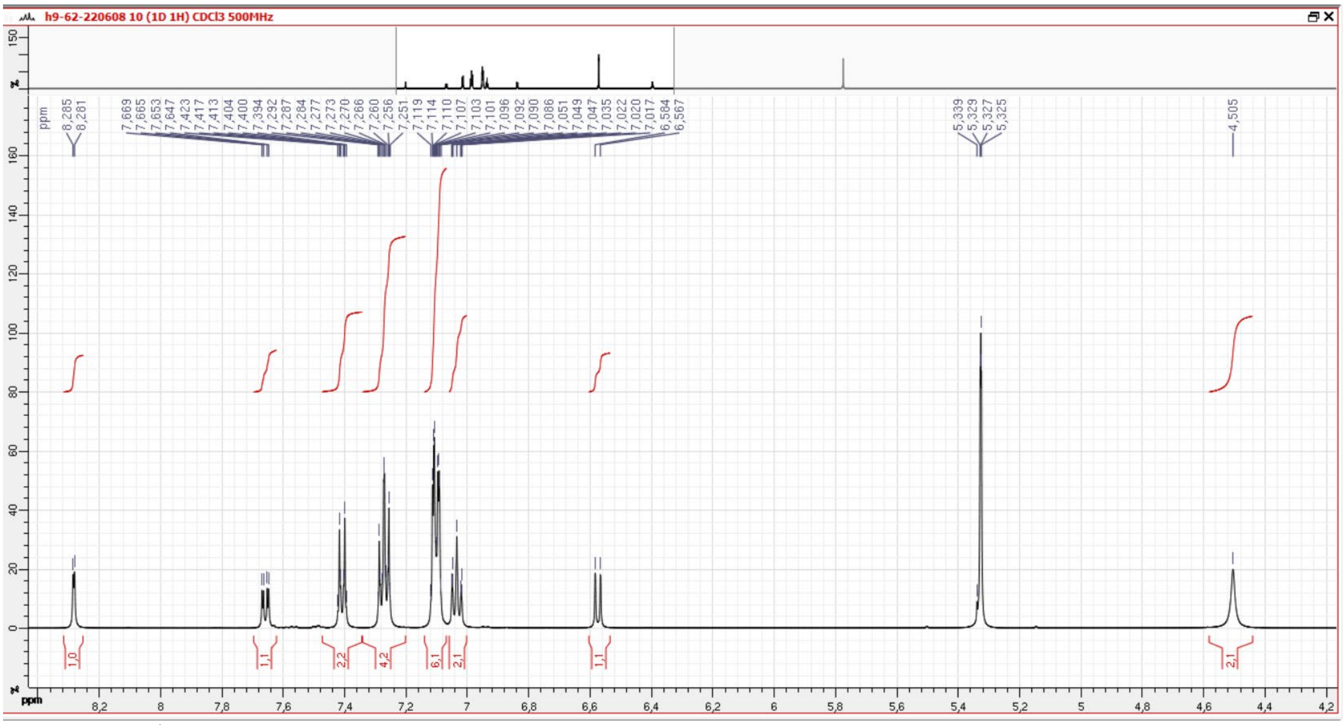

Figure S1. ^{1}H NMR (500 MHz, 298K) spectrum of compound **3c** in $CDCl_3$.

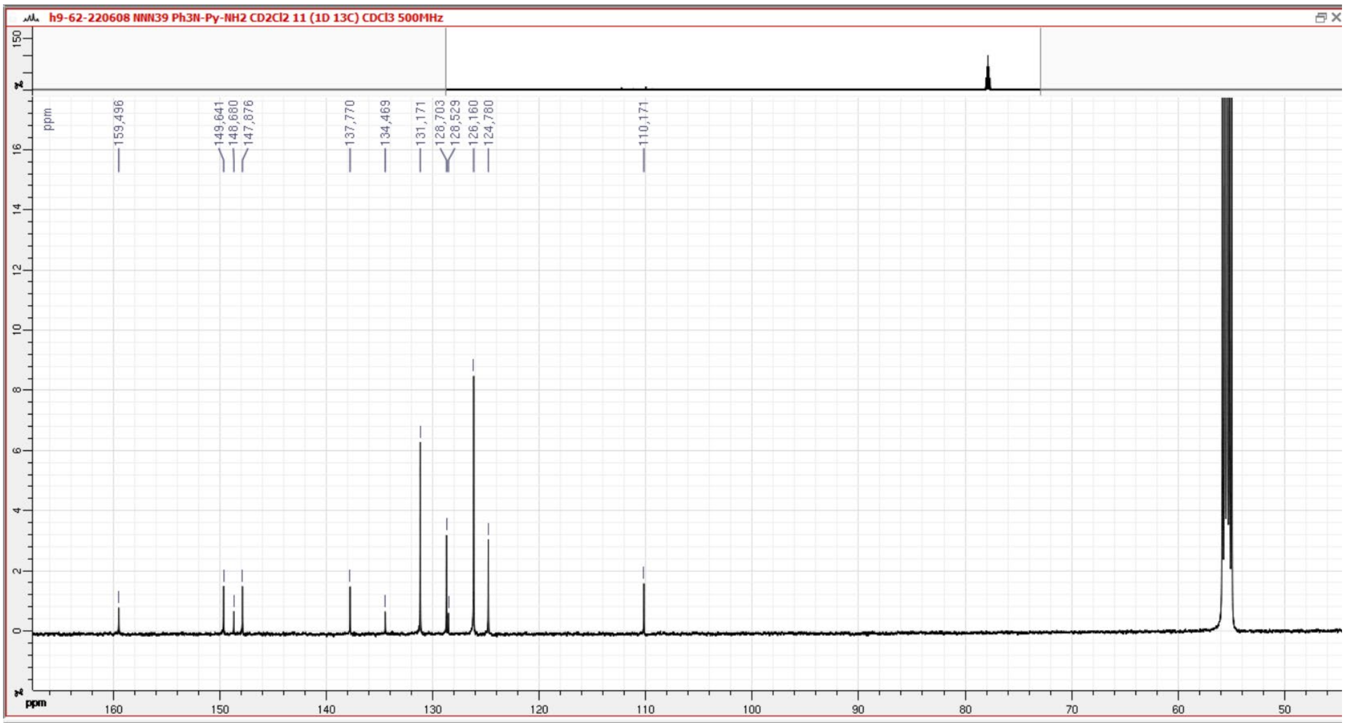

Figure S2. ^{13}C NMR (125 MHz, 298K) spectrum of compound **3c** in $CDCl_3$.

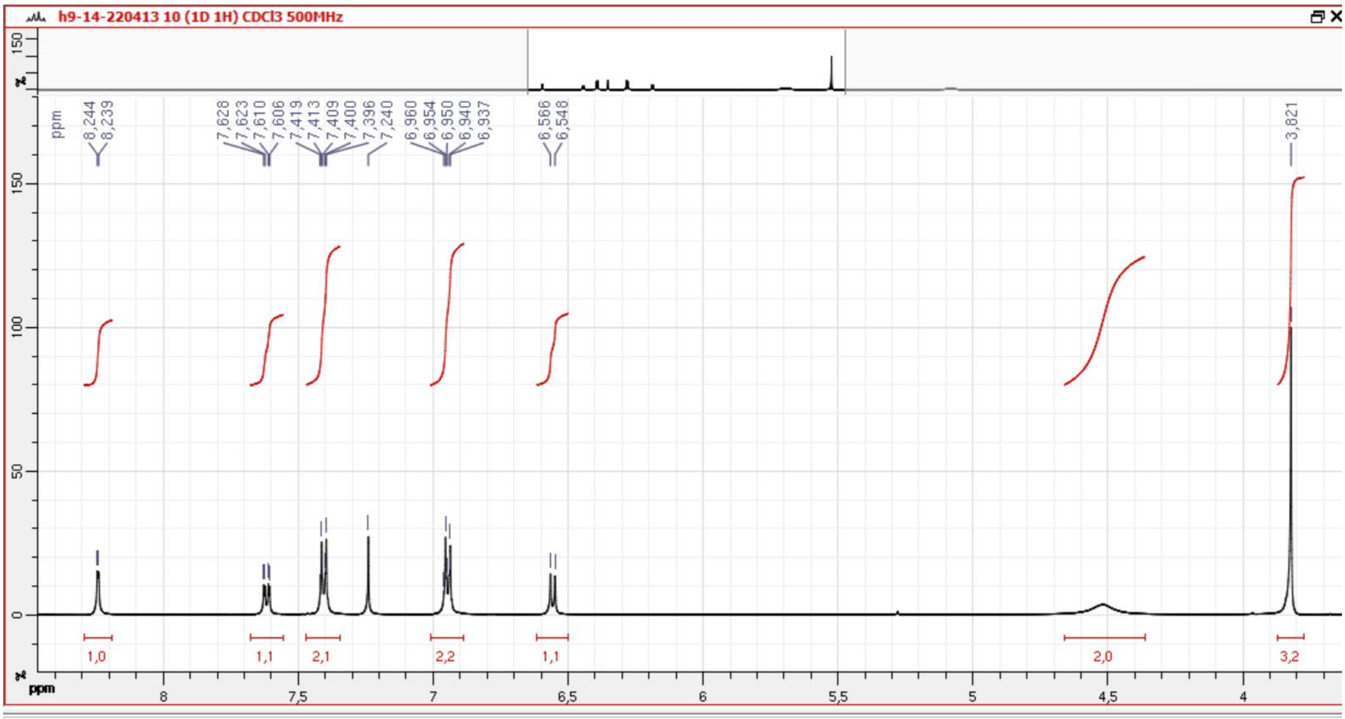

Figure S3. ^{1}H NMR (500 MHz, 298K) spectrum of compound **3d** in $CDCl_3$.

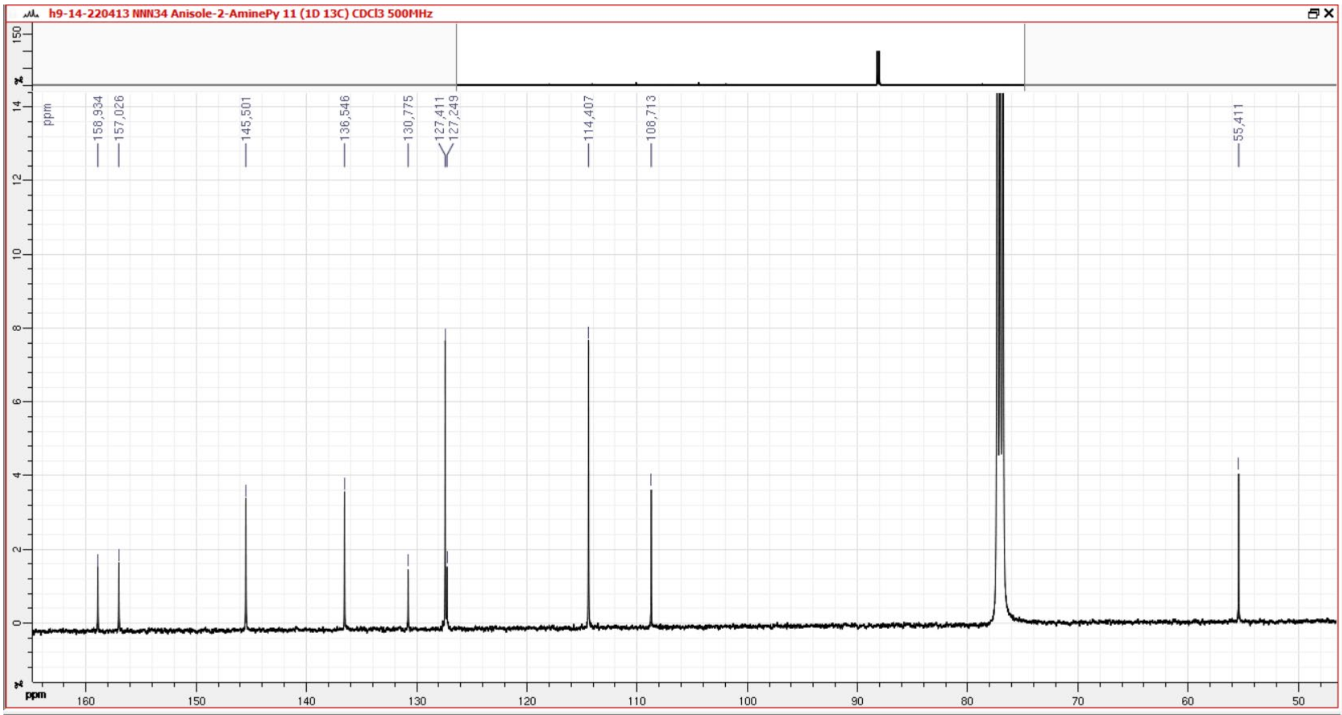

Figure S4. ^{13}C NMR (125 MHz, 298K) spectrum of compound **3d** in $CDCl_3$.

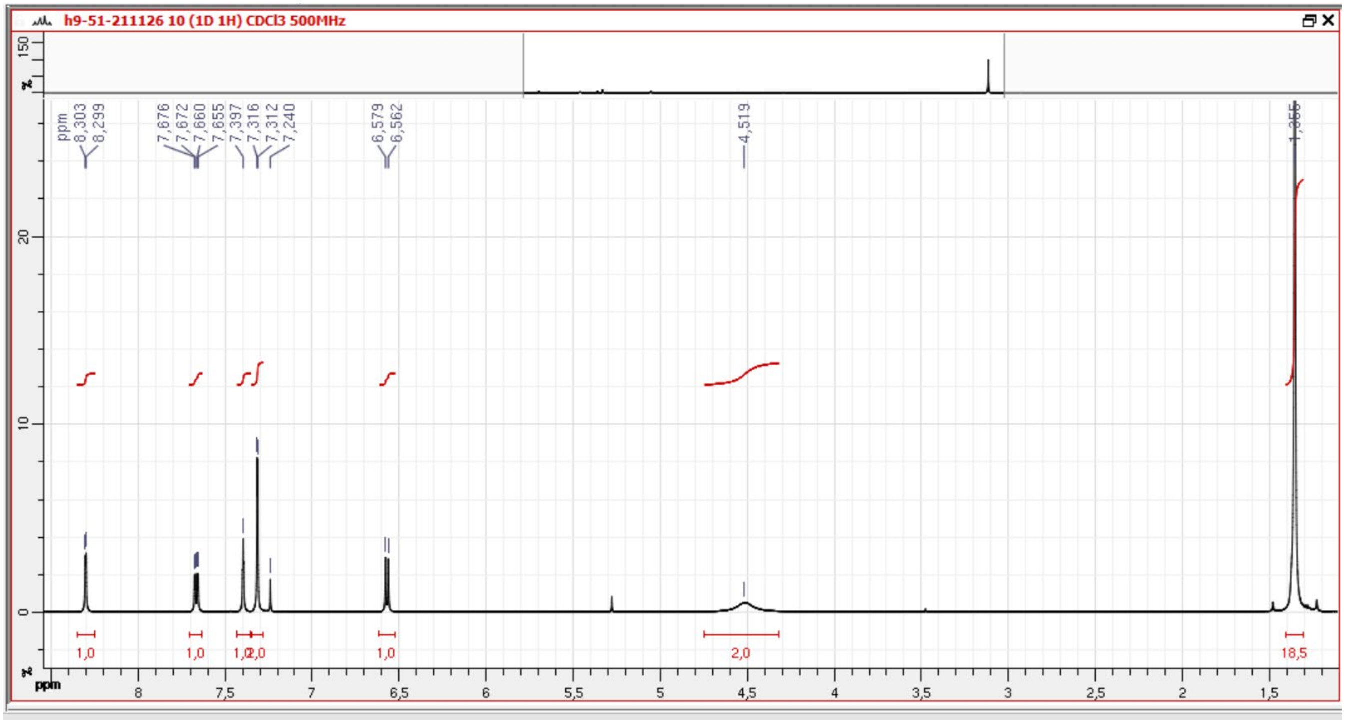

Figure S5. ^{1}H NMR (500 MHz, 298K) spectrum of compound **3e** in $CDCl_3$.

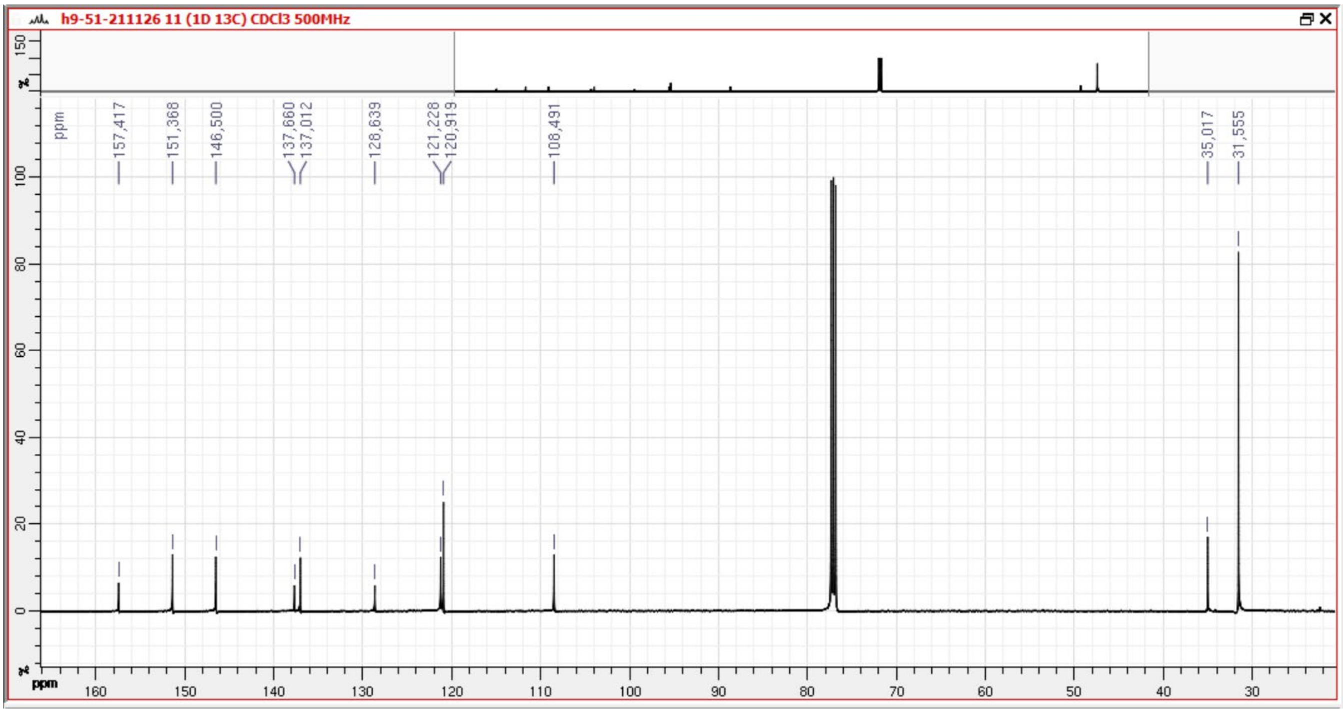

Figure S6. ^{13}C NMR (125 MHz, 298K) spectrum of compound **3e** in $CDCl_3$.

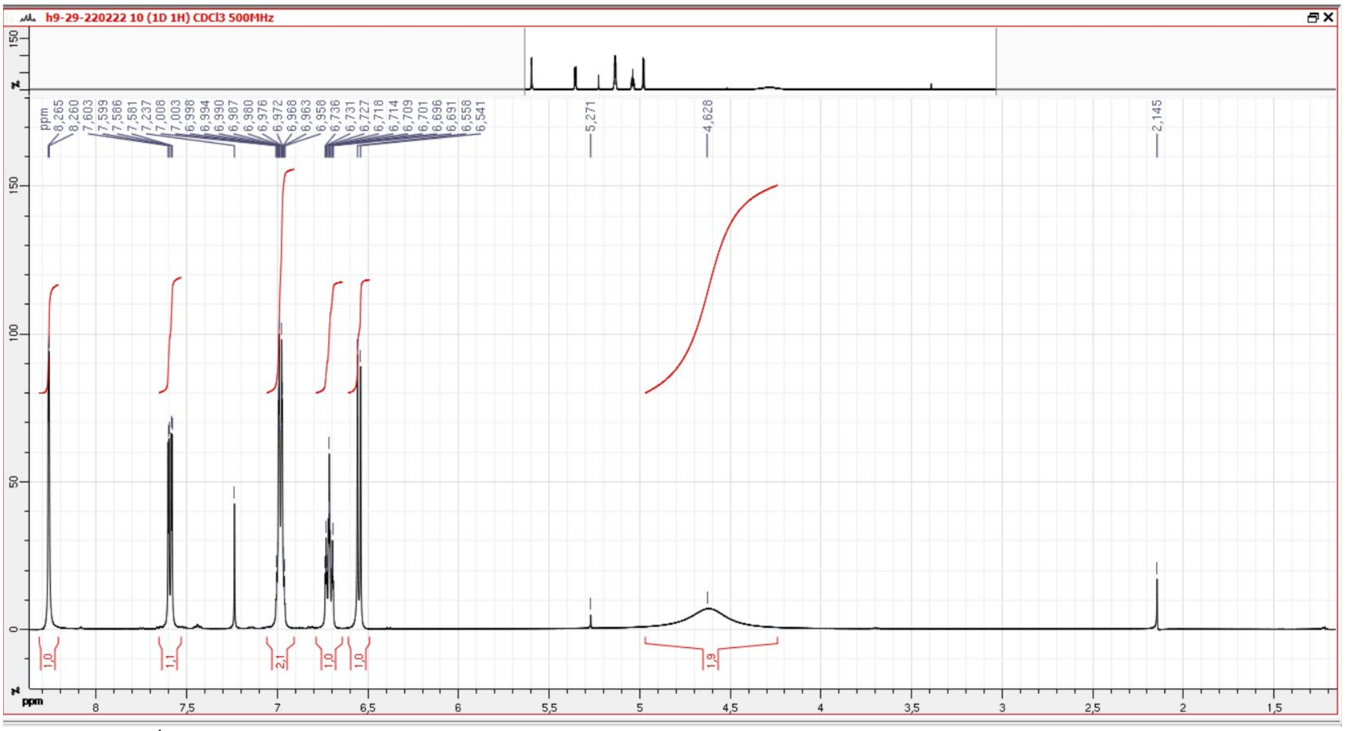

Figure S7. ^{1}H NMR (500 MHz, 298K) spectrum of compound **3f** in $CDCl_3$.

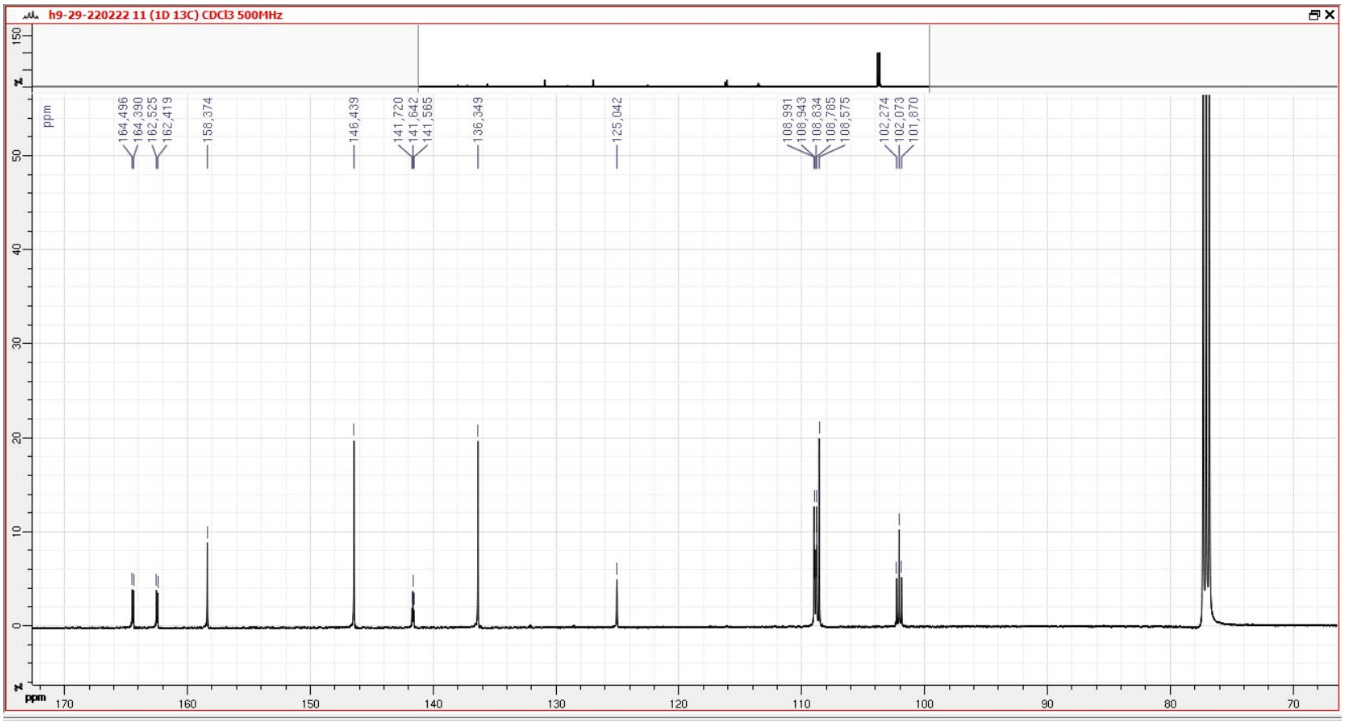

Figure S8. ^{13}C NMR (125 MHz, 298K) spectrum of compound **3f** in $CDCl_3$.

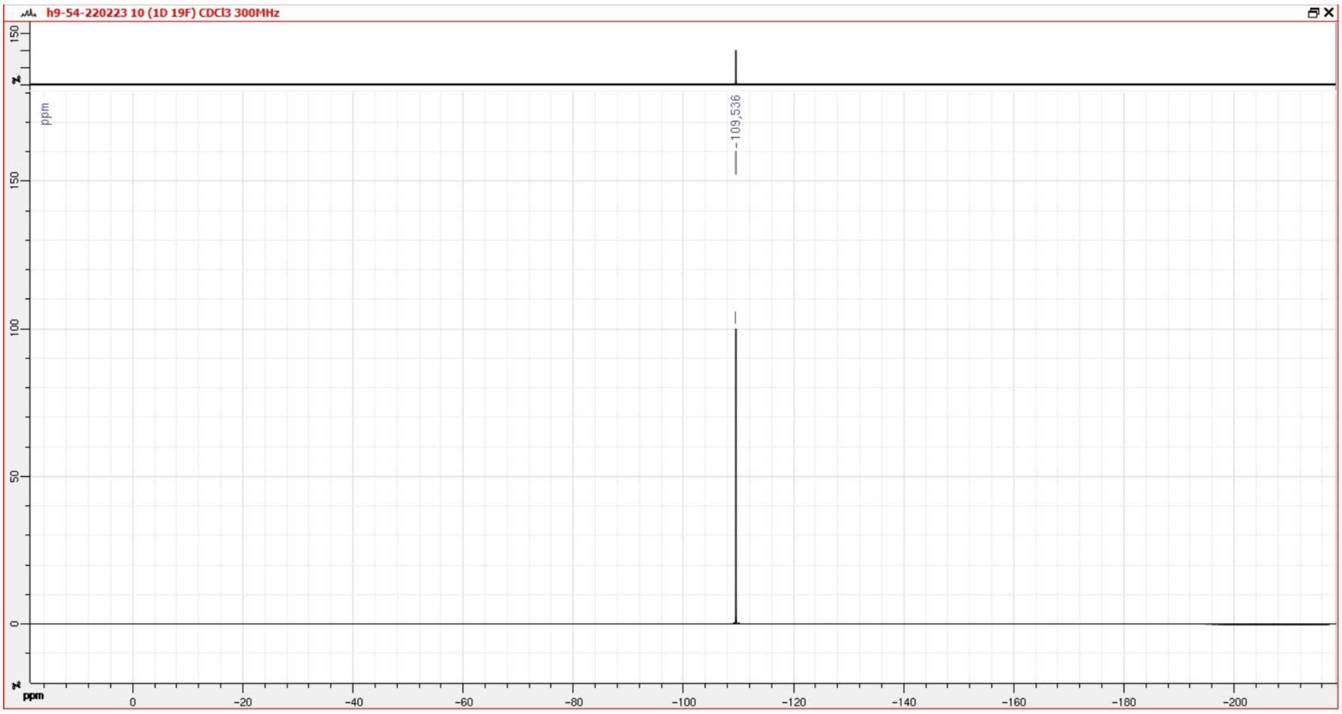

Figure S9. ^{19}F NMR (282 MHz, 298K) spectrum of compound **3f** in $CDCl_3$.

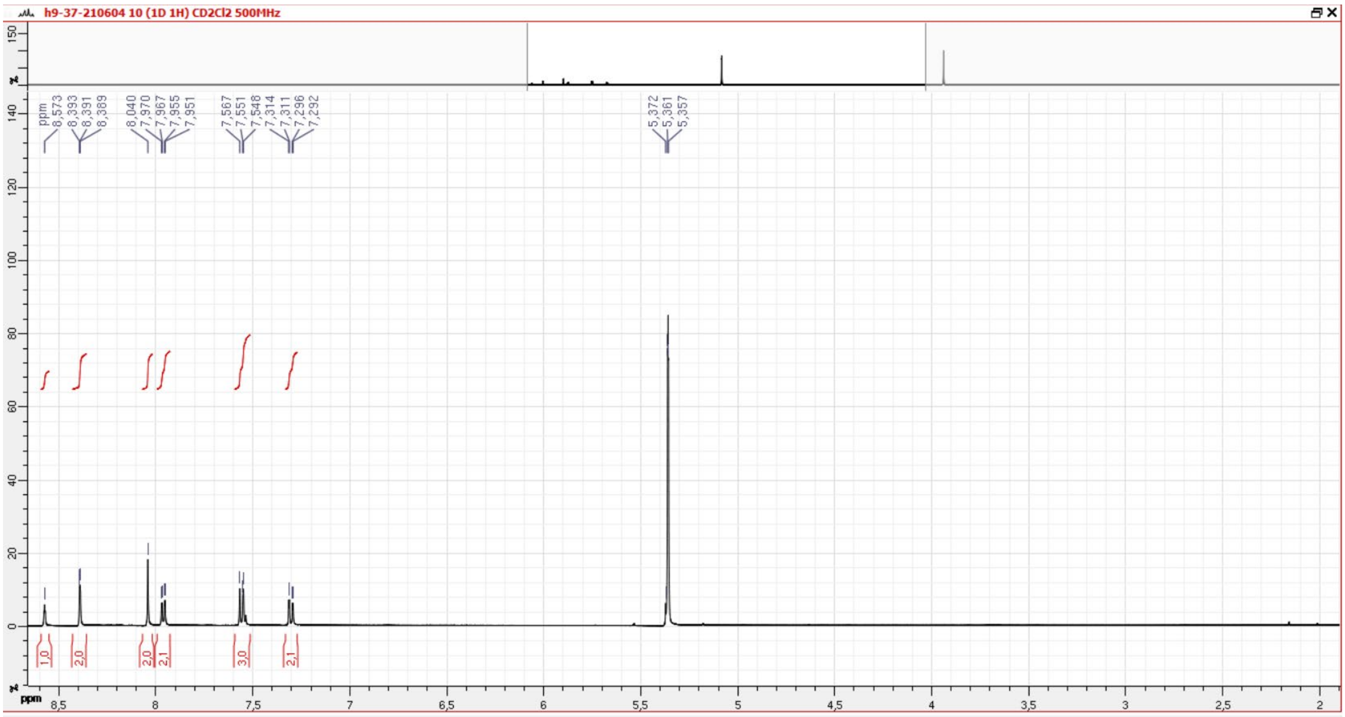

Figure S10. ^{1}H NMR (500 MHz, 298K) spectrum of compound **4a** in CD_2Cl_2.

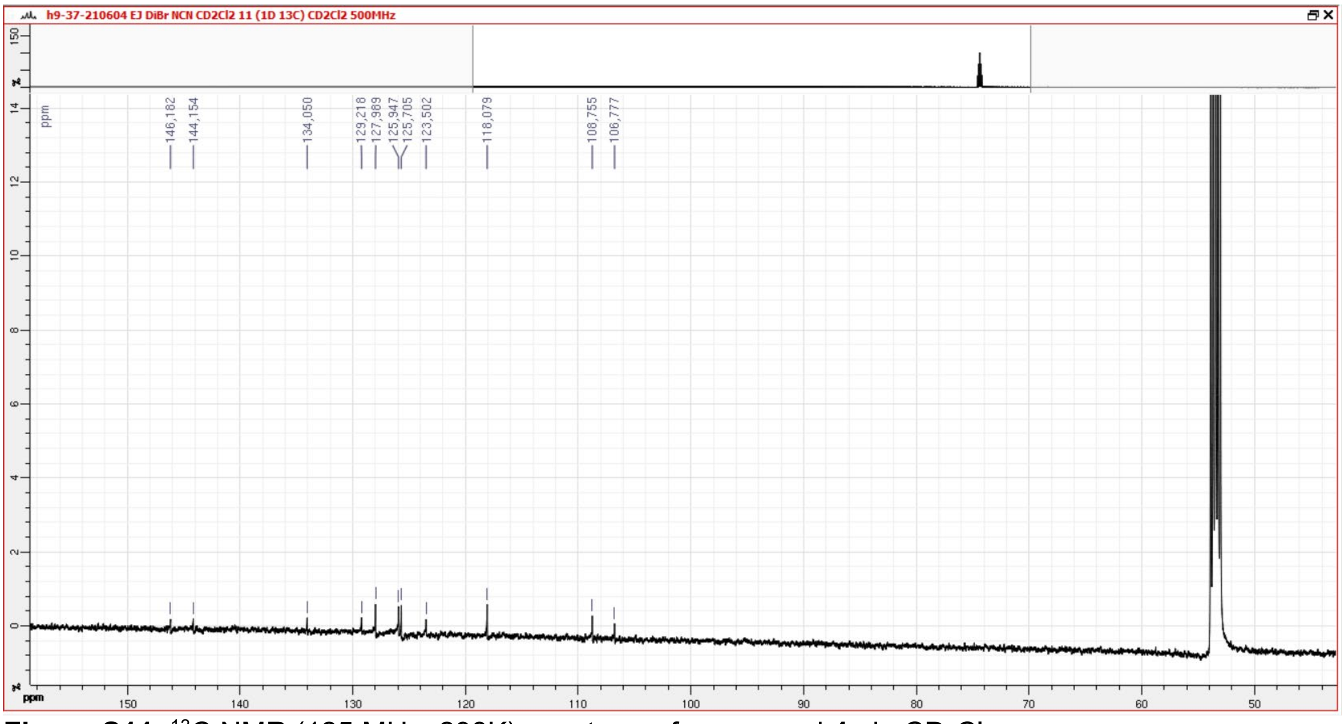

Figure S11. ^{13}C NMR (125 MHz, 298K) spectrum of compound **4a** in CD_2Cl_2.

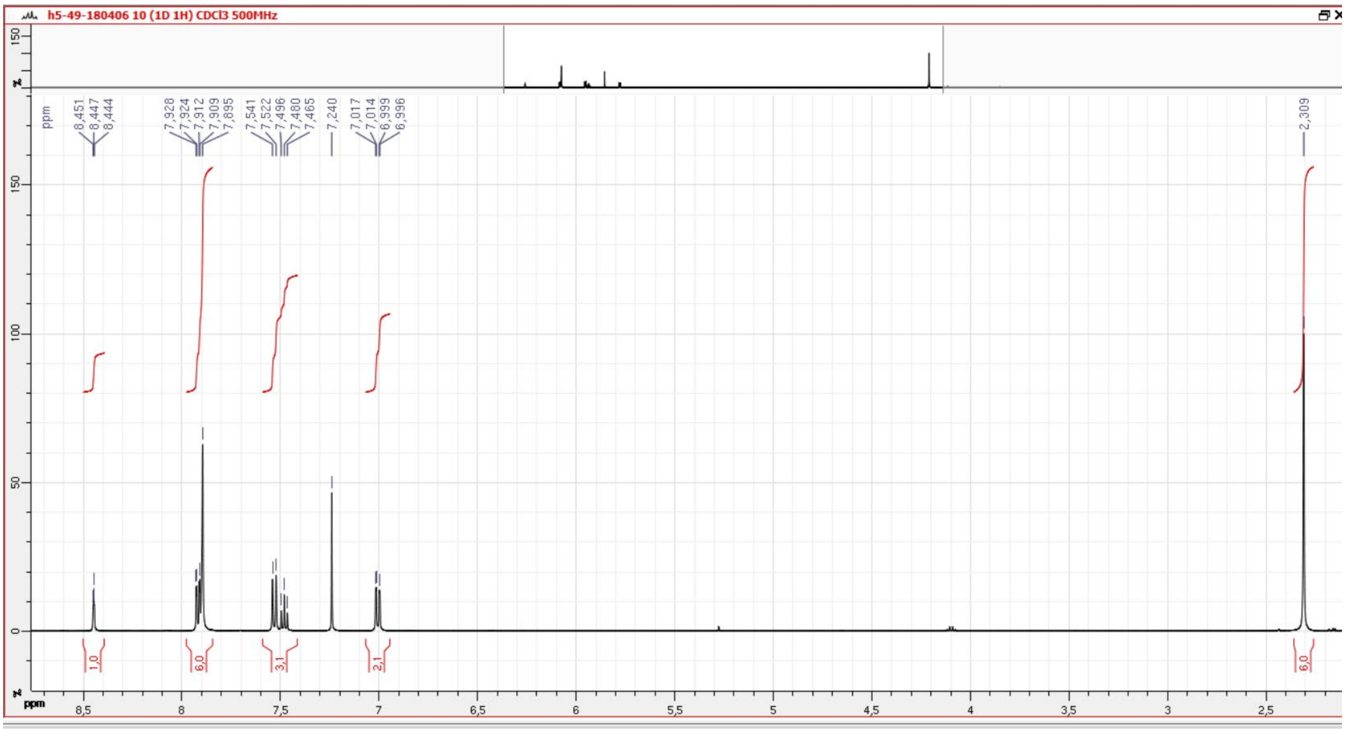

Figure S12. ^{1}H NMR (500 MHz, 298K) spectrum of compound **4b** in $CDCl_3$.

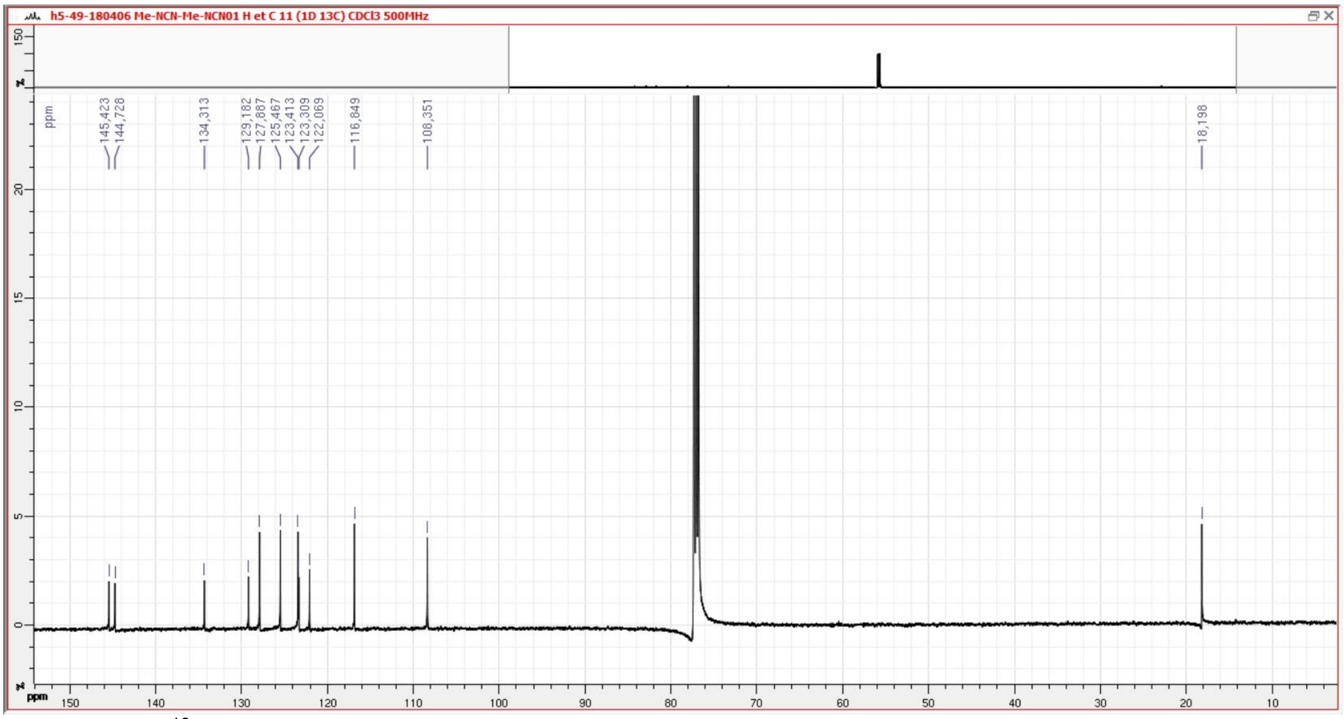

Figure S13. ^{13}C NMR (125 MHz, 298K) spectrum of compound **4b** in $CDCl_3$.

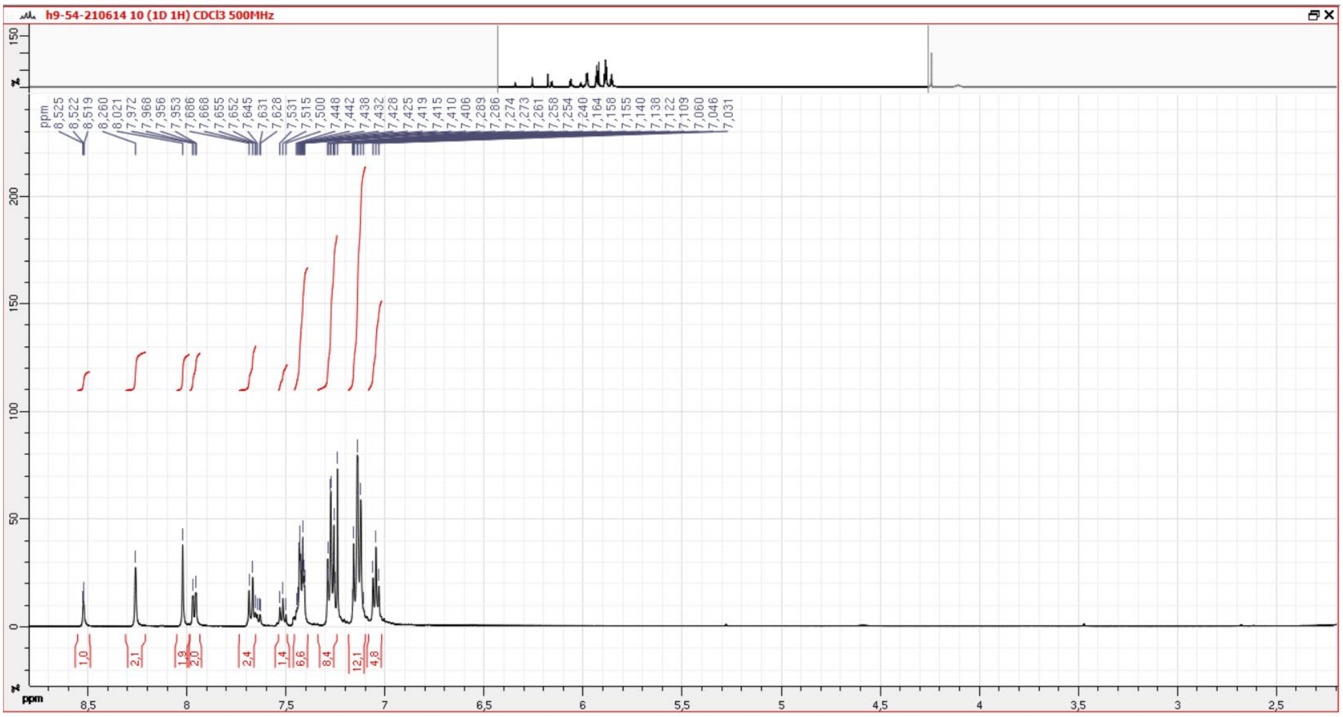

Figure S14. ^{1}H NMR (500 MHz, 298K) spectrum of compound **4c** in $CDCl_3$.

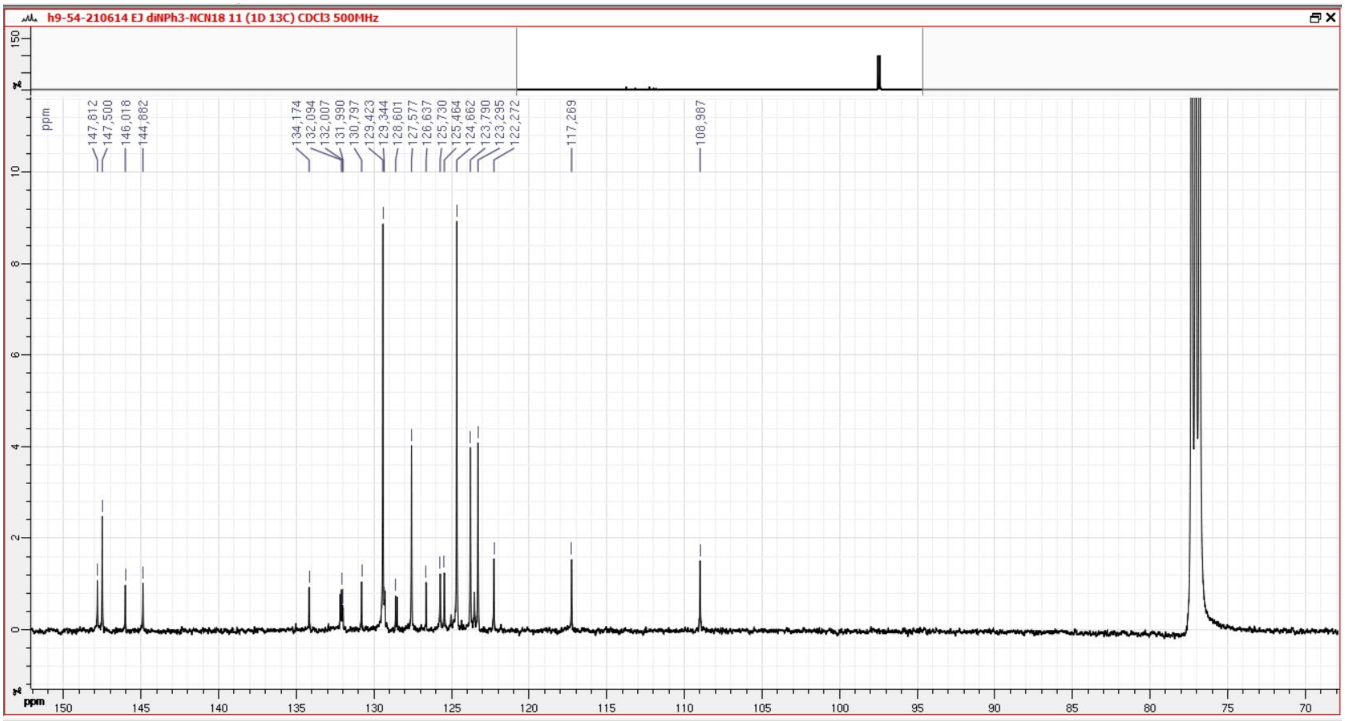

Figure S15. ^{13}C NMR (125 MHz, 298K) spectrum of compound **4c** in $CDCl_3$.

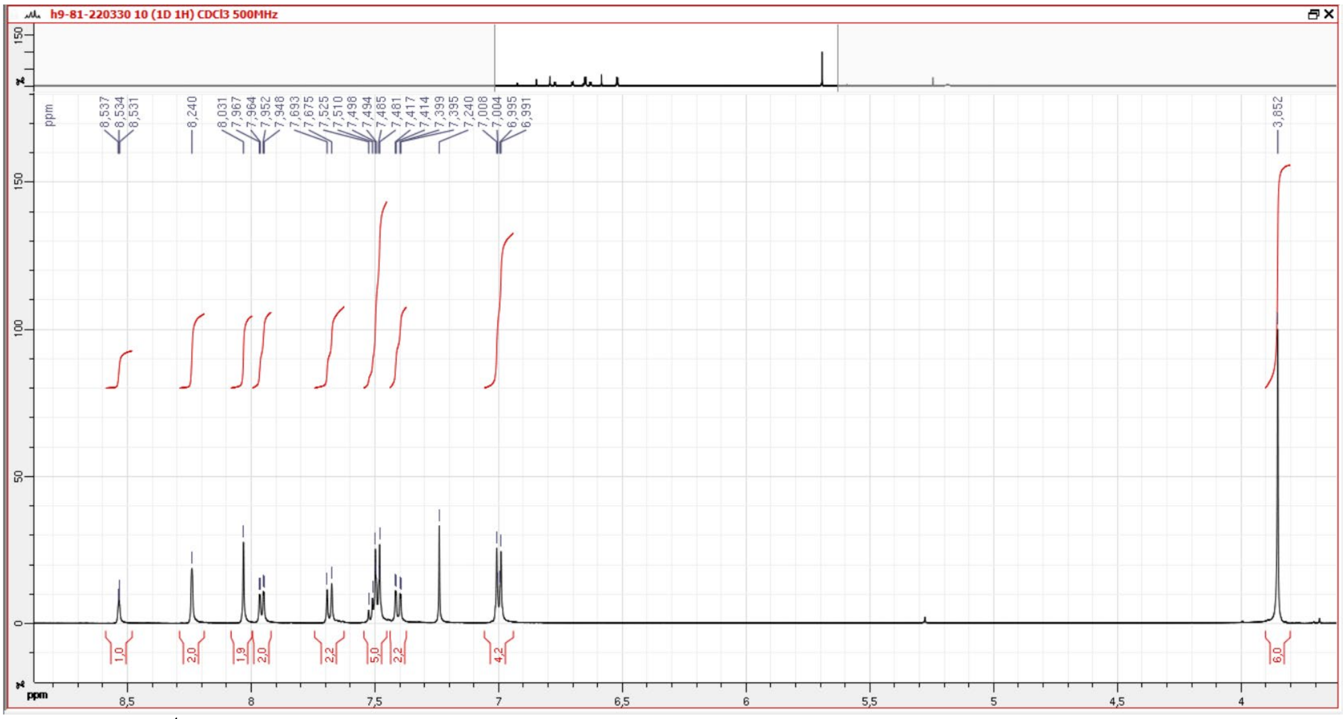

Figure S16. ^{1}H NMR (500 MHz, 298K) spectrum of compound **4d** in $CDCl_3$.

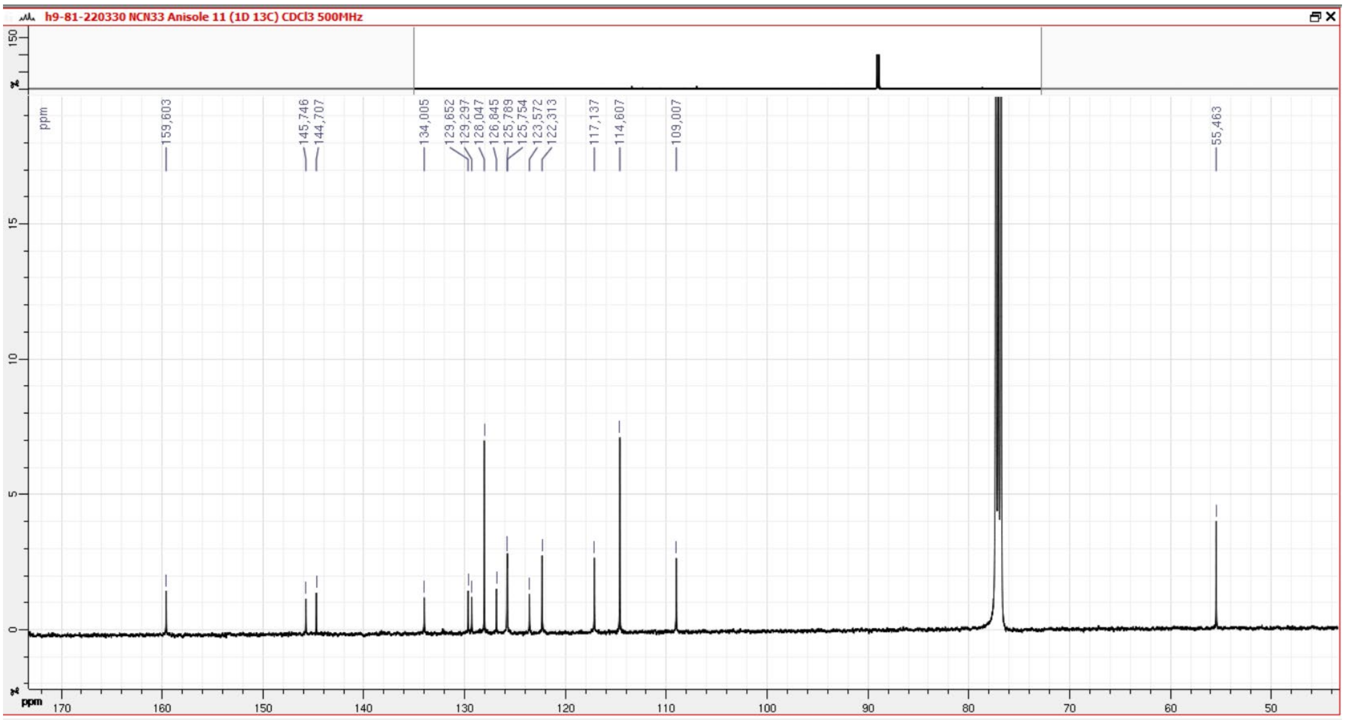

Figure S17. ^{13}C NMR (125 MHz, 298K) spectrum of compound **4d** in $CDCl_3$.

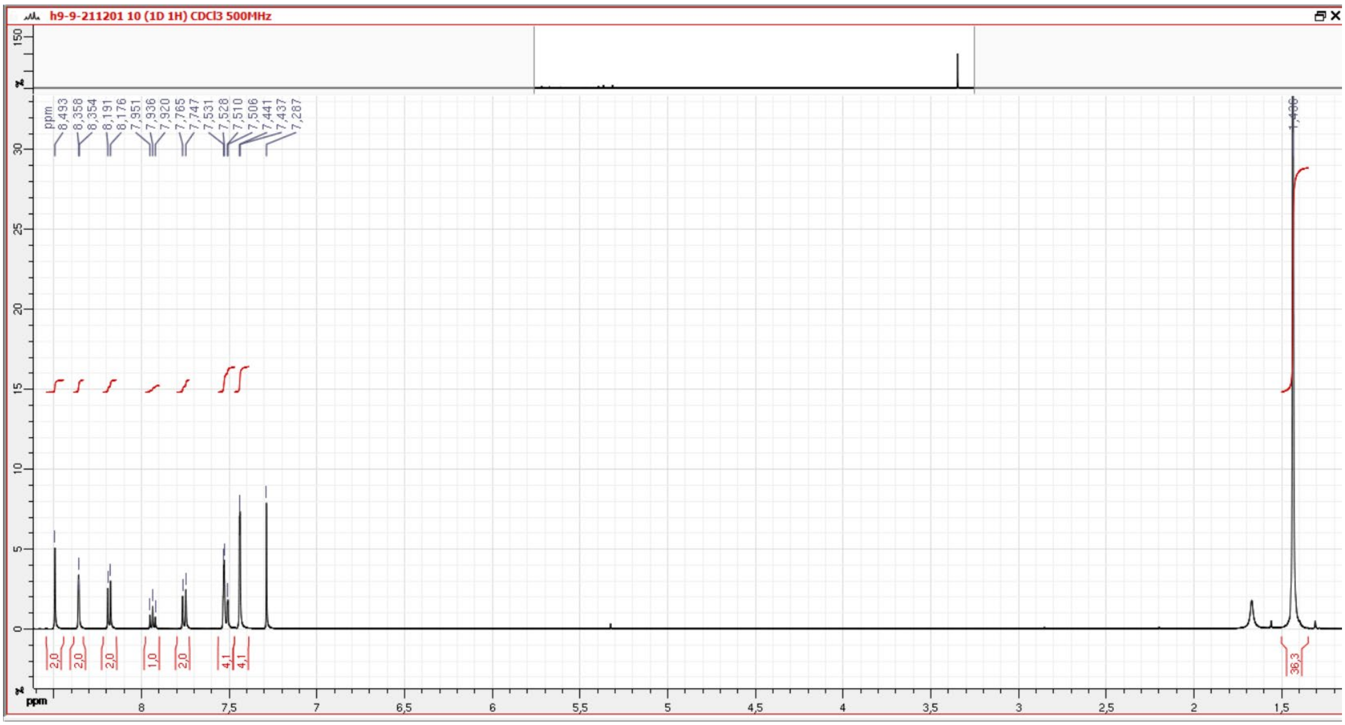

Figure S18. ^{1}H NMR (500 MHz, 298K) spectrum of compound **4e** in $CDCl_3$.

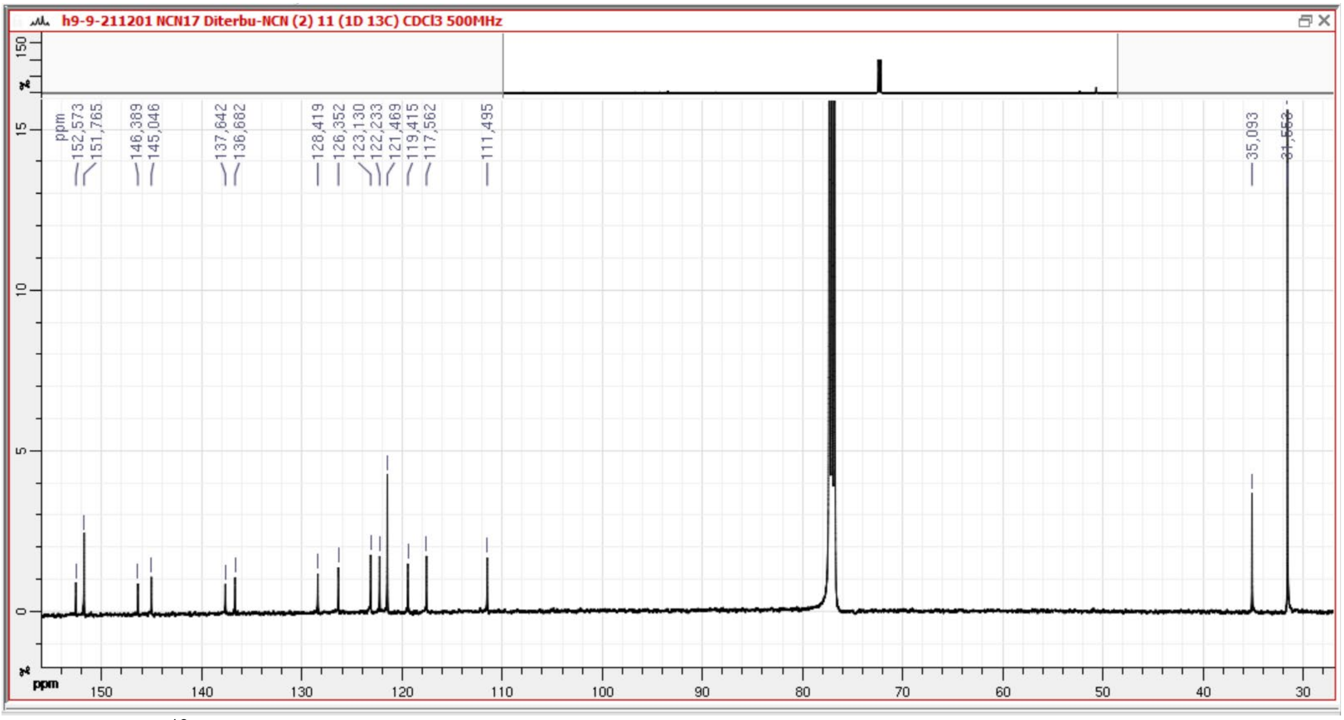

Figure S19. ^{13}C NMR (125 MHz, 298K) spectrum of compound **4e** in $CDCl_3$.

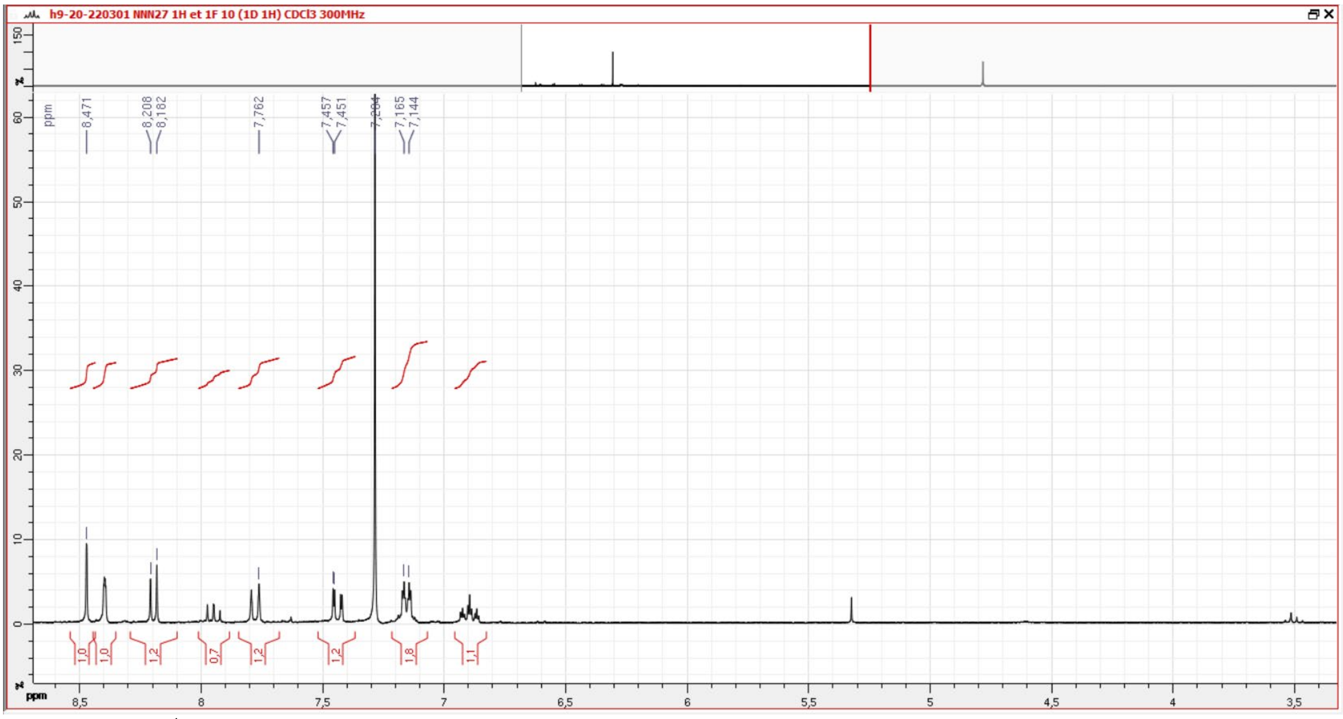

Figure S20. ^{1}H NMR (500 MHz, 298K) spectrum of compound **4f** in $CDCl_3$.

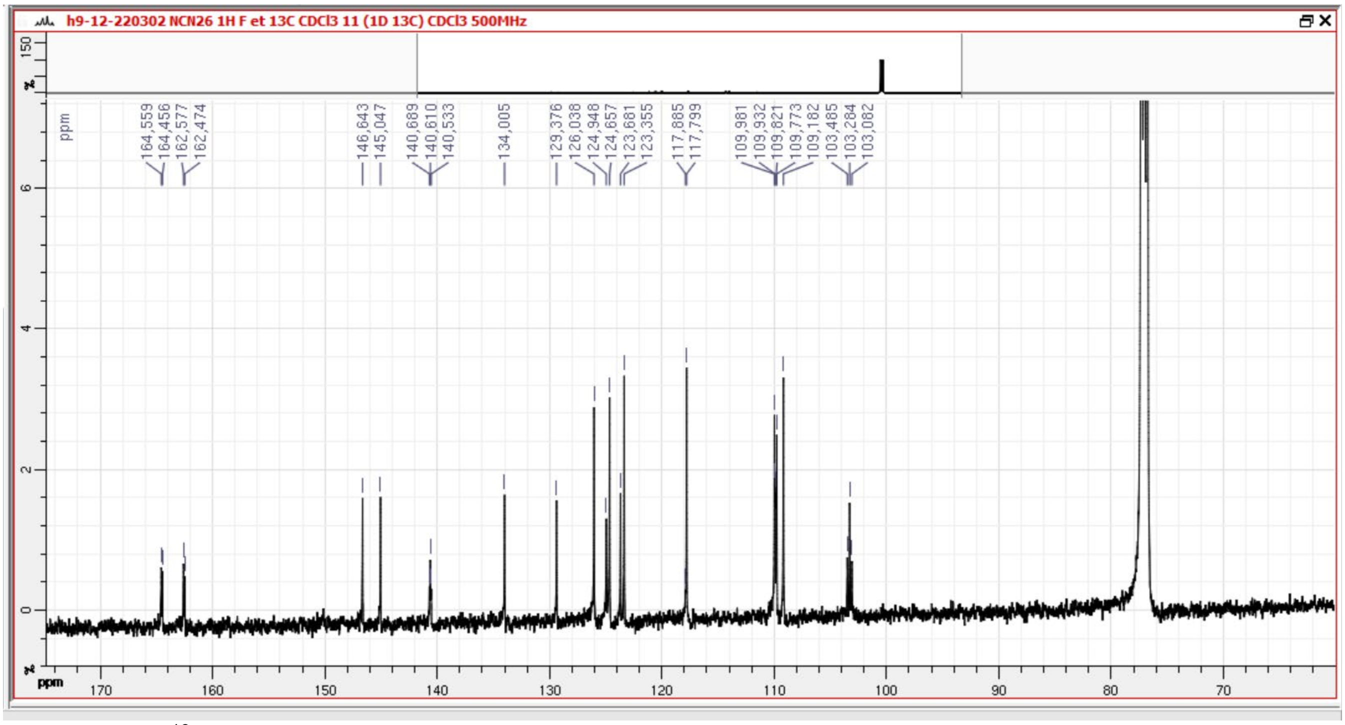

Figure S21. ^{13}C NMR (125 MHz, 298K) spectrum of compound **4f** in $CDCl_3$.

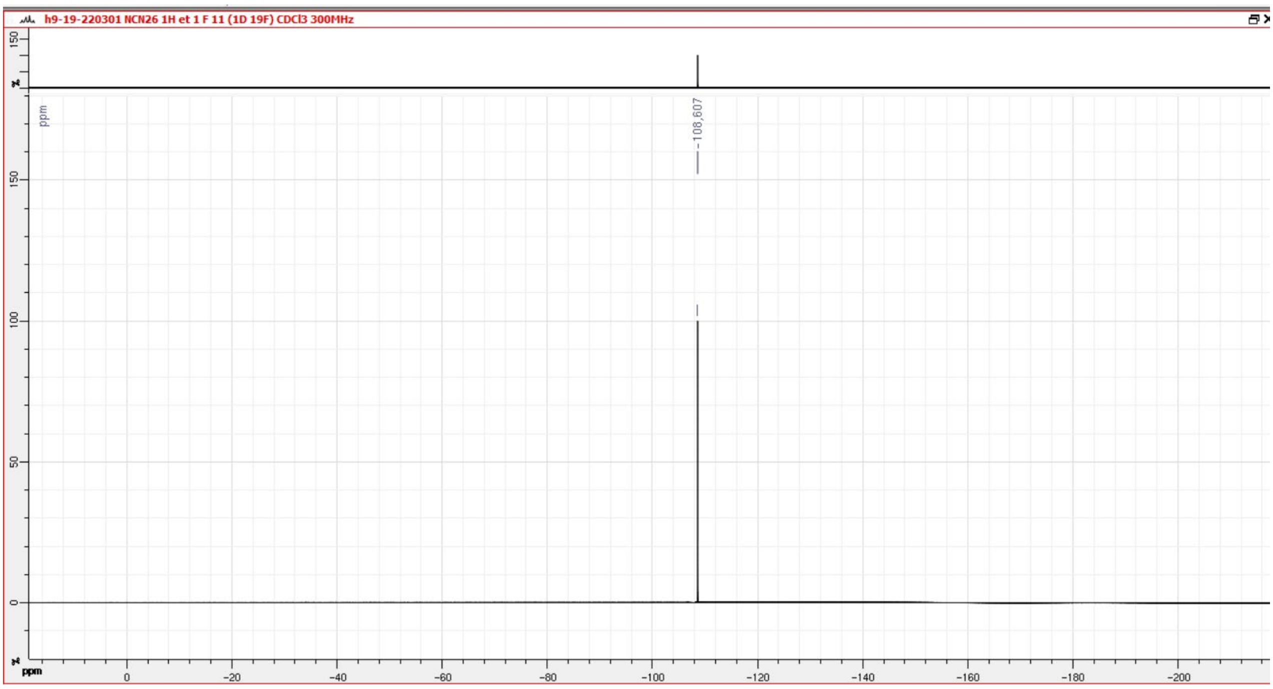

Figure S22. ^{19}F NMR (282 MHz, 298K) spectrum of compound **4f** in $CDCl_3$.

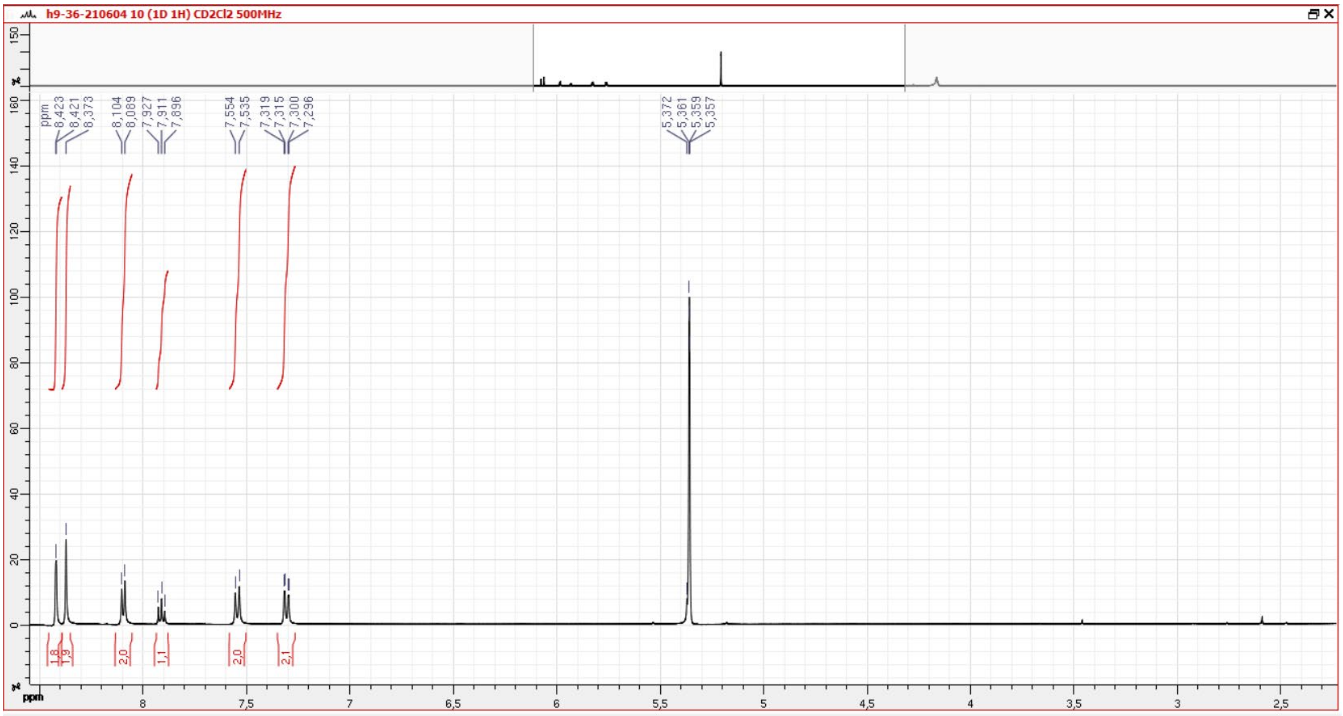

Figure S23. ^{1}H NMR (500 MHz, 298K) spectrum of compound **5a** in CD_2Cl_2.

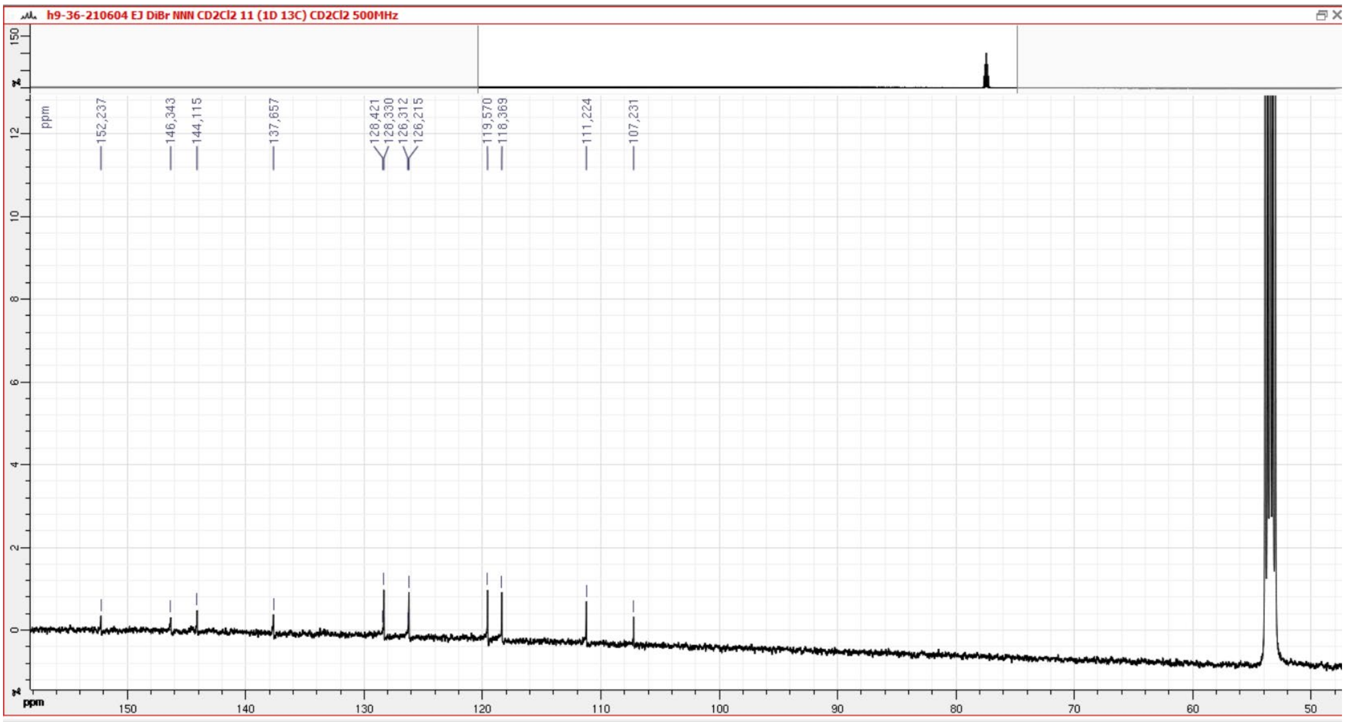

Figure S24. ^{13}C NMR (125 MHz, 298K) spectrum of compound **5a** in CD_2Cl_2.

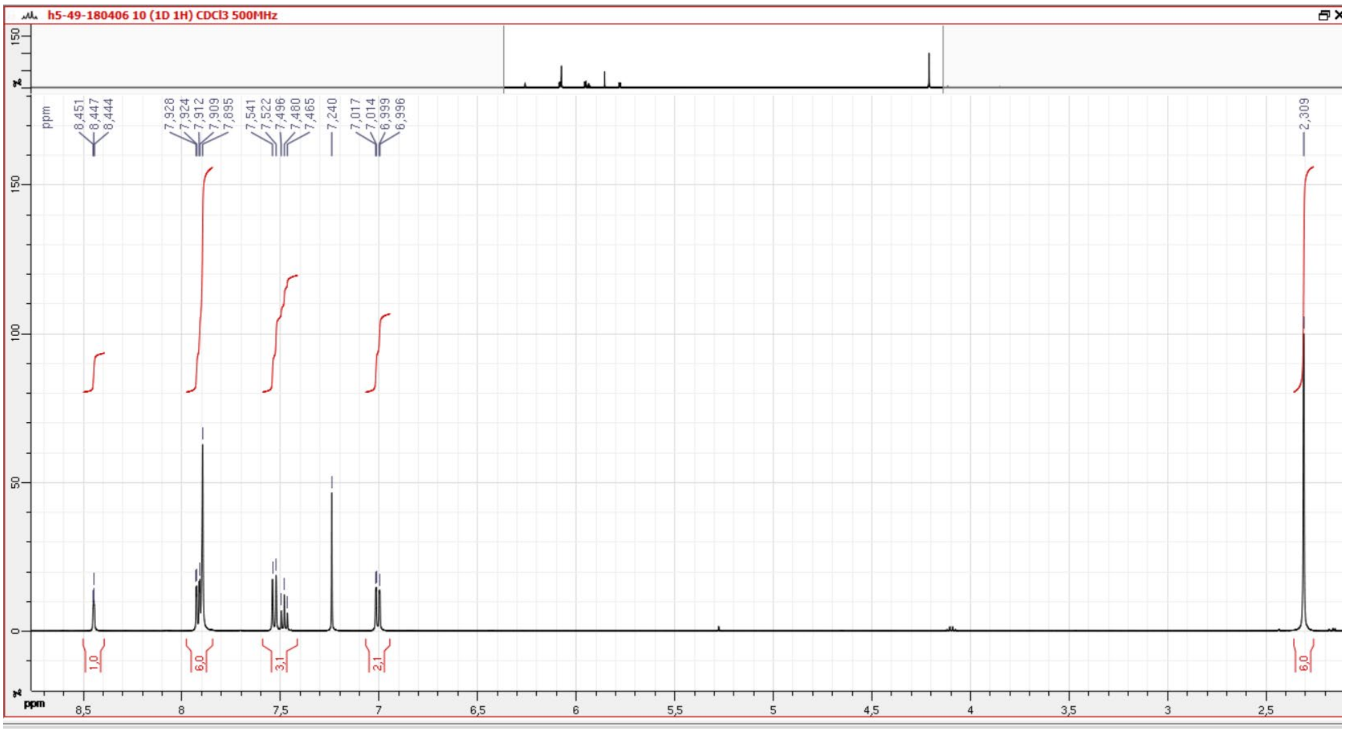

Figure S25. ^{1}H NMR (500 MHz, 298K) spectrum of compound **5b** in $CDCl_3$.

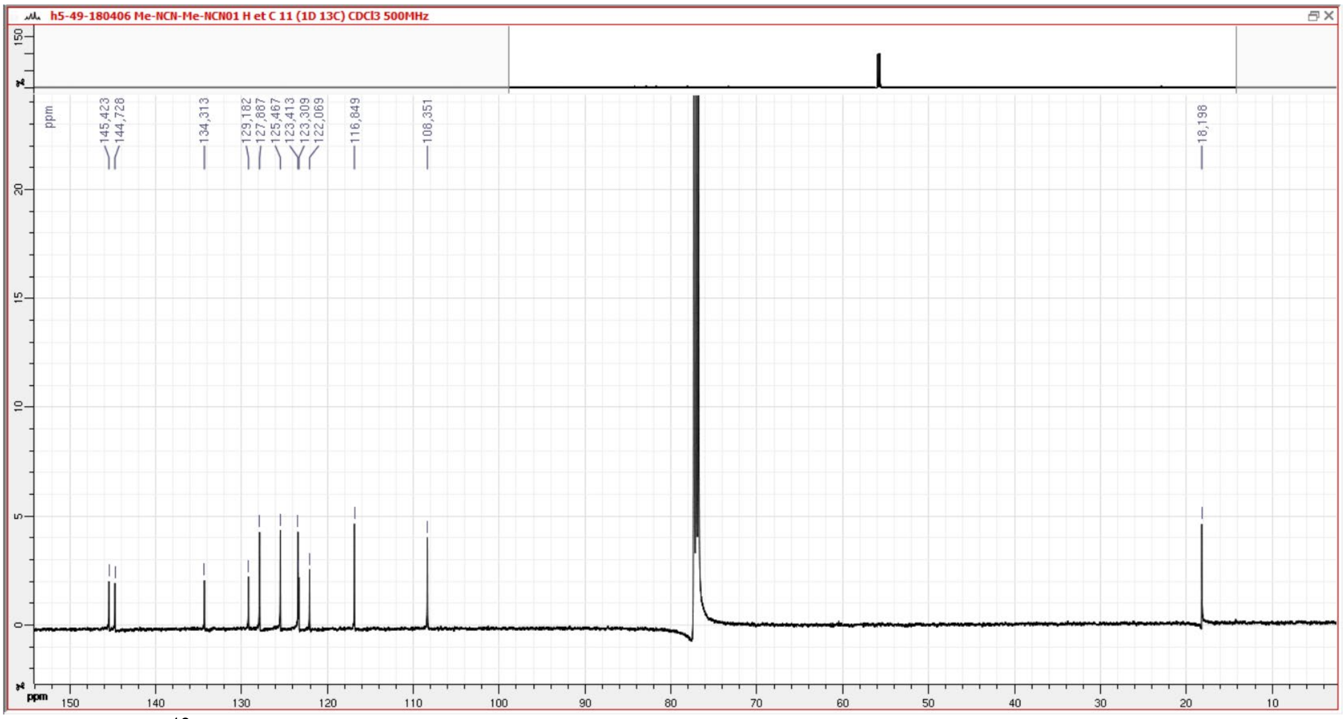

Figure S26. ^{13}C NMR (125 MHz, 298K) spectrum of compound **5b** in $CDCl_3$.

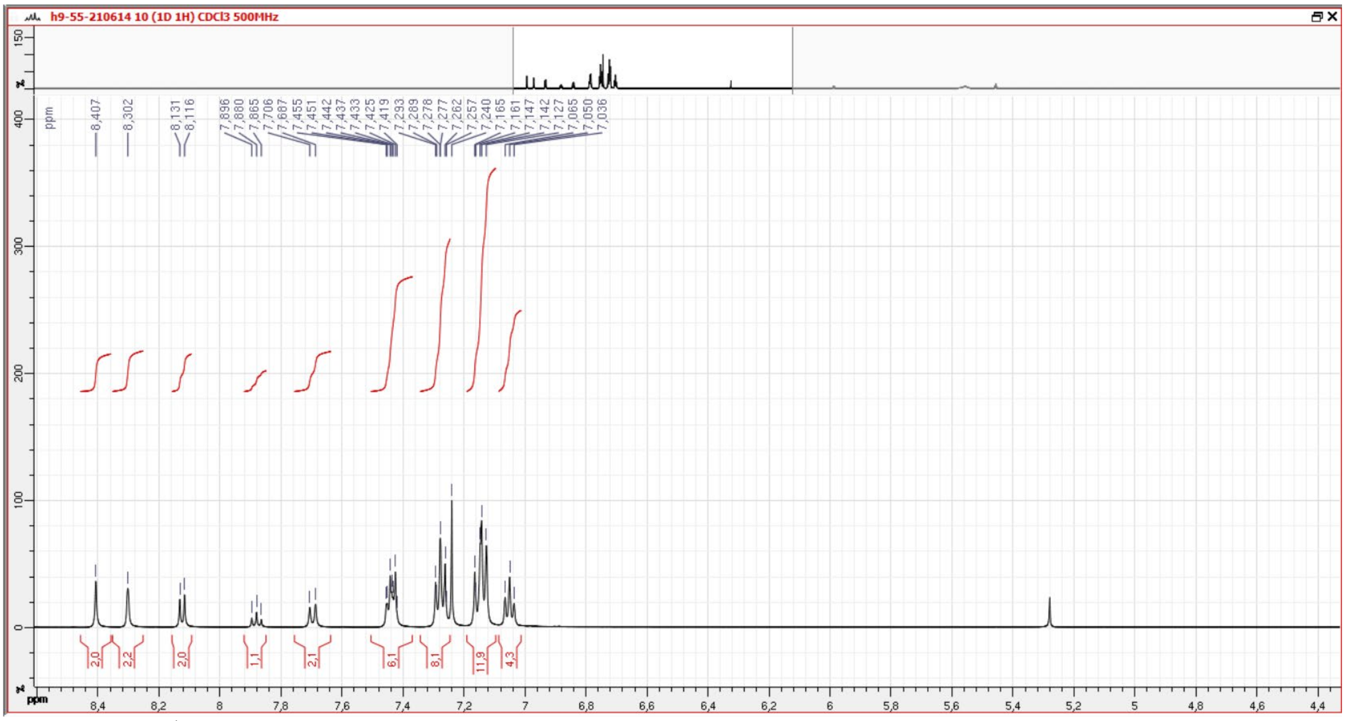

Figure S27. ^{1}H NMR (500 MHz, 298K) spectrum of compound **5c** in $CDCl_3$.

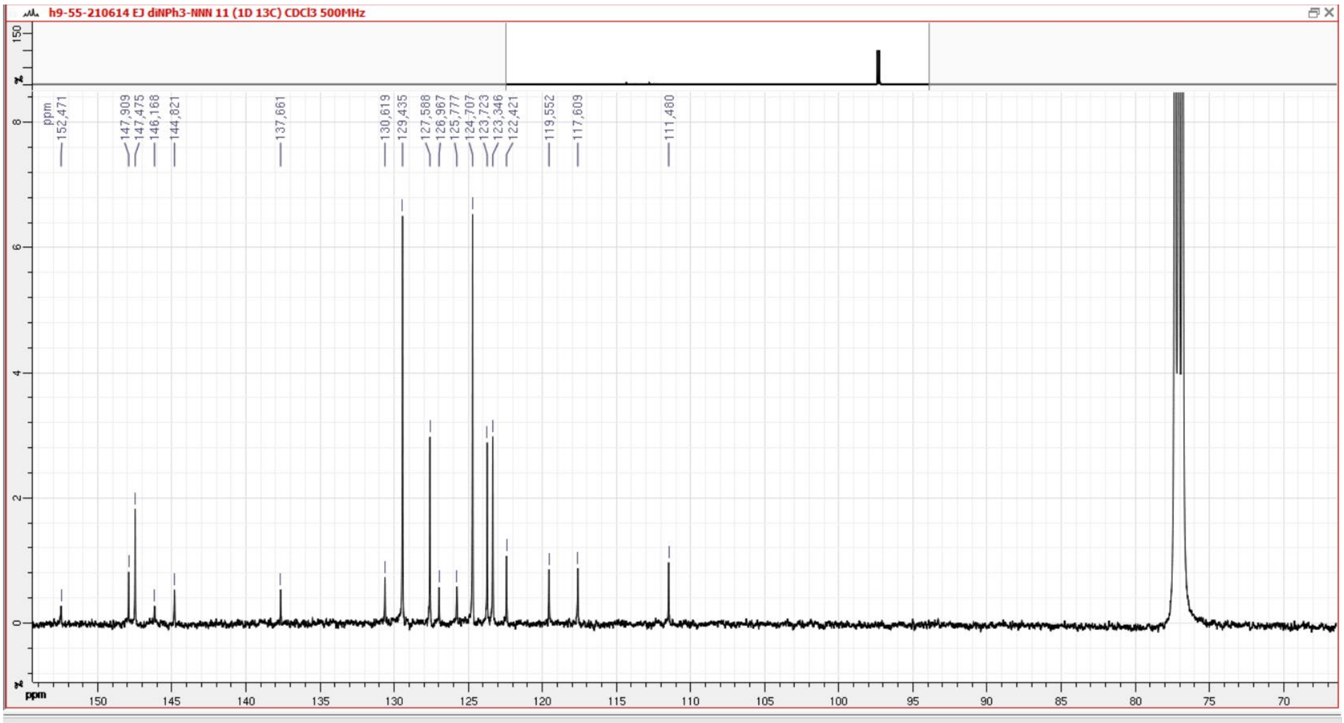

Figure S28. ^{13}C NMR (125 MHz, 298K) spectrum of compound **5c** in $CDCl_3$.

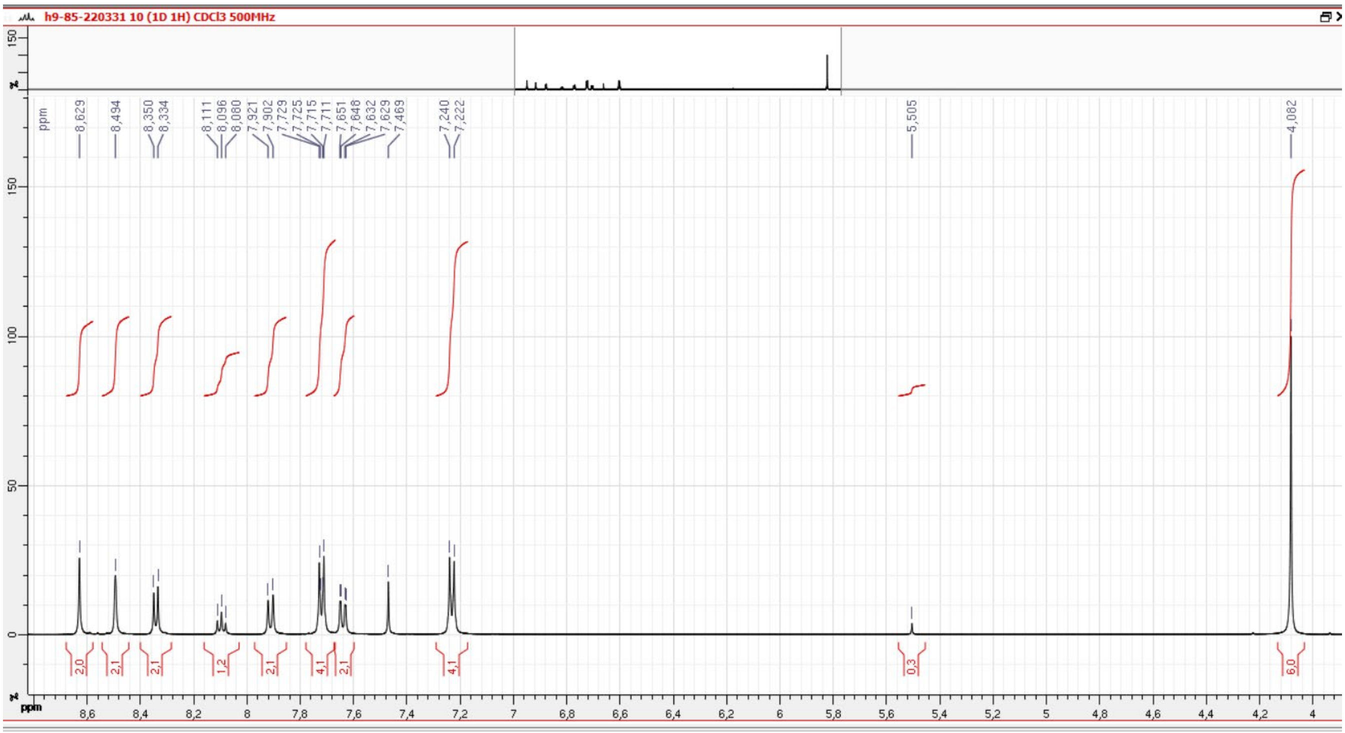

Figure S29. ^{1}H NMR (500 MHz, 298K) spectrum of compound **5d** in $CDCl_3$.

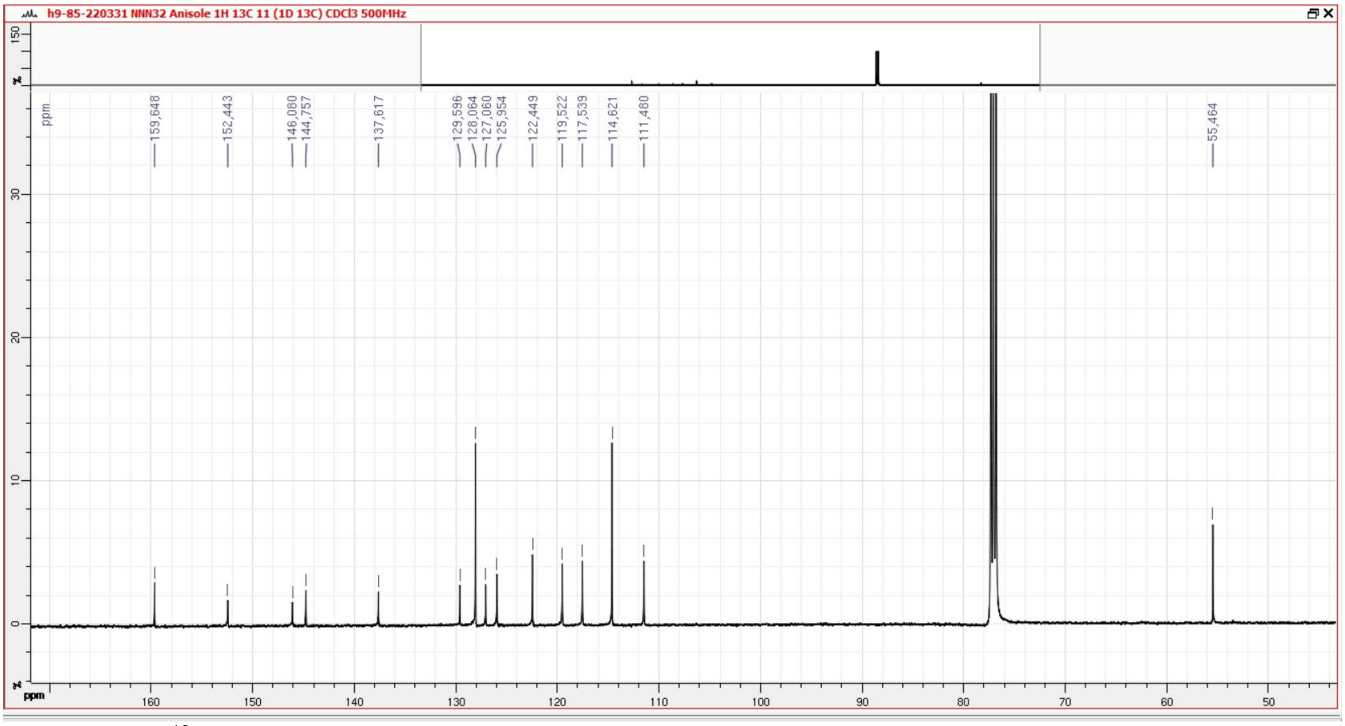

Figure S30. ^{13}C NMR (125 MHz, 298K) spectrum of compound **5d** in $CDCl_3$.

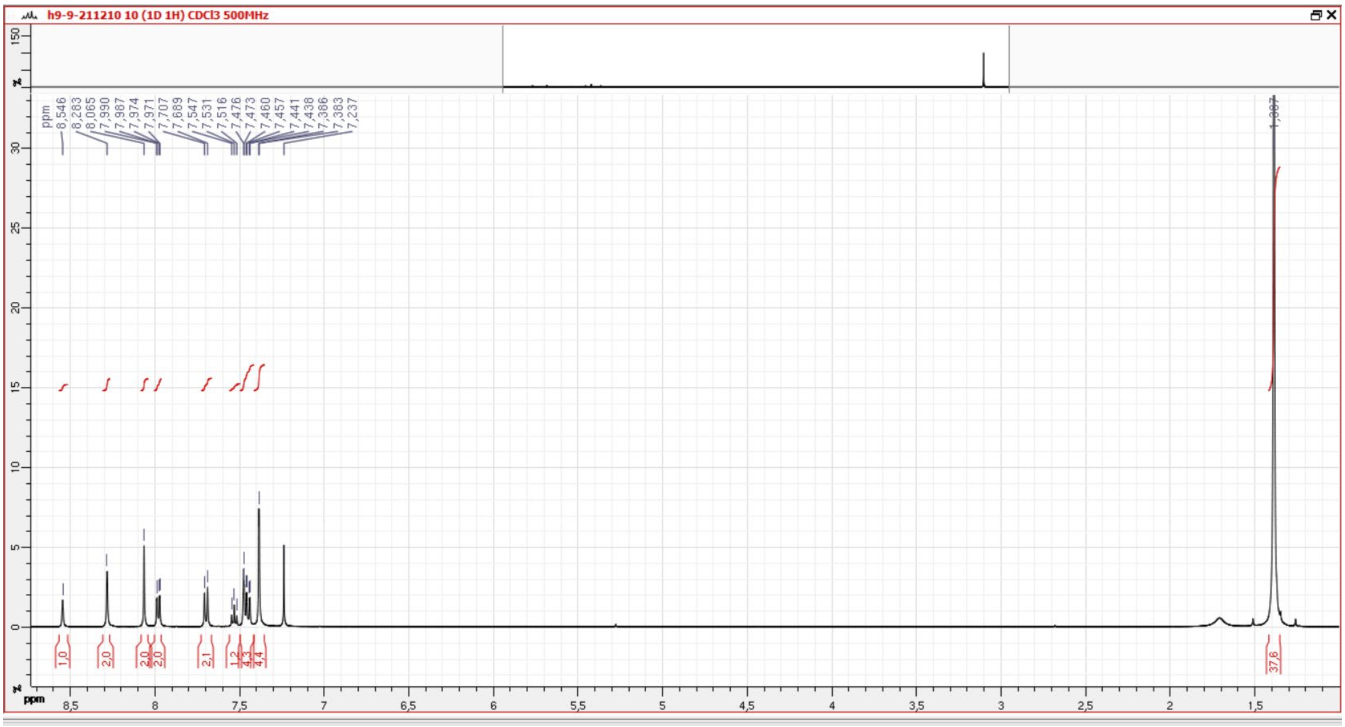

Figure S31. ^{1}H NMR (500 MHz, 298K) spectrum of compound **5e** in $CDCl_3$.

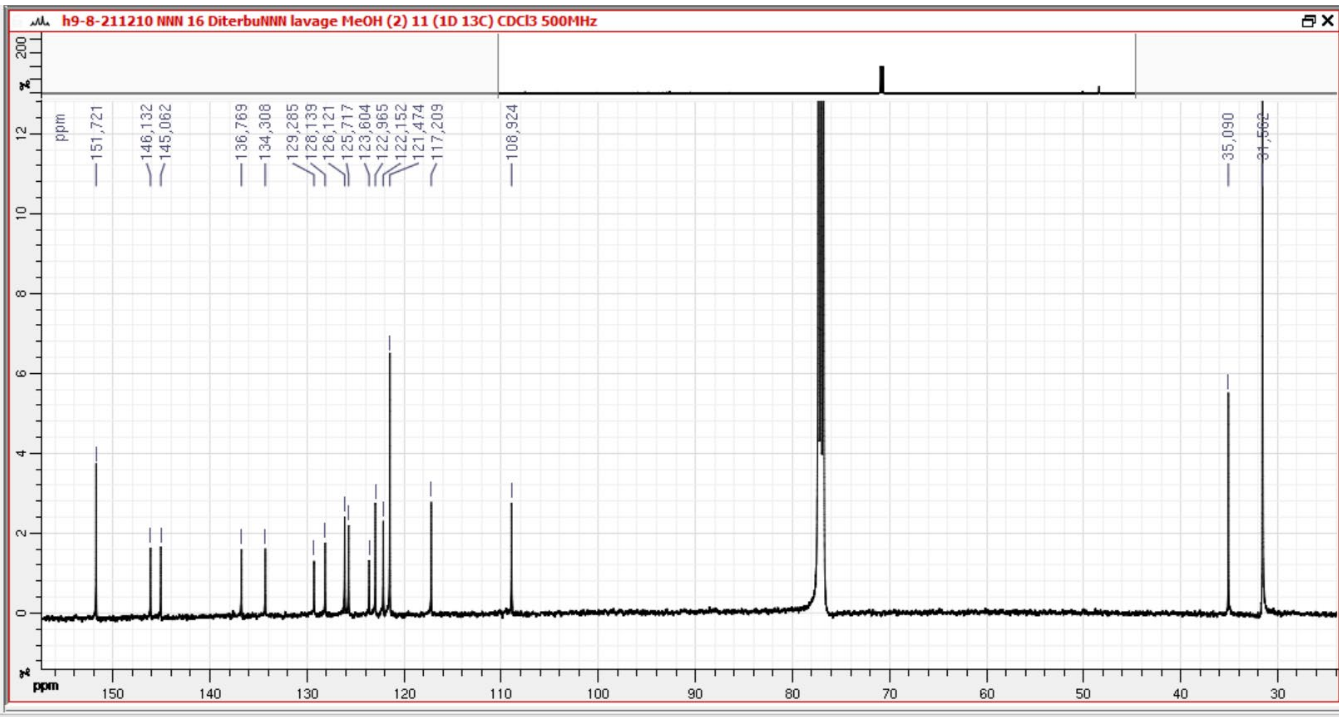

Figure S32. ^{13}C NMR (125 MHz, 298K) spectrum of compound **5e** in $CDCl_3$.

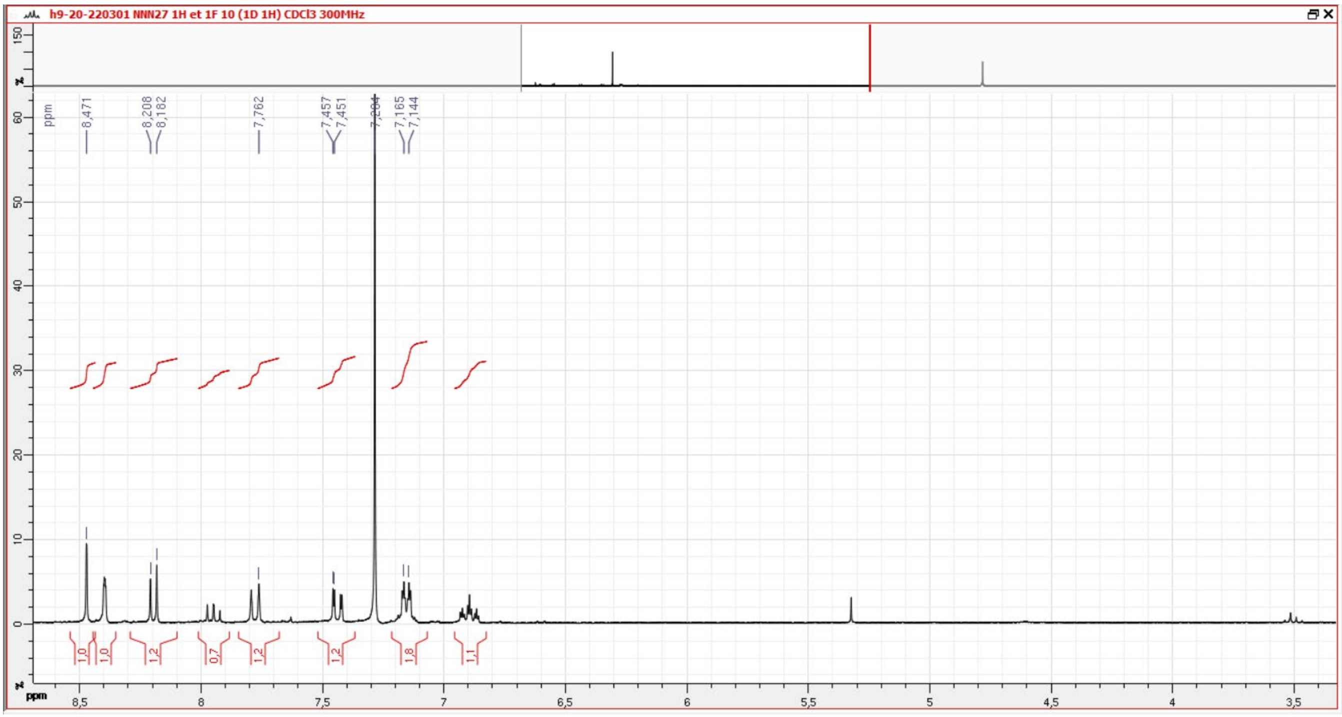

Figure S33. ^{1}H NMR (500 MHz, 298K) spectrum of compound **5f** in $CDCl_3$.

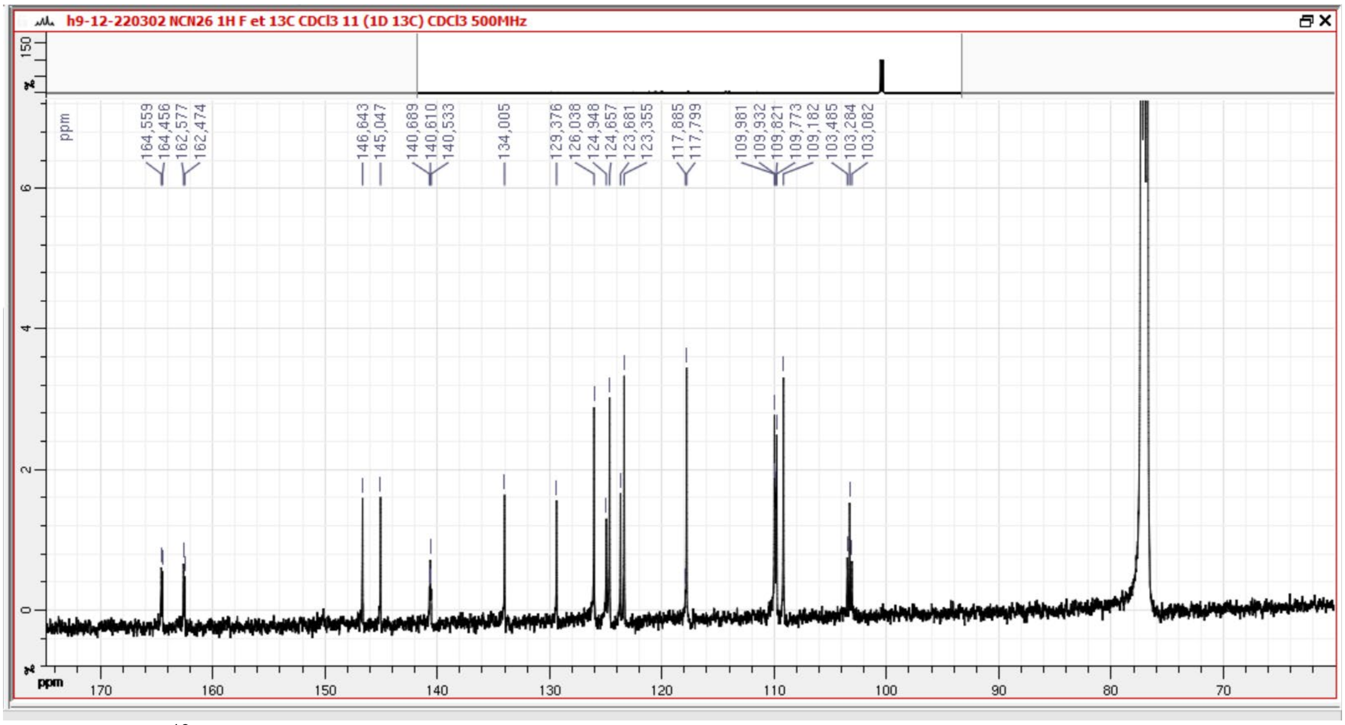

Figure S34. ^{13}C NMR (125 MHz, 298K) spectrum of compound **5f** in $CDCl_3$.

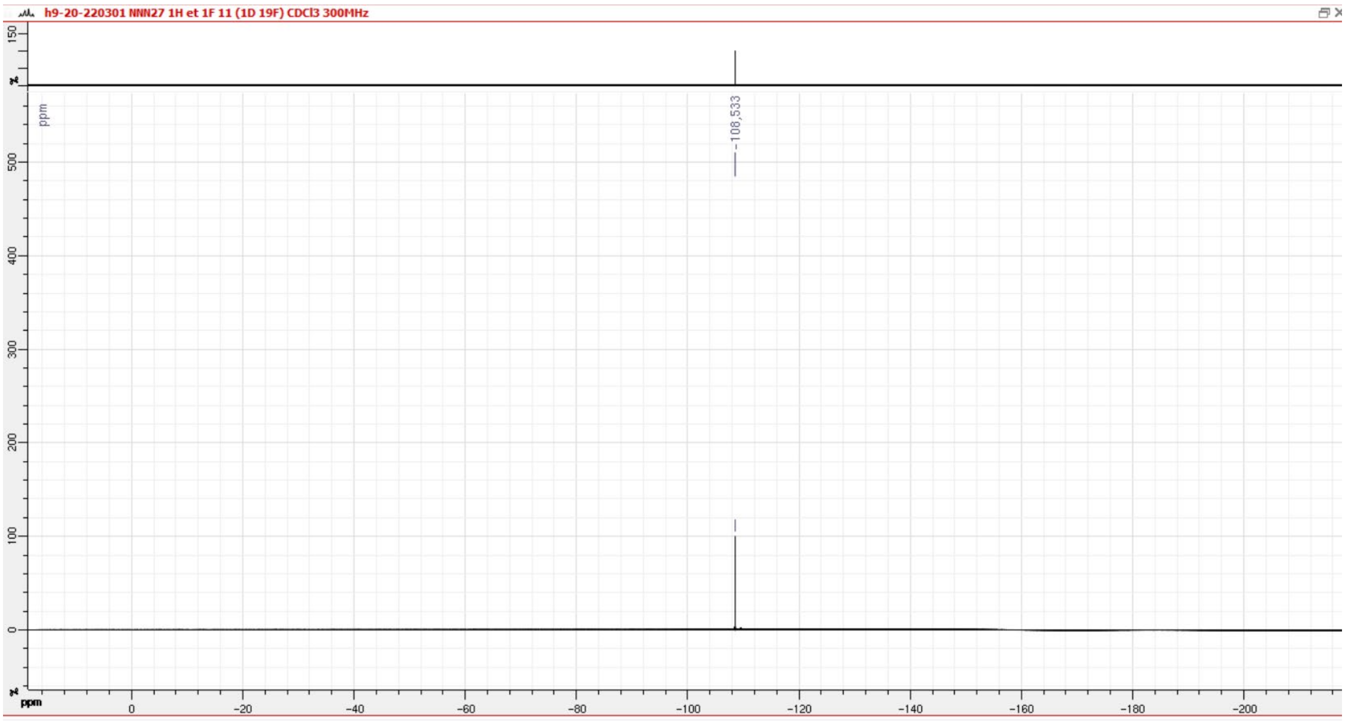

Figure S35. ^{19}F NMR (282 MHz, 298K) spectrum of compound **5f** in $CDCl_3$.

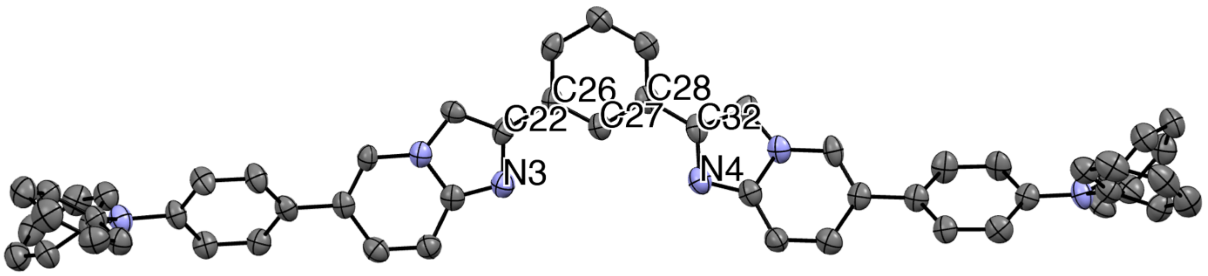

Figure S36. ORTEP diagram of compound **4c·CH_2Cl_2** with partial atom numbering. Gray and blue ellipsoids represent carbon and nitrogen atoms, respectively.

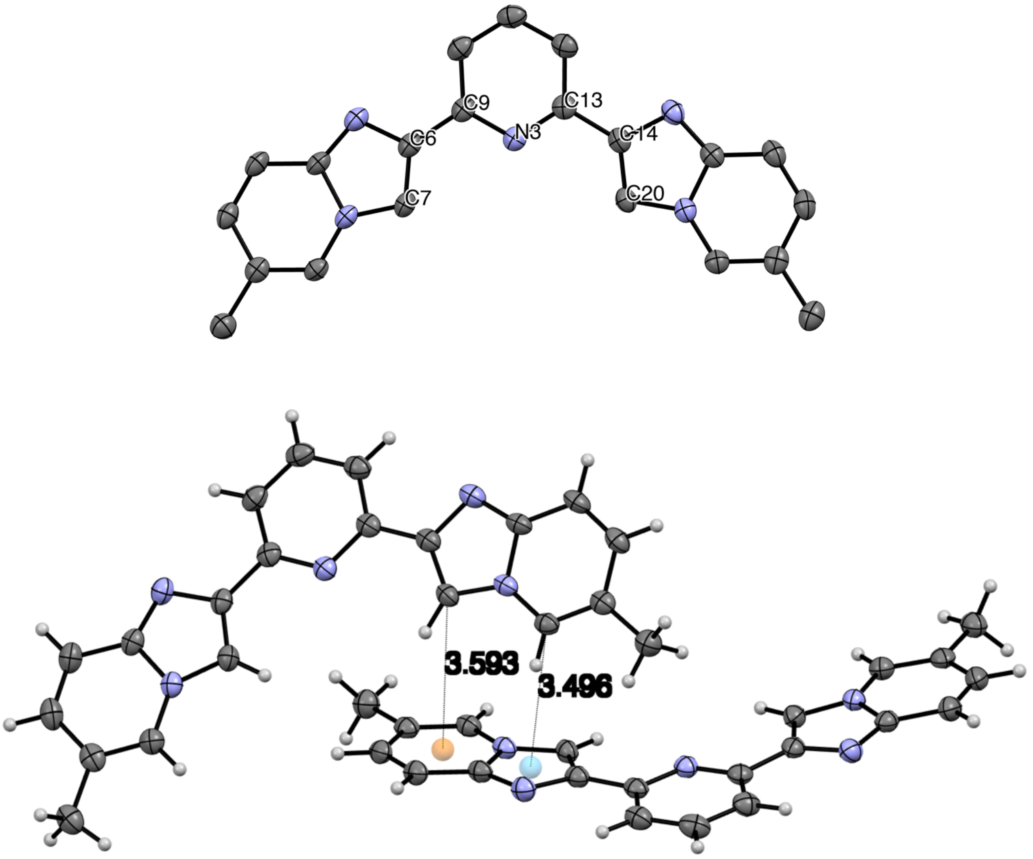

Figure S37. ORTEP diagram of compound **5a** with partial atom numbering (top) and partial crystal packing that highlights the two intermolecular C–H···p interactions (bottom). Gray and blue ellipsoids represent carbon and nitrogen atoms, respectively.

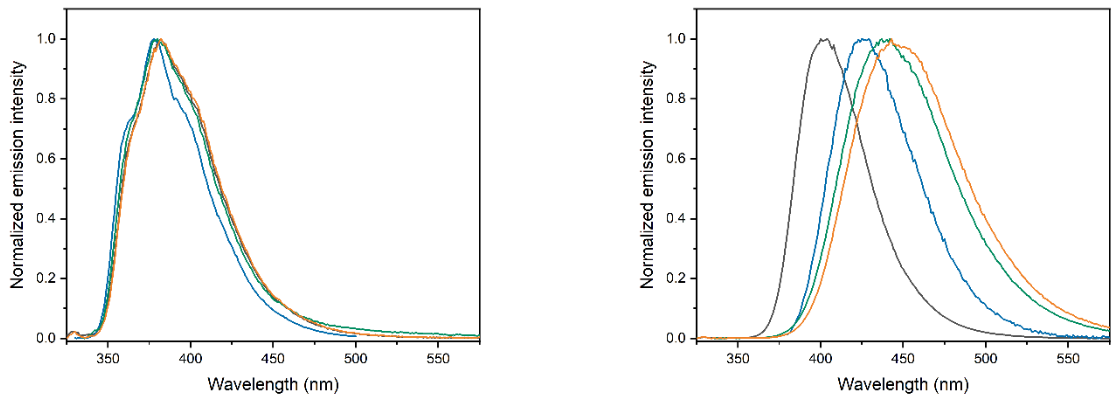

Figure S38. Solvent effect on the photoluminescence spectra recorded for dilute samples of compound **NCN01** (*left box*) and **NCN18** (*right box*) upon excitation at λ_{exc} = 300–320 nm. Solvent: dioxane (black traces), CH_2Cl_2 (blue traces), DMF (green traces) and DMSO (orange traces).

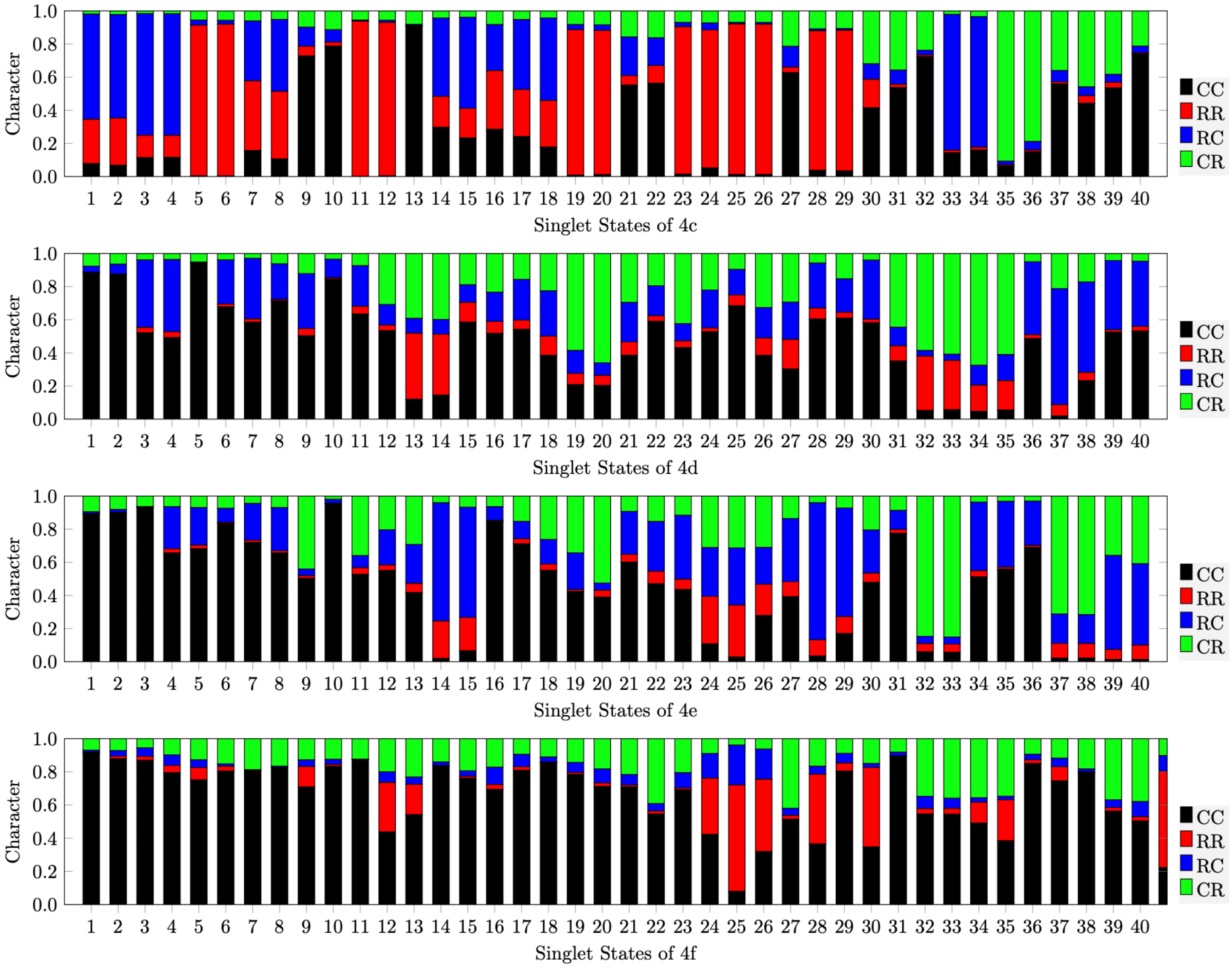

Figure S39. THEODore analyses of the absorption spectra of the different ligand. C stands for the *bis*-imidazo[1,2-*a*]pyridine-phenyl core moiety and R for the methyl substituent (in **4c**) or the peripheral substituted phenyls (for **4d-4f**).

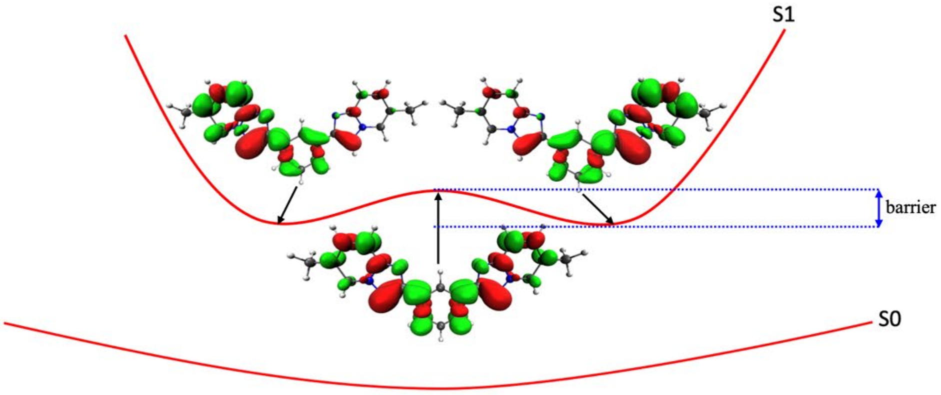

Figure S40. Schematic representation of the two minima of **4b** on the S1 Potential Energy Surface.

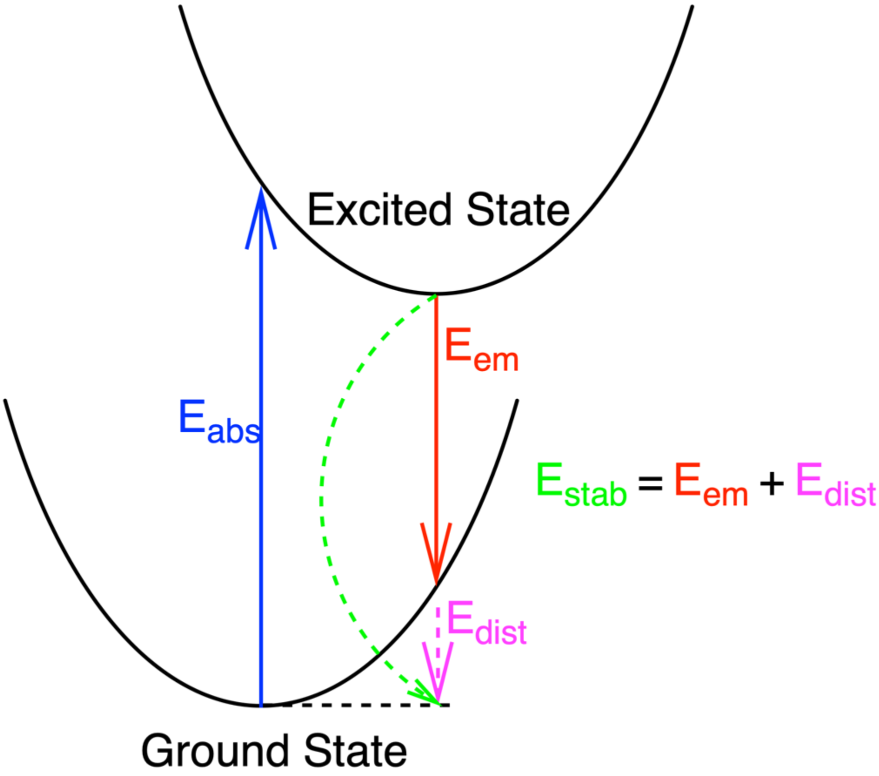

Figure S41. Energetic diagram defining the E_{abs}, E_{dist}, E_{em} and E_{stab} energies.

Table S1. Sample and crystal data for compound **4c·CH_2Cl_2** (CCDC 2183609).

**Identification code**	e4996	
**Chemical formula**	$C_{57}H_{42}Cl_2N_6$	
**Formula weight**	881.86 g/mol	
**Temperature**	173(2) K	
**Wavelength**	0.71073 Å	
**Crystal size**	0.100 x 0.120 x 0.120 mm	
**Crystal system**	triclinic	
**Space group**	P -1	
**Unit cell dimensions**	a = 10.691(3) Å	α = 83.622(6)°
	b = 12.043(4) Å	β = 80.897(10)°
	c = 18.629(5) Å	γ = 70.437(7)°
**Volume**	2227.2(11) $Å^3$	
**Z**	2	
**Density (calculated)**	1.315 g/cm^3	
**Absorption coefficient**	0.194 mm^{-1}	
**F(000)**	920	

Table S2. Data collection and structure refinement for **4c·CH_2Cl_2** (CCDC 2183609).

**Theta range for data collection**	1.80 to 27.56°	
**Index ranges**	-13<=*h*<=13, -15<=*k*<=15, -24<=*l*<=22	
**Reflections collected**	16339	
**Independent reflections**	9620 [R(int) = 0.1081]	
**Max. and min. transmission**	0.7651 and 0.6457	
**Structure solution technique**	direct methods	
**Structure solution program**	SHELXT 2014/5 (Sheldrick, 2014)	
**Refinement method**	Full-matrix least-squares on F^2	
**Refinement program**	SHELXL-2018/3 (Sheldrick, 2018)	
**Function minimized**	Σ w(F_o^2 - F_c^2)^2	
**Data / restraints / parameters**	9620 / 0 / 586	
**Goodness-of-fit on F^2**	0.982	
**Final R indices**	3109 data; I>2σ(I)	R1 = 0.0928, wR2 = 0.1699
	all data	R1 = 0.2814, wR2 = 0.2370
**Weighting scheme**	w=1/[σ^2(F_o^2)+(0.0726P)^2]<br>where P=(F_o^2+2F_c^2)/3	
**Largest diff. peak and hole**	0.708 and -0.364 e$Å^{-3}$	
**R.M.S. deviation from mean**	0.055 e$Å^{-3}$	

Table S3. Sample and crystal data for compound **5a** (CCDC 2183608).

**Identification code**	e4994	
**Chemical formula**	$C_{21}H_{17}N_5$	
**Formula weight**	339.40 g/mol	
**Temperature**	173(2) K	
**Wavelength**	0.71073 Å	
**Crystal size**	0.150 x 0.150 x 0.200 mm	
**Crystal system**	monoclinic	
**Space group**	P 1 21/n 1	
**Unit cell dimensions**	a = 6.7209(3) Å	α = 90°
	b = 7.3022(4) Å	β = 94.324(2)°
	c = 34.0422(18) Å	γ = 90°
**Volume**	1665.95(15) $Å^3$	
**Z**	4	
**Density (calculated)**	1.353 g/cm^3	
**Absorption coefficient**	0.084 mm^{-1}	
***F*(000)**	712	

Table S4. Data collection and structure refinement for **5a** (CCDC 2183608).

**Theta range for data collection**	2.40 to 27.89°	
**Index ranges**	-8<=h<=8, -9<=k<=9, -44<=l<=43	
**Reflections collected**	15613	
**Independent reflections**	3843 [R(int) = 0.0465]	
**Max. and min. transmission**	0.7510 and 0.6540	
**Structure solution technique**	direct methods	
**Structure solution program**	SHELXT 2014/5 (Sheldrick, 2014)	
**Refinement method**	Full-matrix least-squares on F^2	
**Refinement program**	SHELXL-2018/3 (Sheldrick, 2018)	
**Function minimized**	Σ w(F_o^2 - F_c^2)^2	
**Data / restraints / parameters**	3843 / 0 / 237	
**Goodness-of-fit on F^2**	1.020	
**Δ/σ_{max}**	0.001	
**Final R indices**	2983 data; I>2σ(I)	R1 = 0.0441, wR2 = 0.1059
	all data	R1 = 0.0595, wR2 = 0.1167
**Weighting scheme**	w=1/[σ^2(F_o^2)+(0.0511P)^2+0.4835P]<br>where P=(F_o^2+2F_c^2)/3	
**Largest diff. peak and hole**	0.161 and -0.274 e$Å^{-3}$	
**R.M.S. deviation from mean**	0.049 e$Å^{-3}$	

Table S5. Photophysical data recorded for samples of compounds **4b** and **4c** in dilute solution of solvent with different polarity.

**compound**	*Solvent*	λ_{abs} (ε) [nm, 10^4 M^{-1} cm^{-1}]	λ_{em} [nm]	PLQY
**4b**	*DMSO*	329 (2.2)<br>345 (1.6)	365, 382, 404*sh*	0.25
	DMF	294 (3.5)<br>330 (3.7)<br>340 (3.3)	365, 382, 404*sh*	0.21
	CH_2Cl_2	251 (6.17)<br>316 (1.76)<br>327 (1.76)	363, 378, 395*sh*	0.41
	dioxane	330 (2.1)<br>345 (1.4)	364, 380, 400*sh*	0.20
**4c**	*DMSO*	312 (6.4)<br>340 (7.0)	447	0.51
	DMF	312 (6.5)<br>340 (7.2)	440	0.52
	CH_2Cl_2	253 (4.69)<br>310 (4.30)<br>332 (4.50)	427	0.50
	dioxane	272 (7.1)<br>310 (6.3)<br>341 (7.2)	402	0.36

Table S6. Energetic barrier between the two minima of the S_1 PES. Data are listed in eV.

	4b	4c	4d	4e	4f
barrier	0.048	0.093	0.067	0.039	0.096

Table S7. E_{dist}, E_{em}, E_{stab}, ΔE_{stab} energies (in eV) and emission wavelength (λ_{em} in nm) computed for the different conformer, symmetry group and state for **4b**. In red, the values retained in the main text. See definition in Figure S40.

Conformer	In-In	In-In	In-In	In-In	In-Out	Out-Out	Out-Out	Out-Out
Symmetry	C1	C1	C2	C2	C1	C1	C2	C2
State	S1a	90S1a	S1a	S1b	S1a	S1a	s1a	S1b
E_{dist}	0.207	0.371	0.162	0.135	0.199	0.208	0.134	0.149
E_{em}	3.422	3.444	3.515	3.575	3.428	3.450	3.648	3.537
E_{stab}	3.630	3.815	3.677	3.710	3.628	3.658	3.781	3.686
ΔE_{stab}	0.002	0.187	0.050	0.082	0.000	0.030	0.154	0.059
λ_{em}	362	360	353	347	362	359	340	351

Table S8. E_{dist}, E_{em}, E_{stab}, ΔE_{stab} energies (in eV) and emission wavelength (λ_{em} in nm) computed for the different conformer, symmetry group and state for **5b**. In red, the values retained in the main text.

Conformer	In-In	Out-Out	Out-Out	Out-Out
Symmetry	C1	C1	C2	C2
State	S1a	S1a	S1a	S1b
E_{dist}	0.377	0.181	0.135	0.179
E_{em}	3.398	3.414	3.623	3.415
E_{stab}	3.776	3.595	3.759	3.594
ΔE_{stab}	0.181	0.000	0.164	0.000
λ_{em}	365	363	342	363

Table S9. E_{dist}, E_{em}, E_{stab}, ΔE_{stab} energies (in eV) and emission wavelength (λ_{em} in nm) computed for the different conformer, symmetry group and state for **4c** and **5c**. In red, the values retained in the main text.

	4c				5c
Conformer	In-In	In-In	In-In		Out-Out
Symmetry	C1	Cs	Cs		C1
State	S1a	S1a’	S1a’’		S1a
E_{dist}	0.180	0.099	0.102		0.181
E_{em}	2.865	3.056	3.036		2.833
E_{stab}	3.045	3.155	3.138		3.014
ΔE_{stab}	0.000	0.110	0.093		0.000
λ_{em}	433	406	408		438

Table S10. E_{dist}, E_{em}, E_{stab}, ΔE_{stab} energies (in eV) and emission wavelength (λ_{em} in nm) computed for the different conformer, symmetry group and state for **4d**. In red, the values retained in the main text. a) this state is the charge transfer state from the anisole R group to the core.

Conformer	In-In	In-In	In-In	In-In	Out-Out
Symmetry	C1	C1	C2	C2	C1
State	S1a	NS1a[a]	S1a	S1b	S1a
E_{dist}	0.226	0.301	0.147	0.151	0.228
E_{em}	3.298	3.249	3.444	3.462	3.317
E_{stab}	3.524	3.551	3.591	3.613	3.545
ΔE_{stab}	0.000	0.027	0.067	0.089	0.021
λ_{em}	376	382	360	358	374

Table S11. E_{dist}, E_{em}, E_{stab}, ΔE_{stab} energies (in eV) and emission wavelength (λ_{em} in nm) computed for the different conformer, symmetry group and state for **5d**. In red, the values retained in the main text. a) this state is the charge transfer state towards the anisole R group.

Conformer	In-In	In-In	In-Out	Out-Out	Out-Out
Symmetry	C1	C1	C1	C1	C1
State	S1a	NS1a[a]	S1a	S1a	NS1a[a]
E_{dist}	0.415	0.571	0.425	0.423	0.312
E_{em}	3.284	3.168	3.186	3.279	3.183
E_{stab}	3.700	3.739	3.611	3.701	3.495
ΔE_{stab}	0.197	0.235	0.108	0.206	0.000
λ_{em}	377	391	389	378	389

Table S12. E_{dist}, E_{em}, E_{stab}, ΔE_{stab} energies (in eV) and emission wavelength (λ_{em} in nm) computed for the different conformer, symmetry group and state for **4e** and **5e**. In red, the values retained in the main text.

	4e				5e
Conformer	In-In	In-In	In-In		Out-Out
Symmetry	C1	Cs	Cs		C1
State	S1a	S1a′	S1a′′		s1a
E_{dist}	0.238	0.148	0.167		0.162
E_{em}	3.309	3.439	3.472		3.369
E_{stab}	3.547	3.587	3.639		3.531
ΔE_{stab}	0.000	0.039	0.091		0.000
λ_{em}	375	361	357		368

Table S13. E_{dist}, E_{em}, E_{stab}, ΔE_{stab} energies (in eV) and emission wavelength (λ_{em} in nm) computed for the different conformer, symmetry group and state for **4f** and **5f**. In red, the values retained in the main text. NS1 states are charge transfer states. a) this is a conformer.

	4f				5f			
Conformer	In-In	In-In	In-In		Out-Out	Out-Out	Out-Out	Out-Out
Symmetry	C1	Cs	Cs		C1	C1	C2	C2
State	NS1a	NS1a′	NS1a′′		NS1a	NNS1a[a]	NS1a	NS1b

E_{dist}	0.281	0.152	0.159		0.156	0.293	0.139	0.157
E_{em}	3.126	3.351	3.373		3.331	3.155	3.475	3.333
E_{stab}	3.407	3.503	3.532		3.487	3.448	3.614	3.489
ΔE_{stab}	0.000	0.096	0.125		0.039	0.000	0.166	0.041
λ_{em}	397	370	368		372	393	357	372

Table S14. Absorption energy (E_{abs} in eV), absorption wavelength (λ_{abs} in nm) and oscillator strength (f_{osc}) computed for the absorption spectra of **4b** and **5b**.

**4b**			**5b**		
E_{abs}	λ_{abs}	f_{osc}	E_{abs}	λ_{abs}	f_{osc}
3.847	322	6.67E-01	3.776	328	5.31E-01
3.923	316	4.25E-01	3.898	318	4.68E-01
4.029	308	3.14E-01	3.932	315	1.64E-01
4.193	296	1.78E-03	4.001	310	1.11E-02
4.255	291	4.66E-03	4.202	295	5.90E-01
4.391	282	7.94E-02	4.249	292	6.19E-03
4.424	280	3.24E-01	4.433	280	1.07E-01
4.523	274	1.92E-01	4.500	276	8.81E-02
4.525	274	4.83E-02	4.541	273	1.62E-01
4.685	265	8.60E-01	4.688	264	4.22E-01
4.741	262	2.52E-03	4.734	262	7.30E-02
4.831	257	4.28E-02	4.768	260	6.75E-03
4.861	255	3.70E-01	4.821	257	2.77E-02
4.928	252	2.34E-01	4.859	255	5.67E-05
5.097	243	1.52E-02	4.866	255	4.95E-05
5.123	242	4.97E-01	4.930	252	6.19E-01
5.329	233	3.39E-02	5.130	242	2.07E-03
5.359	231	2.78E-01	5.225	237	7.41E-01
5.464	227	4.89E-02	5.448	228	6.01E-02
5.549	223	2.72E-02	5.535	224	1.57E-03

Table S15. Absorption energy (E_{abs} in eV), absorption wavelength (λ_{abs} in nm) and oscillator strength (f_{osc}) computed for the absorption spectra of **4c** and **5c**.

**4c**			**5c**		
E_{abs}	λ_{abs}	f_{osc}	E_{abs}	λ_{abs}	$\mathbf{f}_{osc}$
3.288	377	9.37E-01	3.211	386	5.60E-01
3.321	373	3.59E-01	3.229	384	6.31E-01
3.555	349	5.73E-02	3.400	365	2.88E-02
3.584	346	6.11E-03	3.404	364	2.18E-02
3.652	340	8.40E-02	3.652	340	8.41E-02

3.656	339	7.58E-02	3.654	339	6.56E-02
3.718	334	1.71E-01	3.699	335	1.38E-01
3.726	333	4.19E-01	3.720	333	7.19E-01
3.764	329	2.57E-01	3.727	333	3.63E-01
3.806	326	6.90E-01	3.786	327	4.58E-01
3.851	322	3.91E-01	3.834	323	1.25E-01
3.855	322	4.68E-01	3.842	323	1.80E-01
3.960	313	1.39E-02	3.847	322	4.79E-01
4.086	303	1.36E-02	3.920	316	4.71E-02
4.107	302	5.41E-02	3.936	315	1.10E-01
4.126	300	9.40E-03	3.963	313	2.13E-02
4.146	299	7.57E-02	4.094	303	7.23E-03
4.180	297	2.80E-02	4.099	302	1.28E-03
4.220	294	4.65E-03	4.167	298	6.28E-02
4.223	294	4.46E-03	4.168	297	7.72E-02
4.254	291	1.70E-01	4.174	297	4.03E-01
4.294	289	1.38E-01	4.198	295	4.80E-02
4.303	288	8.70E-02	4.275	290	1.86E-02
4.305	288	1.69E-02	4.281	290	4.53E-02
4.359	284	9.15E-02	4.314	287	3.43E-02
4.364	284	7.97E-02	4.316	287	4.97E-02
4.382	283	2.27E-01	4.346	285	5.11E-02
4.400	282	4.86E-02	4.348	285	1.51E-03
4.402	282	4.00E-02	4.351	285	7.51E-05
4.408	281	3.75E-01	4.357	285	5.32E-02
4.446	279	2.92E-01	4.378	283	1.75E-03
4.514	275	5.18E-02	4.452	279	1.05E-02
4.532	274	2.10E-03	4.457	278	4.35E-03
4.534	273	8.04E-03	4.502	275	6.12E-02
4.547	273	7.04E-03	4.542	273	8.99E-01
4.560	272	2.28E-02	4.600	269	2.48E-01
4.571	271	5.24E-02	4.635	267	1.39E-03
4.657	266	2.13E-01	4.637	267	1.44E-03
4.669	266	2.24E-01	4.658	266	2.57E-03
4.703	264	1.03E-02	4.694	264	2.26E-01

Table S16. Absorption energy (E_{abs} in eV), absorption wavelength (λ_{abs} in nm) and oscillator strength (f_{osc}) computed for the absorption spectra of **4d** and **5d**.

4d			5d		
E_{abs}	λ_{abs}	f_{osc}	E_{abs}	λ_{abs}	f_{osc}
3.739	332	2.26E-01	3.634	341	1.31E+00
3.752	330	1.18E+00	3.735	332	1.19E-01
3.882	319	4.82E-01	3.832	324	1.90E-01
3.923	316	1.76E-01	3.843	323	1.34E-01
3.956	313	1.08E-02	3.895	318	7.11E-04
4.087	303	1.74E-02	3.938	315	1.02E-01
4.097	303	1.17E-01	4.086	303	1.88E-01

4.189	296	1.14E-04	4.145	299	7.79E-04
4.452	278	1.89E+00	4.421	280	1.43E+00
4.469	277	1.39E-02	4.467	278	1.42E-02
4.520	274	1.82E-01	4.487	276	1.51E-01
4.558	272	7.94E-02	4.516	275	1.25E-02
4.609	269	3.76E-02	4.572	271	2.90E-04
4.616	269	1.96E-02	4.603	269	1.77E-03
4.659	266	2.14E-02	4.605	269	3.38E-03
4.664	266	2.77E-02	4.622	268	1.87E-01
4.726	262	3.33E-01	4.643	267	1.66E-02
4.737	262	1.16E-01	4.667	266	4.42E-02
4.771	260	1.28E-01	4.709	263	6.49E-01
4.772	260	1.50E-01	4.720	263	7.39E-02
4.784	259	1.73E-01	4.723	262	1.40E-02
4.801	258	1.58E-02	4.769	260	1.49E-01
4.810	258	2.07E-01	4.811	258	1.94E-01
4.918	252	3.19E-02	4.815	258	2.17E-01
4.944	251	8.01E-04	4.836	256	2.23E-02
4.955	250	7.38E-02	4.851	256	8.02E-02
4.972	249	1.79E-02	4.869	255	2.31E-02
4.981	249	1.17E-03	4.945	251	1.32E-02
5.005	248	1.31E-02	4.963	250	2.13E-02
5.014	247	4.87E-02	5.004	248	2.61E-01
5.058	245	6.63E-03	5.009	248	2.41E-02
5.099	243	2.84E-03	5.061	245	2.01E-02
5.102	243	3.85E-03	5.107	243	4.16E-03
5.146	241	8.46E-03	5.109	243	3.31E-03
5.153	241	2.00E-03	5.131	242	1.79E-02
5.205	238	1.00E-02	5.137	241	2.96E-02
5.221	237	2.62E-02	5.169	240	2.52E-01
5.227	237	1.02E-01	5.202	238	6.22E-03
5.363	231	6.27E-03	5.278	235	7.27E-03
5.369	231	1.73E-01	5.284	235	9.79E-03

Table S17. Absorption energy (E_{abs} in eV), absorption wavelength (λ_{abs} in nm) and oscillator strength (f_{osc}) computed for the absorption spectra of **4e** and **5e**.

**4e**			**5e**		
E_{abs}	λ_{abs}	f_{osc}	E_{abs}	λ_{abs}	f_{osc}
3.721	333	1.10E-01	3.701	335	6.85E-01
3.740	331	1.05E+00	3.814	325	3.62E-01
3.942	315	3.13E-02	3.873	320	2.55E-02
4.005	310	7.59E-01	3.924	316	1.06E-01
4.044	307	2.16E-01	3.992	311	6.04E-01
4.126	300	6.14E-02	4.007	309	9.84E-02
4.203	295	5.54E-02	4.208	295	1.24E-01
4.276	290	2.39E-03	4.321	287	2.67E-02
4.446	279	1.22E+00	4.415	281	2.49E-02
4.469	277	1.58E-03	4.471	277	1.60E-01

4.555	272	4.08E-01	4.499	276	6.91E-01
4.580	271	7.30E-01	4.581	271	8.85E-01
4.633	268	8.94E-03	4.602	269	7.99E-03
4.648	267	1.06E-02	4.602	269	7.95E-03
4.650	267	1.27E-02	4.656	266	4.41E-01
4.685	265	7.25E-03	4.727	262	1.49E-01
4.750	261	3.19E-01	4.734	262	6.14E-02
4.765	260	5.77E-03	4.779	259	4.81E-02
4.868	255	1.36E-03	4.798	258	2.29E-01
4.896	253	8.78E-04	4.828	257	1.10E-02
4.897	253	8.43E-06	4.840	256	5.55E-02
4.944	251	7.26E-02	4.885	254	8.26E-02
4.958	250	5.37E-02	4.891	254	6.16E-03
4.982	249	3.20E-02	4.891	254	5.43E-04
4.987	249	1.14E-03	4.902	253	5.23E-02
5.007	248	2.85E-02	4.904	253	3.08E-02
5.018	247	1.63E-04	4.909	253	2.42E-02
5.021	247	8.82E-04	4.956	250	5.13E-03
5.023	247	6.96E-04	4.975	249	3.94E-03
5.052	245	2.54E-02	5.006	248	6.65E-03
5.064	245	1.80E-03	5.007	248	5.42E-03
5.088	244	5.03E-04	5.076	244	1.40E-02
5.088	244	4.87E-04	5.172	240	3.95E-03
5.139	241	1.86E-02	5.192	239	2.64E-03
5.168	240	1.22E-01	5.195	239	3.25E-03
5.318	233	4.64E-02	5.231	237	1.14E-01
5.404	229	1.88E-02	5.242	237	5.73E-02
5.404	229	5.47E-02	5.302	234	1.71E-02
5.498	226	2.05E-02	5.325	233	6.31E-03
5.500	225	2.25E-02	5.326	233	9.89E-03

Table S18. Absorption energy (E_{abs} in eV), absorption wavelength (λ_{abs} in nm) and oscillator strength (f_{osc}) computed for the absorption spectra of **4f** and **5f**.

**4f**			**5f**		
E_{abs}	λ_{abs}	f_{osc}	E_{abs}	λ_{abs}	f_{osc}
3.663	338	8.40E-02	3.649	340	5.61E-01
3.680	337	8.78E-01	3.752	330	3.33E-01
3.899	318	6.39E-03	3.831	324	2.63E-04
4.040	307	9.94E-02	3.897	318	9.89E-02
4.045	307	8.73E-01	4.022	308	8.63E-01
4.128	300	1.56E-01	4.074	304	1.38E-01
4.245	292	3.18E-01	4.309	288	5.27E-03
4.274	290	8.48E-01	4.364	284	1.10E-01
4.317	287	1.00E-01	4.408	281	2.97E-01
4.409	281	3.70E-01	4.448	279	9.79E-01
4.489	276	6.98E-03	4.474	277	5.41E-02
4.552	272	5.54E-01	4.507	275	5.93E-01

4.554	272	3.19E-01	4.569	271	1.85E-03
4.682	265	9.18E-02	4.591	270	6.33E-01
4.716	263	1.87E-02	4.641	267	5.02E-02
4.725	262	4.13E-02	4.700	264	5.53E-03
4.774	260	4.37E-02	4.722	263	8.66E-02
4.812	258	3.18E-04	4.781	259	2.79E-03
4.817	257	8.80E-03	4.791	259	4.59E-06
4.835	256	1.97E-02	4.796	259	6.75E-05
4.874	254	4.27E-02	4.797	258	2.39E-02
4.897	253	2.15E-03	4.836	256	1.02E-02
4.900	253	3.60E-03	4.878	254	3.48E-02
4.920	252	4.44E-02	4.971	249	1.36E-01
4.966	250	2.08E-01	4.982	249	7.06E-03
5.110	243	1.66E-02	4.984	249	4.44E-02
5.120	242	1.92E-02	5.074	244	8.65E-03
5.130	242	9.04E-03	5.079	244	7.96E-04
5.132	242	1.30E-02	5.124	242	1.71E-02
5.167	240	4.46E-03	5.127	242	8.00E-03
5.224	237	8.67E-04	5.172	240	3.64E-02
5.291	234	9.80E-05	5.192	239	8.65E-03
5.293	234	4.27E-04	5.192	239	4.54E-03
5.309	234	8.88E-04	5.220	238	9.20E-03
5.311	233	3.04E-04	5.245	236	1.12E-01
5.333	232	2.03E-02	5.284	235	7.99E-03
5.360	231	1.64E-01	5.288	234	5.81E-04
5.434	228	2.56E-04	5.300	234	4.25E-02
5.464	227	2.92E-02	5.467	227	6.78E-02
5.49147	226	3.35E-02	5.469	227	1.69E-02
